# “Expression of concern”: publication bias for positive preclinical cardioprotection studies

**DOI:** 10.1007/s00395-024-01050-4

**Published:** 2024-04-26

**Authors:** Andreas Skyschally, Petra Kleinbongard, Markus Neuhäuser, Gerd Heusch

**Affiliations:** 1 Institute for Pathophysiology, West German Heart and Vascular Center, University Duisburg-Essen, Hufelandstr. 55, 45147 Essen, Germany; 2 Department of Mathematics and Technology, Koblenz University of Applied Sciences, Rhein-Ahr-Campus, Remagen, Germany

**Keywords:** Cardioprotection, Infarct size, Robustness, Translation

## Abstract

The present analysis reports on the robustness of preclinical cardioprotection studies with infarct size as endpoint which were published in Basic Research in Cardiology, Cardiovascular Research, and Circulation Research between January 2013 and December 2023. Only 26 out of 269 papers with technically robust analysis of infarct size by triphenyltetrazolium chloride staining, magnetic resonance imaging or single photon emission tomography applied a prospective power analysis. A retrospective power calculation revealed that only 75% of the reported data sets with statistically significant positive results from all these studies had a statistical power of  ≥ 0.9, and an additional 9% had a statistical power  ≥ 0.8. The remaining 16% of all significant positive data sets did not even reach the 0.8 threshold. Only 13% of all analyzed data sets were neutral. We conclude that neutral studies are underreported and there is indeed a significant lack of robustness in many of the published preclinical cardioprotection studies which may contribute to the difficulties of translating cardioprotection to patient benefit.

**Supplementary Information:**

The online version contains supplementary material available at 10.1007/s00395-024-01050-4.

## Introduction

*In a recent personal experience, we submitted to a prestigious basic science cardiovascular journal a neutral study on infarct size reduction by a pharmacological agent which used a power analysis-based prospective design and did not confirm prior exploratory studies from another laboratory that had reported infarct size reduction by this agent. Three obviously competent expert reviewers appreciated the careful nature of our study and emphasized the need to also publish neutral studies in the field. However, they then went on and asked us to provide a mechanism that would explain the discrepancy between our and the prior studies. We could not identify such mechanism and attributed the discrepancy to lack of* ***robustness*** *of the prior studies, and we defined lack of robustness by the multitude of minor issues in choice of animal strain, anesthesia, surgical preparation, co-medications (anti-arrhythmics, anti-platelets, anti-coagulants), protocol of myocardial ischemia/reperfusion, dosing and timing of the agent, and quantification of infarct size which are specific to one laboratory but not another one. This made two out of three reviewers assign a low priority to our study which was accordingly rejected. We were disappointed, but could also understand that a rigorous defense of a non-significant result raised less enthusiasm in the reviewers than a potentially novel mechanistic finding. On second thought, however, we were concerned: why would we, with a neutral study which was based on a power analysis, used a prospective design and could not reject the null hypothesis, be assigned the burden to explain mechanistically the discrepancy from prior positive studies that were exploratory by nature?*

The field of cardioprotection is faced with a growing disappointment over the translational gap between the myriad of positive preclinical studies and the mostly neutral larger phase III clinical trials [6, 7, 9–11]. By their very nature, preclinical studies are reductionist in that they attempt to identify and characterize mechanism(s) underlying cardioprotection. For that purpose, each laboratory uses a cardioprotective intervention that works well in its hands and can be used to search for mechanism(s). Cardioprotective interventions which do not work are then abandoned, preliminary data are not completed and most often not reported. Only recently, with the recognition of the translational gap, have preclinical studies with neutral results on cardioprotective ischemic conditioning interventions come forward and been reported [17, 18, 20]. The above personal experience led us to suspect that these neutral studies may in fact only be the tip of the iceberg. Could a publication bias for positive preclinical studies on cardioprotective interventions have contributed to unjustified expectations on clinical benefit from these interventions? then to the premature conduct of respective large phase III trials? and finally to the resulting disappointment? We realize that there were a number of so-called “proof-of-concept” clinical phase II trials before embarking on larger phase III trials, but—strictly speaking—these were not designed to provide robust proof for efficacy but for safety of the cardioprotective interventions under study [11].

## Methods

To analyze the potential publication bias for positive preclinical cardioprotection studies, we went back and searched in the publicly accessible Database “Web of Science Core Collection” (Clarivate, Philadelphia, USA) for preclinical studies on cardioprotection (search criteria: “cardioprotection” or “infarct size” or “myocardial infarction”) which were published between January 2013 and December 2023 in Basic Research in Cardiology, Cardiovascular Research, and Circulation Research. In a first refinement step, we excluded all reviews, studies in humans and preclinical studies which did not compare infarct size without and with an intervention. We then used all papers that reported infarct size data without and with an intervention and stratified them as positive vs. neutral/negative according to the reported statistical significance (see Supplementary Table). If multiple data sets with a primary infarct size endpoint were available in a given paper (e.g., for different species, for different sexes), these data sets were separately used in our further analysis (see Supplementary Table: lines marked gray). In a second refinement step, for a more stringent analysis, we used only data sets which had quantified infarct size by either triphenyltetrazolium chloride (TTC) staining (normalized to area at risk, left ventricular or cardiac mass, respectively) or magnetic resonance imaging (MRI) or single photon emission tomography (SPECT) (when calculated in absolute mass or normalized to left ventricular mass). Among all data sets, we identified those with a prospective power analysis (see Supplementary Table: lines marked red) and retrospectively calculated the power of the respective results from the published data sets. In cases where numerical data were not available, we digitized the graphical data and calculated the power from those. Even in prospective power analysis-based studies the available information on the power analysis was sometimes sparse, and it was not always clear to what effect the power really related (supplemental references 102, 131, 196, 266, 267, 323, 330, 331, 337, 352, 365, 368, 370). Other studies reported a prospective power analysis with an endpoint other than infarct size (supplemental references 47, 272, 353, 361). We calculated the power for a significance level of *α* = 0.05 of the most reasonable significant “primary infarct size endpoint” and used only these data sets for a most stringent analysis, but also calculated the power all other available infarct size data sets for a more liberal analysis. When the *n*-values for a given data set were not precisely indicated but as a range, we assumed the lowest *n*-value for a conservative power estimate.

## Results

A total of 2155 papers on cardioprotection with key words “cardioprotection” or “infarct size” or “myocardial infarction”, were identified. Detailed data on search strategy and search results are summarized separately for each journal in Table 1. After exclusion of reviews, studies in humans and preclinical studies which did not compare infarct size data without and with an intervention, a total of 371 preclinical papers remained. A subset of 269 reported the methodologically most robust data using TTC, MRI, or SPECT for infarct size quantification. Among these papers with the methodologically most robust data, only 26 had used a prospective power analysis and only 17 specified infarct size as endpoint. Ten papers with a prospective study design were published in Basic Research in Cardiology, 4 in Cardiovascular Research and 12 in Circulation Research. For 40 prospectively designed data sets in small rodents (mice, rats, rabbits) the fraction of positive results was 83% and almost identical to the 89% in data sets without a prospective power calculation. Eleven of the prospectively designed data sets were in pigs, the most relevant species for potential translation to patient benefit [12]; in this small sample, 7 (64%) results were positive, notably less than the 39 out of 49 (80%) positive data sets in pig studies did not use a prospective design.

**Table 1:** Search strategy to identify publications with preclinical data on infarct size and numbers of retrieved publications

Database	Clarivate Web of Science Core Collection (https://www.webofscience.com/)
Search term	((ALL = (infarct size) OR ALL = (cardioprotection)) OR ALL = (myocardial infarction)) AND ((SO = BASIC RESEARCH IN CARDIOLOGY) OR (SO = CARDIOVASCULAR RESEARCH) OR (SO = CIRCULATION RESEARCH))
Date range	2013/01/01 to 2014/12/31

	Basic research in cardiology	Cardiovascular research	Circulation research	∑
Total records found	369	654	1132	2155
Refinement:				
• Publication type ≠ *article* or* article, early access*	77 excluded	333 excluded	714 excluded	1124
• Publications without preclinical data	154 excluded	186 excluded	320 excluded	660
Infarct size quantification by TTC, MRI, or SPECT (normalized to area at risk or LV/cardiac mass) *[endpoint for prospective power calculation: infarct size/other/not specified]*	117 *[7/1/2]*	92 *[2/0/2]*	60 *[8/2/2]*	269 [17/3/6]
Other infarct size quantification *[endpoint for prospective power calculation: infarct size / other / not specified]*	21 *[0/0/0]*	43 *[0/1/1]*	38 *[0/0/6]*	102 *[0/1/7]*
Total	138	135	98	371

When calculating retrospectively the power, combined for all three journals, and considering only data sets with the most robust infarct size quantification method, only 75% of the significant positive results had a power of  ≥ 0.9 and an additional 9% had a power of  ≥ 0.8. Thus, 16% of all significant positive results did not even reach the power threshold of 0.8. Only 13% of all analyzed data sets reported neutral results. Figure 1 depicts these results separately for Basic Research in Cardiology, Cardiovascular Research and Circulation Research. With the more liberal analyses including also infarct size data determined by histologic image analysis (Suppl Fig. 1) and additionally including all infarct size comparisons in each paper (Suppl Fig. 2), the same trend was apparent. Often, a cardioprotective effect of an intervention with low power was followed by secondary antagonist effects on this cardioprotective intervention with higher power, further supporting the notion that the cardioprotective effect was not really robust.

**Fig. 1:**
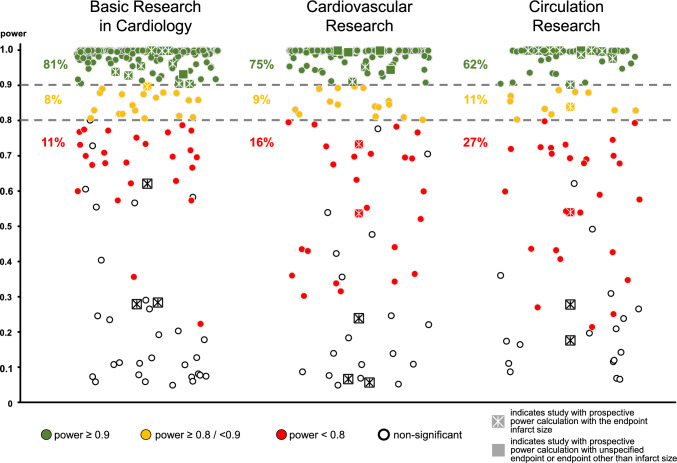
Scatterplot of the retrospectively calculated statistical power for a significance level of *α* = 0.05 of infarct size data sets which were published between 2013 and 2023 in preclinical papers in Basic Research in Cardiology, Cardiovascular Research and Circulation Research. Only infarct size measured by TTC-staining, MRI, or SPECT techniques and the most reasonable primary infarct size data sets were considered. Closed symbols indicate data with reported statistical significance, open symbols without statistical significance. Square symbols indicate data from studies with a prospective power calculation

Unfortunately, the NHLBI-sponsored CAESAR consortium [15] which had pioneered in Circulation Research 2015 the approach of a prospectively designed, multi-center trial with a core-lab centralized data analysis for experimental cardioprotection studies was not continued after the end of the funding period, but it was still followed by 17 prospective, power analysis-based study protocols in Circulation Research, and this journal in its statistical author submission guidelines continues to specifically ask for power analysis, expected effect size etc.

## Discussion

Why are preclinical studies mostly positive but the major clinical phase III trials neutral/negative? Our analysis clearly revealed that neutral results are underreported and that there is a lack of preclinical cardioprotection studies with a prospective, power analysis-based design, and a publication bias for positive results in those studies without a prospective design.

We realize that a power analysis is an established instrument to avoid a type II error in a specific, individual study, i.e., to prospectively determine a sample size with the aim to not miss an expected or reasonable effect size with a given probability. We here used a retrospective power analysis to quantitatively characterize a research field with a larger number of data sets with the effect of the size that was actually observed as sufficiently powered or not with an adequate sample size, if it were repeated. In our study, the neutral results can serve as a positive control for such use since they all had a power of less than 0.8, whether they were prospectively planned or not. However, the low power is no surprise since non-significant *p*-values always correspond to low retrospective power [13].

Thus, our use of a retrospective power analysis was not aiming to assess whether or not a given hypothesis in an individual study is indeed correct, but on the chance that positive results in a research field are repeated under the assumption of the sample size which was used and the effect size which was observed—this is in our view one essential feature of robustness [3]. Of course, there are other factors, apart from too small sample size, which undermine the robustness of preclinical cardioprotection studies, most notably lack of a priori definition of exclusion and inclusion criteria, lack of proper randomization and lack of blinding of the investigators [1, 2]. Different from power, these other factors cannot be identified retrospectively, unless they are explicitly specified in the study.

We are aware that the approach to calculating retrospective power can be criticized, since non-significant *p*-values always correspond to low retrospective power as mentioned above [13]. Nevertheless, we think that the approach is justified for our intention in the present study. We could not determine the positive predictive value because the baseline probability for a positive result was unknown for the analyzed studies.

Clinical trials are designed prospectively and must be pre-registered, e.g., on clinicaltrials.gov or else, and will not be published in a rigorous, high-impact journal unless they are pre-registered—so the final results can always be compared to the original study design, and neutral and negative data cannot be hidden. There are attempts to establish pre-registration also for preclinical studies [19] but pre-registration is unfortunately not really accepted and used by the cardioprotection community so far, and its use is not a prerequisite for publication in a decent journal—a measure that largely promoted the use of pre-registration for clinical trials.

What can be done to improve robustness of preclinical cardioprotection studies and their potential translation to clinical practice? For truly exploratory studies, a positive publication bias will certainly remain. It is unrealistic to expect scientists who have generated preliminary exploratory data which are neutral to pursue these studies and report these data; they will prefer to move on to something more exciting. However, when there is the aim of translation—and most publications in the field start in fact their introduction with an emphasis on the mortality and morbidity from ischemic heart disease—neutral data should indeed be reported. The only 13% data sets with neutral results in our analysis, therefore, most probably reflect the tip of the iceberg and contradict all reason and experience; this notion is supported by the few translationally most important pig studies with a prospective design where 36% were neutral. We realize that for innovative exploratory studies with truly novel findings, an a priori effect size cannot be estimated. However, whenever a priori information on the intervention under study exists and an effect size can be quantified or assumed for power analysis, we recommend a prospective power analysis, and—if translation to patient benefit is aimed for—a power of 0.9 for a significance level of *α* = 0.05 effect; we recognize that a power of 0.9 requires a larger effect size and/or a larger sample size. Even in the absence of prior data, when translation to patient benefit is aimed for, one could define a “clinically relevant” effect size of e.g., an infarct size reduction by 25% of the area at risk or by 5% of left ventricular mass, respectively, as a surrogate and then still use a prospective power analysis-based study design. At the very least, the authors of all studies that did not use a prospective power analysis-based study design but aim for translation could be requested to present the exact p-values and corresponding confidence intervals of their significant study results.

For journal editors, we propose to not only explicitly encourage publication of neutral data but also not to put the burden to explain discrepant results from prior studies on the authors of the neutral study, in particular when the neutral study has a power analysis-based prospective design. Strictly speaking, only the rejection of the Null hypothesis needs a reasonable explanation. Maybe, a journal editor in a case where a neutral study did not confirm a prior positive study should solicit a comment from the authors of the prior positive study. Basic Research in Cardiology has just done that, and the results were indeed enlightening [14, 16]. Also, the request for the identical repetition of a positive prior study is futile, as that would require not only use of animals of the same breed, sex and age [15–17], the same anesthesia, surgical approach and study protocol including route of administration, timing and dosing of the cardioprotective intervention, but also keeping such minute conditions constant as time of the year [16, 21], time of the day [4, 5], the composition of diet and tap water [15] which all impact on the study results and its robustness but are impossible to replicate. Of course, all these variables are also relevant for clinical cardioprotection trials and may differ between one trial and another one. Therefore, it is an important consideration whether or not a cardioprotection of interest relies on robust preclinical data before embarking on a clinical trial.

In conclusion, better reporting of positive and neutral data will further improve the rigor and robustness of preclinical cardioprotection studies and thus facilitate their translation to patient benefit [1, 2, 8, 15].

### Supplementary Information

Below is the link to the electronic supplementary material.

Supplementary file1 (PDF 1134 KB)

## References

[1] Bolli R (2021). CAESAR’s legacy: a new era of rigor in preclinical studies of cardioprotection. Basic Res Cardiol.

[2] Bolli R, Tang XL (2022). New insights into cardioprotection, gained by adopting the CAESAR standards of rigor. Basic Res Cardiol.

[3] Button KS, Ioannidis JP, Mokrysz C, Nosek BA, Flint J, Robinson ES, Munafo MR (2013). Power failure: why small sample size undermines the reliability of neuroscience. Nat Rev Neurosci.

[4] du Pre B, Van Veen T, Crnko S, Vos M, Deddens J, Doevendans P, Van Laake L (2017). Variation within variation: comparison of 24-h rhythm in rodent infarct size between ischemia reperfusion and permanent ligation. Int J Mol Sci.

[5] Durgan DJ, Pulinilkunnil T, Villegas-Montoya C, Garvey ME, Frangogiannis NG, Michael LH, Chow CW, Dyck JR, Young ME (2010). Short communication: ischemia/reperfusion tolerance is time-of-day-dependent: mediation by the cardiomyocyte circadian clock. Circ Res.

[6] Heusch G (2017). Critical issues for the translation of cardioprotection. Circ Res.

[7] Heusch G (2020). Myocardial ischaemia-reperfusion injury and cardioprotection in perspective. Nat Rev Cardiol.

[8] Heusch G (2023) Cardioprotection and its translation: a need for new paradigms? Or for new pragmatism? An opinionated retro- and perspective. J Cardiovasc Pharmacol Ther. 10.1177/10742484231179613 10.1177/10742484231179613 37259502

[9] Heusch G (2024). Myocardial ischemia/reperfusion: translational pathophysiology of ischemic heart disease. MED.

[10] Heusch G, Bøtker EH, Ferdinandy P, Schulz R (2023). Primordial non-responsiveness—a neglected obstacle to cardioprotection. Eur Heart J.

[11] Heusch G, Rassaf T (2016). Time to give up on cardioprotection? A critical appraisal of clinical studies on ischemic pre-, post-, and remote conditioning. Circ Res.

[12] Heusch G, Skyschally A, Schulz R (2011). The in-situ pig heart with regional ischemia/reperfusion—ready for translation. J Mol Cell Cardiol.

[13] Hoenig JM, Heisey DM (2001). The abuse of power: the pervasive fallacy of power calculations for data analysis. Am Stat.

[14] Ibanez B (2023). A tale of pigs, beta-blockers and genetic variants. Basic Res Cardiol.

[15] Jones SP, Tang XL, Guo Y, Steenbergen C, Lefer DJ, Kukreja RC, Kong M, Li Q, Bhushan S, Zhu X, Du J, Nong Y, Stowers HL, Kondo K, Hunt GN, Goodchild TT, Orr A, Chang CC, Ockaili R, Salloum FN, Bolli R (2015). The NHLBI-sponsored consortium for preclinicAL assESsment of cARdioprotective therapies (CAESAR): a new paradgm for rigorous, accurate, and reproducible evaluation of putative infarct-sparing interventions in mice, rabbits, and pigs. Circ Res.

[16] Kleinbongard P, Lieder HR, Skyschally A, Alloosh M, Gödecke A, Rahmann S, Sturek M, Heusch G (2022). Non-responsiveness to cardioprotection by ischaemic preconditioning in Ossabaw minipigs with genetic predisposition to, but without the phenotype of the metabolic syndrome. Basic Res Cardiol.

[17] Kleinbongard P, Lieder H, Skyschally A, Heusch G (2023). No robust reduction of infarct size and no-reflow by metoprolol pretreatment in adult Göttingen minipigs. Basic Res Cardiol.

[18] Lassen TR, Hjortbak MV, Hauerslev M, Tonnesen PT, Kristiansen SB, Jensen RV, Botker HE (2021). Influence of strain, age, origin, and anesthesia on the cardioprotective efficacy by local and remote ischemic conditioning in an ex vivo rat model. Physiol Rep.

[19] Menon JML, van der Naald M, Chamuleau SAJ, Duncker DJ (2023). Preclinicaltrials.eu: prospective registration of animal studies. Eur Heart J.

[20] Sayour NV, Brenner GB, Makkos A, Kiss B, Kovácsházi C, Gergely TG, Aukrust SG, Tian H, Zenkl V, Gömöri K, Szabados T, Bencsik P, Heinen A, Schulz R, Baxter GF, Zuurbier CJ, Vokó Z, Ferdinandy P, Giricz Z (2023). Cardioprotective efficacy of limb remote ischemic preconditioning in rats: discrepancy between meta-analysis and a three-centre in vivo study. Cardiovasc Res.

[21] Uitterdijk A, Yetgin T, Te Lintel HM, Sneep S, Krabbendam-Peters I, van Beusekom HM, Fischer TM, Cornelussen RN, Manintveld OC, Merkus D, Duncker DJ (2015). Vagal nerve stimulation started just prior to reperfusion limits infarct size and no-reflow. Basic Res Cardiol.

