# Supplementary material for: “Expression of concern”: publication bias for positive preclinical cardioprotection studies
Source: Basic Res Cardiol. 2024 Apr 26;119(3):397–402. doi: 10.1007/s00395-024-01050-4 (PMC11143004; doi:10.1007/s00395-024-01050-4)
Supplement: Supplementary file 1 — Supplementary file1 (PDF 1134 KB) [file 395_2024_1050_MOESM1_ESM.pdf]

“Expression of concern” – publication bias for positive preclinical cardioprotection studies

Andreas Skyschally[1], Petra Kleinbongard[1], Markus Neuhäuser[2], Gerd Heusch[1]

[1]Institute for Pathophysiology, West German Heart and Vascular Center, University Duisburg-Essen, Essen, Germany

[2]Department of Mathematics and Technology, Koblenz University of Applied Sciences, Rhein-Ahr-Campus, Remagen, Germany

Supplemental data

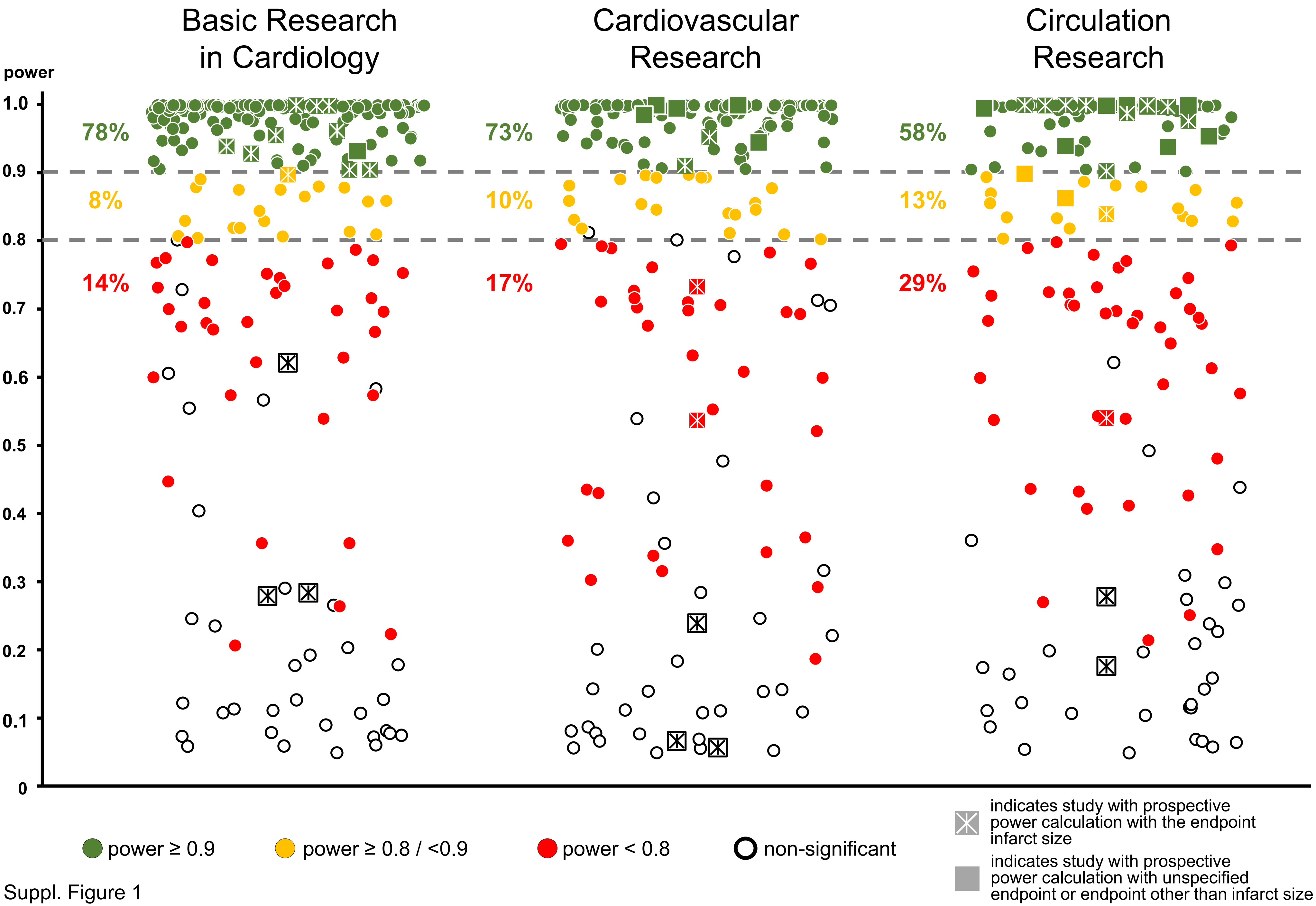

Suppl. Figure 1

Suppl. Figure 1: Scatterplot of the retrospectively calculated statistical power for a significance level of $\alpha = 0.05$ of infarct size data sets which were published between 2013 and 2023 in preclinical papers in Basic Research in Cardiology, Cardiovascular Research and Circulation Research. Only infarct size measured by TTC-staining, MRI, SPECT, or histologic techniques and the most reasonable primary infarct size data sets were considered. Closed symbols indicate data with reported statistical significance, open symbols without statistical significance. Square symbols indicate data from studies with a prospective power calculation.

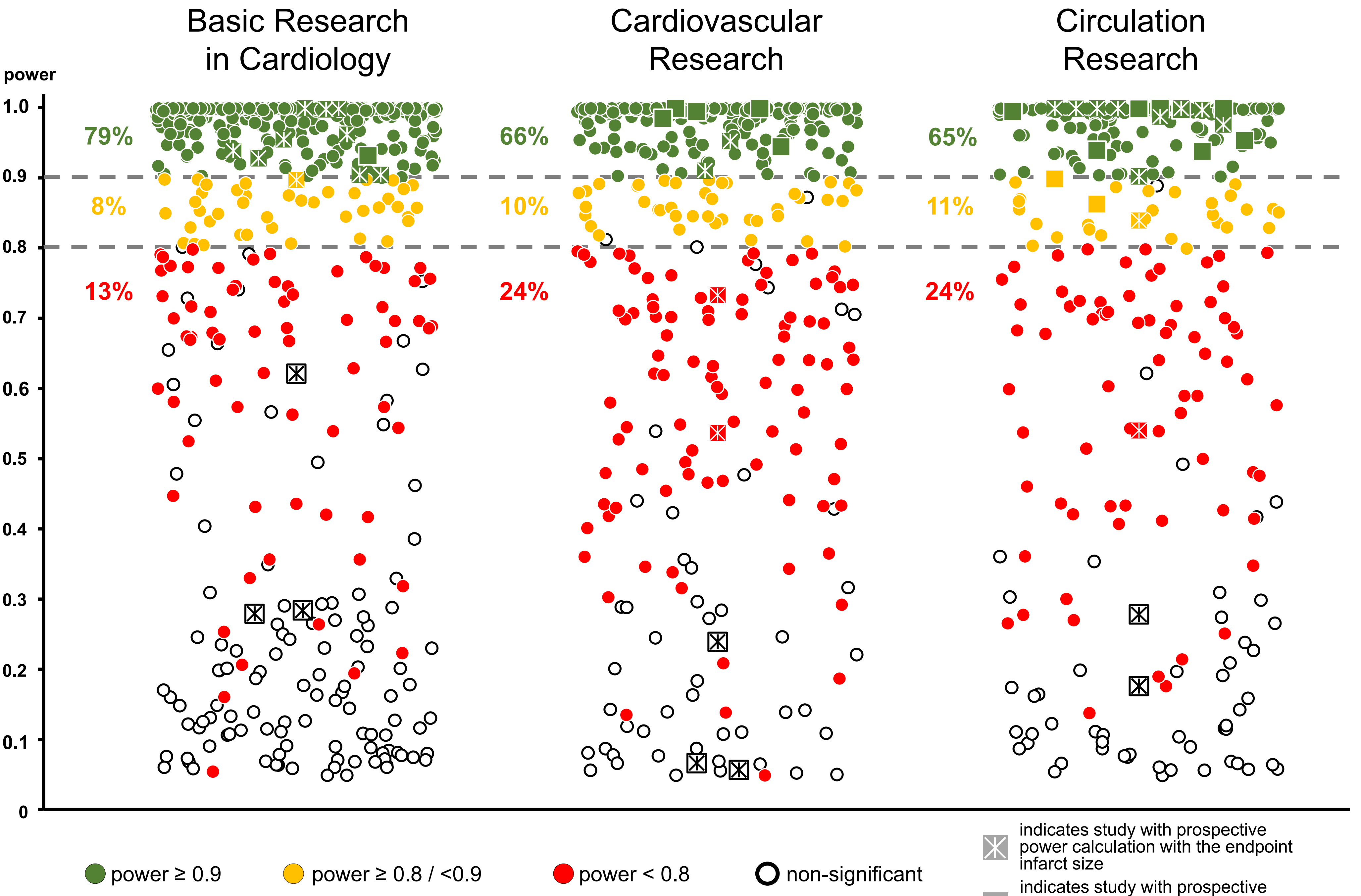

Suppl. Figure 2

Suppl. Figure 2: Scatterplot of the retrospectively calculated statistical power for a significance level of $\alpha = 0.05$ of infarct size data sets which were published between 2013 and 2023 in preclinical papers in Basic Research in Cardiology, Cardiovascular Research and Circulation Research. All available data sets on infarct size were used. Closed symbols indicate data with reported statistical significance, open symbols without statistical significance. Square symbols indicate data from studies with a prospective power calculation.

Basic Research in Cardiology								
ref.	species	I/R timing	intervention	method	effect	p-value	power	note
[1]	mouse	60 min / 24 h	WT vs CypD KO	TTC	↓	<0.01	1.00	
[1]	mouse	60 min / 24 h	WT vs NIM811	TTC	↓	<0.01	0.70	
[1]	mouse	60 min / 24 h	WT vs TUDCA	TTC	↓	<0.01	0.70	
[1]	mouse	60 min / 24 h	WT vs NIM811+TUDCA	TTC	→	n.s.	0.11	
[2]	mouse	30 min / 120 min	con vs PoCo	TTC	↓	<0.05	*	
[2]	mouse	30 min / 120 min	con vs iliomast	TTC	↓	<0.05	*	
[2]	mouse	30 min / 120 min	con vs PoCo (CyPD KO)	TTC	→	n.s.	*	
[2]	mouse	30 min / 120 min	con vs iliomast (CypD KO)	TTC	↓	<0.05	*	
[2]	mouse	30 min / 120 min	con vs iliomast	TTC	↓	<0.05	*	
[2]	mouse	30 min / 120 min	con vs iliomast (CypD KO)	TTC	↓	<0.05	*	
[3]	rabbit	30 min / 120 min	pla vs RIPC	TTC	↓	0.019	0.94	
[4]	rat	35 min / 60 min	con vs RIC	TTC	↓	<0.001	1.00	
[4]	rat	35 min / 60 min	RIC vs AMD+RIC	TTC	↑	<0.05	0.76	
[5]	rat	30 min / 120 min	con vs IPC	TTC	↓	<0.05	1.00	
[5]	rat	30 min / 120 min	con vs CsA	TTC	↓	<0.05	1.00	
[5]	rat	30 min / 120 min	con vs isoflurane	TTC	↓	<0.05	1.00	
[5]	rat	30 min / 120 min	con vs PoCo	TTC	→	n.s.	0.58	
[5]	rat	30 min / 120 min	con vs post-CsA	TTC	→	n.s.	0.61	
[5]	rat	30 min / 120 min	con vs post-isoflurane	TTC	↓	<0.05	1.00	
[6]	rat	30 min / 120 min	sedentary vs exercise	TTC	↓	<0.05	0.91	
[6]	rat	30 min / 120 min	exercise vs exercise+LNAME	TTC	↑	<0.05	0.44	
[6]	rat	30 min / 120 min	sedentary vs exercise	TTC	↓	<0.05	0.89	
[6]	rat	30 min / 120 min	exercise vs exercise+BH4	TTC	↑	<0.05	1.00	
[7]	rat	30 min / 120 min	veh vs incision	TTC	↓	<0.001	1.00	
[7]	rat	30 min / 120 min	veh vs bradykinin	TTC	↓	<0.001	1.00	
[7]	rat	30 min / 120 min	veh vs ePKC	TTC	↓	<0.001	1.00	
[7]	rat	30 min / 120 min	TAT vs TAT+incision	TTC	↓	<0.001	1.00	
[7]	rat	30 min / 120 min	veh vs ePKC	TTC	↓	<0.05	1.00	
[7]	rat	30 min / 120 min	veh+T10 vs gammaPKC	TTC	↓	<0.001	1.00	
[7]	rat	30 min / 120 min	ePKC during I vs incision after I	TTC	↑	<0.001	1.00	
[7]	rat	30 min / 120 min	incision during I vs incision after I	TTC	↑	<0.001	1.00	
[8]	mouse	40 min / 60 min	con vs GW3965	TTC	↓	<0.01	1.00	
[9]	mouse	45 min / 24 h	WT vs famotidine	TTC	↓	<0.05	0.73	
[9]	mouse	45 min / 24 h	WT vs amthamine	TTC	↑	<0.05	1.00	
[9]	mouse	45 min / 24 h	WT+amthamine vs WT+amthamine+famotidine	TTC	↓	<0.05	0.94	
[9]	mouse	45 min / 24 h	WT vs H2R KO	TTC	↓	<0.05	0.99	
[9]	mouse	perm / 24 h	WT vs famotidine	TTC	↓	<0.05	0.79	
[9]	mouse	perm / 24 h	WT vs amthamine	TTC	↑	<0.05	1.00	
[9]	mouse	perm / 24 h	WT+amthamine vs WT+amthamine+famotidine	TTC	↓	<0.05	0.93	
[9]	mouse	perm / 24 h	WT vs H2R KO	TTC	↓	<0.05	0.90	
[10]	mouse	60 min / 24 h	WT con vs WT1668	TTC	↓	<0.05	1.00	
[10]	mouse	60 min / 24 h	WT DGalN vs WT1668	TTC	↓	<0.05	1.00	
[10]	mouse	60 min / 24 h	WT H154 vs WT1668	TTC	↓	<0.05	1.00	
[10]	mouse	60 min / 24 h	WT 1612 vs WT1668	TTC	↓	<0.05	1.00	
[10]	mouse	60 min / 24 h	WT IL10ab DGalN vs WT1668	TTC	↓	<0.05	1.00	
[10]	mouse	60 min / 24 h	WT IL10ab 1668 vs WT1668	TTC	↓	<0.05	1.00	
[10]	mouse	60 min / 24 h	TLR9-D D-GalN vs WT1668	TTC	↓	<0.05	0.99	
[10]	mouse	60 min / 24 h	TLR9-D 1668 vs WT1668	TTC	↓	<0.05	0.90	
[10]	mouse	60 min / 24 h	WTcon vs WT IL10ab DGalN	TTC	↑	<0.05	1.00	
[10]	mouse	60 min / 24 h	WTcon vs WT IL10ab 1668	TTC	↑	<0.05	0.99	
[10]	mouse	60 min / 24 h	WTcon vs TLR9-D D-GalN	TTC	↓	<0.05	0.90	
[11]	mouse	30 min / 120 min	con vs PoCo	TTC	↓	<0.05	1.00	
[11]	mouse	30 min / 120 min	con vs Bay60	TTC	↓	<0.05	0.99	
[11]	mouse	30 min / 120 min	con vs MitoSNO	TTC	↓	<0.05	1.00	
[11]	mouse	30 min / 120 min	con vs Bay58	TTC	↓	<0.05	1.00	
[11]	mouse	30 min / 120 min	con vs sidenafil	TTC	↓	<0.05	1.00	
[11]	mouse	30 min / 120 min	con vs PoCo +QDO	TTC	↓	<0.05	1.00	
[11]	mouse	30 min / 120 min	con vs PoCo (PGI KO)	TTC	↓	<0.05	1.00	
[11]	mouse	30 min / 120 min	con vs Bay60 (PGI KO)	TTC	↓	<0.05	1.00	
[11]	mouse	30 min / 120 min	con vs MitoSNO (PGI KO)	TTC	↓	<0.05	1.00	
[11]	mouse	30 min / 120 min	con vs Bay58 (PGI KO)	TTC	→	n.s.	0.08	
[11]	mouse	30 min / 120 min	con vs sidenafil (PGI KO)	TTC	→	n.s.	0.26	
[11]	mouse	30 min / 120 min	con vs PoCo +QDO (PGI KO)	TTC	↓	<0.05	1.00	
[12]	mouse	30 min / 24 h	con vs Notch1 KO	TTC	↑	<0.01	0.98	
[12]	mouse	30 min / 24 h	veh vs EUK 134	TTC	↓	<0.01	0.97	
[12]	mouse	30 min / 24 h	veh vs JAGGD1	TTC	↓	<0.01	0.92	
[13]	mouse	40 min / 60 min	con vs diabetic	TTC	↑	<0.01	1.00	
[13]	mouse	40 min / 60 min	diabetic vs diabetic+insulin	TTC	↓	<0.01	0.87	
[13]	mouse	40 min / 60 min	diabetic+insulin vs diabetic+insulin+comp C	TTC	↑	<0.01	0.96	
[13]	mouse	40 min / 60 min	WT vs APN -/-	TTC	↑	<0.01	1.00	
[13]	mouse	40 min / 60 min	WT+insulin vs APN -/- +insulin	TTC	↑	<0.01	0.88	
[14]	rat	30 min / 120 min	con vs PoCo	NTB	↓	<0.01	1.00	
[14]	rat	30 min / 120 min	con vs Diazoxide	NTB	↓	<0.01	1.00	
[15]	mouse	40 min / 60 min	fl/fl+oil vs cre/fl+4-OHT	TTC	↓	<0.0001	1.00	
[15]	mouse	40 min / 60 min	fl/fl+4-OHT vs cre/fl+4-OHT	TTC	↓	<0.0001	1.00	
[15]	mouse	40 min / 60 min	cre/fl+oil vs cre/fl+4-OHT	TTC	↓	<0.0001	1.00	
[15]	mouse	40 min / 60 min	(fl/fl+oil) ctrl vs IPC	TTC	↓	<0.05	0.96	
[15]	mouse	40 min / 60 min	(fl/fl+oil) ctrl vs DZX	TTC	↓	<0.05	0.93	
[15]	mouse	40 min / 60 min	(fl/fl+4-OHT) ctrl vs IPC	TTC	↓	<0.05	0.96	
[15]	mouse	40 min / 60 min	(fl/fl+4-OHT) vs DZX	TTC	↓	<0.05	0.77	
[15]	mouse	40 min / 60 min	(cre/fl+oil ctrl) vs IPC	TTC	↓	<0.05	0.77	
[15]	mouse	40 min / 60 min	(cre/fl+oil ctrl) vs DZX	TTC	→	n.s.	0.19	
[15]	mouse	40 min / 60 min	(ctrl) fl/fl+oil vs cre/fl+4-OHT	TTC	↓	<0.001	1.00	
[15]	mouse	40 min / 60 min	(ctrl) fl/fl+4-OHT vs cre/fl+4-OHT	TTC	↓	<0.001	1.00	
[15]	mouse	40 min / 60 min	(ctrl) cre/fl+oil vs cre/fl+4-OHT	TTC	↓	<0.001	1.00	

[15]	mouse	40 min / 60 min	(IPC) fl/fl+oil vs cre/fl+4-OHT	TTC	↓	<0.001	1.00
[15]	mouse	40 min / 60 min	(IPC) fl/fl+4-OHT vs cre/fl+4-OHT	TTC	↓	<0.001	0.87
[15]	mouse	40 min / 60 min	(IPC) cre/fl+oil vs cre/fl+4-OHT	TTC	↓	<0.001	1.00
[15]	mouse	40 min / 60 min	(DZX) fl/fl+oil vs cre/fl+4-OHT	TTC	↓	<0.001	1.00
[15]	mouse	40 min / 60 min	(DZX) fl/fl+4-OHT vs cre/fl+4-OHT	TTC	↓	<0.001	0.67
[15]	mouse	40 min / 60 min	(DZX) cre/fl+oil vs cre/fl+4-OHT	TTC	↓	<0.001	1.00
[16]	mouse	30 min / 24 h	con vs ctrp9	TTC	↓	<0.01	1.00
[17]	rat	30 min / 24 h	veh vs GIK	TTC	↓	<0.05	0.99
[17]	rat	30 min / 24 h	GK vs GIK	TTC	↓	<0.05	1.00
[17]	rat	30 min / 24 h	(GIK) NDRG2 scramble vs NDRG2 KO	TTC	↑	<0.05	0.97
[18]	mouse	30 min / 120 min	con vs GAP19	TTC	↓	<0.05	0.60
[18]	mouse	30 min / 120 min	con vs GAP19i130A	TTC	→	n.s.	0.20
[19]	rat	60 min / 120 min	con vs vagal stimulation	TTC	↓	<0.01	0.98
[20]	mouse	45 min / 24 h	con vs BRL37344	TTC	↓	<0.05	0.77
[20]	mouse	45 min / 2 h	con vs BRL37344 (WT)	TTC	↓	<0.05	0.69
[20]	mouse	45 min / 2 h	con vs BRL37344 (CypD KO)	TTC	→	n.s.	0.07
[20]	mouse	45 min / 2 h	con (WT) vs BRL 37344 (CyPD KO)	TTC	↓	<0.05	0.69
[20]	pig	60 min / 7 d	con vs BRL37344	MRI	↓	<0.05	0.71
[21]	mouse	30 min / 60 min	PBS vs RIPC	TTC	↓	<0.05	0.86
[21]	mouse	30 min / 60 min	PBS vs miR-Co+RIPC	TTC	↓	<0.01	0.97
[21]	mouse	30 min / 60 min	PBS vs anti-miR144+RIPC	TTC	→	n.s.	0.20
[21]	mouse	30 min / 60 min	PBS vs anti-miR144	TTC	→	n.s.	0.09
[21]	mouse	30 min / 60 min	PBS vs miR-Co	TTC	→	n.s.	0.73
[21]	mouse	30 min / 60 min	PBS vs miR144 dy1	TTC	↓	<0.05	0.96
[21]	mouse	30 min / 60 min	PBS vs miR144 dy3	TTC	↓	<0.05	1.00
[22]	mouse	30 min / 24 h	con vs NRLP3 siRNA	TTC	↓	<0.05	0.78
[22]	mouse	30 min / 24 h	con vs BAY 117082	TTC	↓	<0.05	0.81
[23]	rat	30 min / 240 min	con vs HPC	TTC	↓	<0.05	1.00
[23]	rat	30 min / 240 min	capzasepine vs HPC+capsacepine	TTC	↓	<0.05	0.97
[23]	rat	30 min / 240 min	HPC vs HPC+capsacepine	TTC	↑	<0.05	0.20
[24]	rabbit	30 min / 120 min	(rabbit plasma dialysate) sham vs li-RIPC	TTC	↓	<0.01	0.81
[24]	rabbit	30 min / 120 min	(rabbit plasma dialysate) sham vs Tens	TTC	↓	<0.01	0.99
[24]	mouse	30 min / 60 min	(human plasma dialysate) pre li-IPC vs post-li-IPC	TTC	↓	<0.01	1.00
[24]	mouse	30 min / 60 min	(human plasma dialysate) pre TENS vs post-TENS	TTC	↓	<0.01	0.98
[24]	mouse	30 min / 60 min	(human plasma dialysate) pre TENS vs post-TENS	TTC	↓	<0.01	0.98
[24]	mouse	30 min / 60 min	(human plasma dialysate) pre TENS vs pre TENS+naloxone	TTC	↑	<0.05	0.95
[24]	mouse	30 min / 60 min	(human plasma dialysate) pre TENS+naloxone vs post-TENS+naloxone	TTC	→	n.s.	0.23
[25]	mouse	60 min / 24 h	BC+/EC+ vs BC-/EC+	TTC	↑	<0.01	0.98
[25]	mouse	60 min / 24 h	ETU- vs ETU+ (BC+/EC+)	TTC	↑	<0.001	1.00
[25]	mouse	60 min / 24 h	ETU- (BC+/EC+) vs ETU- (BC-/BC+)	TTC	↑	<0.05	1.00
[26]	mouse	perm / 3 wk	veh vs anti IL6	TTC	→	n.s.	0.08
[26]	mouse	perm / 3 wk	veh vs anti IL6	Histo	↑	<0.05	0.85
[27]	mouse	30 min / 60 min	WT vs WT+PoCo	NTB	↓	<0.01	0.98
[27]	mouse	30 min / 60 min	WT vs Mel OE	NTB	↓	<0.05	0.95
[27]	mouse	30 min / 60 min	WT vs Mel OE+PoCo	NTB	↓	<0.05	1.00
[27]	mouse	30 min / 60 min	Mel OE vs Mel OE+PoCo	NTB	→	n.s.	0.30
[27]	mouse	30 min / 60 min	Mel OE vs Mel OE+wortmannin	NTB	↑	<0.05	0.53
[27]	mouse	30 min / 60 min	Mel OE vs Mel OE+U0126	NTB	↑	<0.05	0.55
[28]	mouse	30 min / 120 min	WT vs GAB1 KO	TTC	↑	<0.05	0.89
[29]	rat	perm / 1 mo	con vs cardiospheres	Histo	↓	<0.05	0.75
[29]	rat	perm / 6 mo	con vs cardiospheres	Histo	↓	<0.05	0.16
[30]	mouse	60 min / 24 h	WT vs IFR9 KO	TTC	↓	<0.05	1.00
[30]	mouse	60 min / 24 h	WT vs IFR9 OE	TTC	↑	<0.05	0.99
[31]	mouse	perm / 1 wk	DKK3 +/+ vs DKK3 -/-	Histo	↑	<0.05	0.99
[31]	mouse	perm / 1 wk	WT vs csOE DKK3	Histo	↓	<0.05	0.99
[32]	rat	20/45 min / 120 min	i20 vs i45	TTC	↑	<0.05	0.99
[32]	rat	30 min / 120 min	con vs PoCo 10s	TTC	↓	<0.01	1.00
[32]	rat	30 min / 120 min	con vs PoCo 10min	TTC	↓	<0.05	0.99
[32]	rat	30 min / 120 min	con vs PoCo 10s	TTC	↓	<0.01	1.00
[32]	rat	30 min / 120 min	con vs PoCo 10 min	TTC	↓	<0.05	0.98
[32]	rat	30 min / 120 min	con vs PoCo 30 min	TTC	↓	<0.01	1.00
[32]	rat	30 min / 120 min	con vs PoCo 45 min	TTC	↓	<0.01	1.00
[32]	rat	30 min / 120 min	con vs PoCo 60 min	TTC	→	n.s.	0.66
[32]	rat	30 min / 120 min	con vs PoCo 10 s	TTC	↓	<0.01	1.00
[32]	rat	30 min / 120 min	con vs PoCo 10 min	TTC	↓	<0.01	1.00
[32]	rat	30 min / 120 min	PoCo 10s vs PoCo 10 s+PD	TTC	↑	<0.01	1.00
[32]	rat	30 min / 120 min	PoCo 10 s vs PoCo 10 s+LY	TTC	↑	<0.01	1.00
[32]	rat	30 min / 120 min	PoCo 10 s vs PoCo 10 s+AG	TTC	→	n.s.	0.09
[32]	rat	30 min / 120 min	PoCo 10min vs PoCo 10 min+PD	TTC	→	n.s.	0.29
[32]	rat	30 min / 120 min	PoCo 10 min vs PoCo 10 min+LY	TTC	→	n.s.	0.31
[32]	rat	30 min / 120 min	PoCo 10 min vs PoCo 10 min+AG	TTC	→	n.s.	0.15
[32]	rat	30 min / 120 min	con vs PoCo 10 s	TTC	↓	<0.01	1.00
[32]	rat	30 min / 120 min	con vs PoCo 10 min	TTC	↓	<0.01	0.99
[32]	rat	30 min / 120 min	con vs PoCo 30 min	TTC	↓	<0.01	1.00
[32]	rat	30 min / 120 min	con vs PoCo 45 min	TTC	↓	<0.01	1.00
[32]	rat	30 min / 120 min	PoCo 10 s vs PoCo 10 s+5HD	TTC	↑	<0.05	0.93
[32]	rat	30 min / 120 min	PoCo 10 min vs PoCo 10 min+5HD	TTC	↑	<0.05	0.90
[32]	rat	30 min / 120 min	PoCo 30 min vs PoCo 30 min+5HD	TTC	→	n.s.	0.13
[32]	rat	30 min / 120 min	PoCo 45 min vs PoCo 45 min+5HD	TTC	→	n.s.	0.07
[33]	mouse	perm / 3 d	WT vs p66shc KO	Histo	→	n.s.	0.18
[34]	rat	30 min / 24 h	con vs PKG Ia	TTC	↓	<0.05	1.00
[34]	rat	30 min / 24 h	PKG Ia+PAG vs PKG-Ia	TTC	↓	<0.05	1.00
[34]	rat	30 min / 24 h	K390A vs PKG-Ia	TTC	↓	<0.05	1.00
[34]	rat	30 min / 24 h	PAG vs PKG-Ia	TTC	↓	<0.05	1.00
[34]	rat	30 min / 24 h	PKG-Ia+KT5823 at reperfusion vs PKG-Ia	TTC	↓	<0.05	0.85
[34]	rat	30 min / 24 h	KT5823 at reperfusion vs PKG-Ia	TTC	↓	<0.05	1.00
[34]	rat	30 min / 24 h	con vs PKG-Ia+KT5823 at reperfusion	TTC	↓	<0.05	0.61
[34]	rat	30 min / 24 h	PKG Ia vs PKG-Ia+KT5823 at reperfusion	TTC	↑	<0.05	0.85

[34]	rat	30 min / 24 h	PKG Ia+PAG vs PKG-Ia+KT5823 at reperfusion	TTC	↓	<0.05	0.93	
[34]	rat	30 min / 24 h	K390A vs PKG-Ia+KT5823 at reperfusion	TTC	↓	<0.05	0.56	
[34]	rat	30 min / 24 h	PAG vs PKG-Ia+KT5823 at reperfusion	TTC	↓	<0.05	0.97	
[34]	rat	30 min / 24 h	KT5823 at reperfusion vs PKG-Ia+KT at reperfusion	TTC	↓	<0.05	0.94	
[35]	mouse	30 min / 60 min	C57 vs db/db DMSO	TTC	↑	<0.05	1.00	
[35]	mouse	30 min / 60 min	C57 vs db/db RAPA	TTC	↓	<0.001	1.00	
[35]	mouse	30 min / 60 min	db/db DMSO vs db/db RAPA	TTC	↓	<0.001	1.00	
[35]	mouse	30 min / 60 min	DMSO vs RAPA	TTC	↓	<0.001	1.00	
[35]	mouse	30 min / 60 min	RAPA vs DMSO(STAT3 def)	TTC	↑	<0.001	1.00	
[36]	mouse	30 min / 48 h	WT vs miR155 -/-	TTC	↓	<0.05	0.68	
[37]	rat	30 min / 90 min	con vs prolame	TTC	↓	<0.05	0.97	
[37]	rat	30 min / 90 min	con vs 17ß-estradiol	TTC	↓	<0.05	0.22	
[37]	rat	30 min / 90 min	prolame vs prolame+LNAME	TTC	↑	<0.05	1.00	
[37]	rat	30 min / 90 min	prolame vs 17ß-estradiol	TTC	↑	<0.05	0.25	
[38]	mouse	perm / 4 wk	WT vs CARD3 KO	Histo	↓	<0.05	1.00	
[38]	mouse	perm / 4 wk	WT vs CARD3 OE	Histo	↑	<0.05	1.00	
[39]	mouse	perm / 24 h	WT vs csOE MCP1	TTC	→	n.s.	0.13	
[39]	mouse	perm / 28 d	WT vs csOE MCP1	Histo	↓	<0.05	1.00	
[40]	mouse	perm / 4 wk	con vs exendin	TTC	→	n.s.	0.08	
[41]	mouse	35 min / 60 min	WT vs MLS-STAT3E	TTC	↓	<0.05	1.00	
[41]	mouse	30 min / 24 h min	WT vs MLS-STAT3E	TTC	↓	<0.05	1.00	
[42]	mouse	30 min / 60 min	euglycemia vs hyperglycemia	TTC	↑	<0.05	0.99	
[42]	mouse	30 min / 60 min	SPLX (EG vs HG)	TTC	→	n.s.	0.20	
[42]	mouse	30 min / 60 min	SPLX+SPAT (EG vs HG)	TTC	↑	<0.05	1.00	
[42]	mouse	30 min / 60 min	SPLX+SPAT EG vs SPLX+HGSPAT+EG	TTC	↑	<0.05	0.89	
[43]	mouse	45 min / 60 min	WT vs IB-MECA	TTC	↓	<0.05	0.98	
[43]	mouse	45 min / 60 min	WT vs A3AR KO	TTC	↓	<0.05	0.82	
[43]	mouse	45 min / 60 min	WT vs A3AR KO+IB-MECA	TTC	↓	<0.05	1.00	
[43]	mouse	45 min / 60 min	B6/B6 vs B6/B6+IB-MECA	TTC	↓	<0.05	1.00	
[43]	mouse	45 min / 60 min	A2A KO/B6 vs B6/B6+IB-MECA	TTC	↓	<0.05	1.00	
[43]	mouse	45 min / 60 min	A2A KO/B6*IB-MECA vs B6/B6+IB-MECA	TTC	↓	<0.05	0.99	
[44]	pig	45 min / 120 min	con vs vns	TTC	↓	<0.05	0.68	
[45]	woodchuck	60 min / 120 min	summer pla vs summer IPC	TTC	↓	<0.05	0.97	
[45]	woodchuck	60 min / 120 min	summer pla vs winter IPC (room temp)	TTC	↓	<0.05	1.00	
[45]	woodchuck	60 min / 120 min	summer pla vs winter IPC (hibernation)	TTC	↓	<0.05	1.00	
[45]	woodchuck	60 min / 120 min	summer pla vs winter+IPC	TTC	↓	<0.05	1.00	
[45]	woodchuck	60 min / 120 min	winter+L-NA vs winter (room temp)	TTC	↓	<0.05	1.00	
[45]	woodchuck	60 min / 120 min	winter+L-NA vs winter (hibernation)	TTC	↓	<0.05	1.00	
[45]	woodchuck	60 min / 120 min	winter+L-NA vs winter+IPC	TTC	↓	<0.05	1.00	
[46]	rat	30 min / 120 min	con vs cangrelor	TTC	↓	<0.001	1.00	
[46]	rat	30 min / 120 min	con vs endo III 4 mg pre	TTC	↓	<0.001	1.00	
[46]	rat	30 min / 120 min	con vs endo III+cangrelor	TTC	↓	<0.005	1.00	
[46]	rat	30 min / 120 min	cangrelor vs endo III+cangrelor	TTC	↓	<0.001	0.98	
[46]	rat	30 min / 120 min	endo III 4 mg pre vs endo III+cangrelor	TTC	↓	<0.001	0.99	
[46]	rat	30 min / 120 min	con vs endo III 4 mg at rep	TTC	↓	<0.001	0.99	
[46]	rat	30 min / 120 min	con vs endo III 8 mg at rep	TTC	↓	<0.001	1.00	
[46]	rat	30 min / 120 min	con vs endo III 8 mg at rep+cangrelor	TTC	↓	<0.001	1.00	
[46]	rat	30 min / 120 min	con vs endo III 8 mg 10 min rep	TTC	↓	<0.001	1.00	
[46]	rat	30 min / 120 min	cangrelor vs endo III 8 mg at rep+cangrelor	TTC	↓	<0.001	1.00	
[46]	rat	30 min / 120 min	endo III 4 mg rep vs endo III 8 mg rep+cangrelor	TTC	↓	<0.001	1.00	
[46]	rat	30 min / 120 min	endo III 8 mg rep vs endo III 8 mg rep+cangrelor	TTC	↓	<0.002	1.00	
[46]	rat	30 min / 120 min	con vs endo III 4 mg pre+wortmannin	TTC	↓	<0.001	1.00	
[46]	rat	30 min / 120 min	con vs endo III 4 mg pre+SPT	TTC	↓	<0.001	1.00	
[46]	rat	30 min / 120 min	endo III 4 mg pre vs endo III 4 mg pre+PD96059	TTC	↑	<0.001	1.00	
[46]	rat	30 min / 120 min	con vs DNASE 16	TTC	↓	<0.001	1.00	
[46]	rat	30 min / 120 min	con vs DNASE 32	TTC	↓	<0.001	1.00	
[46]	rat	30 min / 120 min	con vs DNASE+cangrelor	TTC	↓	<0.001	1.00	
[46]	rat	30 min / 120 min	con vs DNASE+PD	TTC	↓	<0.001	1.00	
[46]	rat	30 min / 120 min	con vs DNASE+endo III	TTC	↓	<0.001	1.00	
[46]	rat	30 min / 120 min	DNASE+cangrelor vs cangrelor	TTC	↑	0.006	0.98	
[46]	rat	30 min / 120 min	DNASE+cangrelor vs DNASE16	TTC	↑	<0.02	0.77	
[46]	rat	30 min / 120 min	DNASE+cangrelor vs DNASE32	TTC	↑	<0.02	0.98	
[46]	rat	30 min / 120 min	DNASE+endo III vs DNASE16	TTC	↑	<0.01	0.81	
[46]	rat	30 min / 120 min	DNASE+endo III vs DNASE32	TTC	↑	<0.01	0.98	
[46]	rat	30 min / 120 min	DNASE+endo III vs Endo III	TTC	↑	<0.01	0.99	
[46]	rat	30 min / 120 min	con vs DNASE333	TTC	↓	<0.001	1.00	
[46]	rat	30 min / 120 min	con vs mtDNA	TTC	↑	<0.001	1.00	
[47]	pig	60 min / 180 min	con vs CsA	TTC	↓	0.008	0.99	①
[47]	pig	60 min / 180 min	con vs PoCo	TTC	↓	0.016	0.92	①
[48]	pig	90 min / 21 d	normal cholesterol diet vs high cholesterol diet	TTC	↑	<0.05	0.57	
[48]	mouse	60 min / 0 min	WT vs LRP5-/-	Histo	↑	<0.05	0.67	
[48]	mouse	60 min / 0 min	WT vs LRP5-/-	Histo	↑	<0.05	0.42	
[49]	dog	60 min / 600 min	non.hypoxic vs intermittent hypoxia	TTC	↓	<0.001	1.00	
[49]	dog	60 min / 600 min	intermittent hypoxia vs intermittend hypoxia+naltrindole	TTC	↑	<0.001	1.00	
[49]	dog	60 min / 600 min	intermittent hypoxia vs intermittend hypoxia+NAC	TTC	↑	<0.001	1.00	
[49]	dog	60 min / 600 min	intermittent hypoxia vs uniterrupted hypoxia	TTC	↑	<0.001	1.00	
[50]	mouse	45 min / 24 h	con vs DMS	TTC	↓	<0.05	0.88	
[50]	mouse	45 min / 24 h	con vs DMS+PC61	TTC	→	n.s.	0.20	
[50]	mouse	45 min / 24 h	con vs DMS+IgG	TTC	↓	<0.05	0.87	
[50]	mouse	45 min / 24 h	con vs DMS+LY	TTC	→	n.s.	0.17	
[50]	mouse	45 min / 24 h	WT vs RAG2 KO	TTC	↓	<0.05	0.88	
[50]	mouse	45 min / 24 h	WT vs RAG2 KO+DMS	TTC	↓	<0.05	0.95	
[50]	mouse	45 min / 24 h	WT vs RAG2 KO+DMS+nonTREGS (wt)	TTC	→	n.s.	0.08	
[50]	mouse	45 min / 24 h	WT vs RAG2 KO+DMS+nonTREGS (wt) +TREGS (wt)	TTC	↓	<0.05	0.79	
[50]	mouse	45 min / 24 h	WT vs RAG2 KO+DMS+TREGS (WT)	TTC	↓	<0.05	0.94	
[50]	mouse	45 min / 24 h	WT vs RAG2 KO +DMS+nonTREGS (wt) +TREGS (AKT1+/-)	TTC	→	n.s.	0.06	
[51]	mouse	45 min / 24 h	WT vs Caspase KO	TTC	→	n.s.	0.13	
[52]	mouse	25 min / 60 min	con vs RIPC 2 cyc	TTC	→	n.s.	0.13	

[52]	mouse	25 min / 60 min	con vs RIPC 4 cyc	TTC	↓	<0.05	0.94
[52]	mouse	25 min / 60 min	con vs RIPC 6 cyc	TTC	↓	<0.001	1.00
[52]	mouse	25 min / 60 min	con vs RIPC 8 cyc	TTC	↓	<0.05	1.00
[52]	mouse	25 min / 60 min	4 cy vs RIPC 6 cyc	TTC	→	n.s.	0.27
[52]	mouse	25 min / 60 min	RIPC 6 cyc vs RIPC 8 cyc	TTC	→	n.s.	0.20
[52]	mouse	25 min / 60 min	con vs RIPC 2min occ	TTC	↓	<0.05	0.97
[52]	mouse	25 min / 60 min	con vs RIPC 5 min occ	TTC	↓	<0.05	1.00
[52]	mouse	25 min / 60 min	con vs RIPC 10 min occ	TTC	→	n.s.	0.09
[52]	mouse	25 min / 60 min	RIPC 2min occ vs RIPC 5min occ	TTC	→	n.s.	0.08
[52]	mouse	25 min / 60 min	con vs RIPC 4cyc-1limb	TTC	↓	<0.001	0.99
[52]	mouse	25 min / 60 min	con vs RIPC 4 cyc-2limb	TTC	↓	<0.05	0.95
[52]	mouse	25 min / 60 min	RIPC 4 cyc-1limb vs RIPC 4 cyc-2limb	TTC	→	n.s.	0.07
[52]	mouse	25 min / 60 min	con vs RIPC 6cyc-1limb	TTC	↓	<0.001	1.00
[52]	mouse	25 min / 60 min	con vs RIPC 6cyc-2limb	TTC	↓	<0.0001	1.00
[52]	mouse	25 min / 60 min	RIPC 6cyc-1limb vs RIPC 6cyc-2limb	TTC	→	n.s.	0.25
[52]	mouse	25 min / 60 min	con vs RIPC 0.5h delay to isch	TTC	↓	<0.001	1.00
[52]	mouse	25 min / 60 min	con vs RIPC 1.5h delay to isch	TTC	↓	<0.0001	1.00
[52]	mouse	25 min / 60 min	con vs RIPC 2h delay to isch	TTC	→	n.s.	0.16
[52]	mouse	25 min / 60 min	con vs RIPC 2.5h delay to isch	TTC	→	n.s.	0.11
[52]	mouse	25 min / 60 min	con vs IPC	TTC	↓	<0.0001	1.00
[53]	mouse	40 min / 85 min	con vs HFS (hig-Fat-Sucrose)	TTC	↑	<0.05	0.97
[53]	mouse	40 min / 85 min	HFS vs HFS+exercise	TTC	↓	<0.05	0.75
[53]	mouse	40 min / 120 min	con vs HFS (hig-Fat-Sucrose)	TTC	↑	<0.05	0.67
[53]	mouse	40 min / 120 min	HFS vs HFS+exercise	TTC	↓	<0.05	0.67
[53]	mouse	40 min / 120 min	con vs BRL37344 (ß3-stim)	TTC	↓	<0.05	0.92
[53]	mouse	40 min / 120 min	con vs HFS	TTC	↑	<0.05	1.00
[53]	mouse	40 min / 120 min	HFS vs HFS+BRL37344	TTC	→	n.s.	0.12
[53]	mouse	40 min / 85 min	HFS vs HFS+1400W (iNOS inh)	TTC	↓	<0.05	0.93
[54]	mouse	perm / 7 d	saline vs ROFA (1 mm)	Histo	→	n.s.	0.48
[54]	mouse	perm / 7 d	saline vs ROFA (1.5 mm	Histo	→	n.s.	0.29
[54]	mouse	perm / 7 d	saline vs ROFA (2 mm)	Histo	↑	<0.05	1.00
[54]	mouse	perm / 7 d	saline vs ROFA (2.5 mm)	Histo	→	n.s.	0.66
[54]	mouse	perm / 7 d	saline vs ROFA (3 mm)	Histo	↑	<0.05	1.00
[55]	rat	35 min / 60 min	con donor hearts vs RIC donor hearts	TTC	↓	<0.01	0.97
[55]	rat	35 min / 60 min	prp-dialysate (con) vs prp-dialysate (RIC)	TTC	↓	<0.01	0.99
[55]	rat	35 min / 60 min	ppp-dialysate (con) vs ppp-dialysate (RIC)	TTC	↓	<0.001	1.00
[55]	rat	35 min / 60 min	sham vagotomy con dialysate vs sham vagotomy+RIC dialysate	TTC	↓	<0.05	1.00
[55]	rat	35 min / 60 min	sham vagotomy+con dialysate vs vagotomy+con dialysate	TTC	→	n.s.	0.57
[55]	rat	35 min / 60 min	sham vagotomy+con dialysate vs vagotomy+RIC dialysate	TTC	→	n.s.	0.07
[55]	rat	35 min / 60 min	con (dialysate) vs RIC (dialysate)	TTC	↓	<0.05	1.00
[55]	rat	35 min / 60 min	con (dialysate) vs hexamethonium	TTC	→	n.s.	0.06
[55]	rat	35 min / 60 min	con (dialysate) vs RIC+hexamethonium	TTC	→	n.s.	0.39
[55]	rat	35 min / 60 min	con (dialysate) vs atropine	TTC	→	n.s.	0.16
[55]	rat	35 min / 60 min	con (dialysate) vs RIC+atropine	TTC	→	n.s.	0.63
[56]	mouse	30 min / 180 min	con vs solid lipid nanoparticles	TTC	↓	<0.001	1.00
[57]	mouse	40 min / 60 min	con vs SPLX (40 min I)	TTC	↓	<0.05	1.00
[57]	mouse	50 min / 60 min	con vs SPLX (50 min I)	TTC	↓	<0.05	1.00
[57]	mouse	40 min / 60 min	con vs SPLX (40 min I)	TTC	↓	<0.05	1.00
[57]	mouse	40 min / 60 min	SPLX vs SPLX+SPAT	TTC	↑	<0.05	1.00
[57]	mouse	40 min / 60 min	con vs SPLX+SPAT	TTC	→	n.s.	0.16
[57]	mouse	20 min / 60 min	SPLX vs SPLX+SPAT	TTC	→	n.s.	0.06
[57]	mouse	20 min / 60 min	con vs 10-IHH	TTC	→	n.s.	0.20
[57]	mouse	20 min / 60 min	con vs 40-IHH	TTC	↑	<0.05	1.00
[57]	mouse	20 min / 60 min	10-IHH vs 40-IHH	TTC	↑	<0.05	1.00
[57]	mouse	20 min / 60 min	40-IHH vs 40-IHH+SPLX	TTC	↓	<0.05	1.00
[57]	mouse	20 min / 60 min	40-IHH+SPLX vs 40-IHH+SPLX+SPAT	TTC	↑	<0.05	1.00
[57]	mouse	20 min / 60 min	40-IHH (H mgB1- vs H mgB1+)	TTC	↑	<0.05	1.00
[58]	mouse	60 min / 24 h	WT vs SIRT6 +/-	TTC	↑	<0.05	0.97
[58]	mouse	45 min / 60 min	WT vs SIRT6 +/-	TTC	↑	<0.001	1.00
[58]	mouse	60 min / 24 h	WT+Ad-null vs SIRT6+/- +Ad-null	TTC	↑	<0.001	1.00
[58]	mouse	60 min / 24 h	WT+Ad-null vs SIRT6+/- +Ad-SIRT6	TTC	↓	<0.001	1.00
[58]	mouse	60 min / 24 h	SIRT6+/- +Ad-null vs SIRT6+/- +Ad-SIRT6	TTC	↓	<0.001	1.00
[59]	mouse	30 min / 24 h	PBS vs mouse EVs baseline	TTC	↓	<0.05	1.00
[59]	mouse	30 min / 24 h	PBS vs mouse EVs after swimming	TTC	↓	<0.05	1.00
[59]	mouse	30 min / 24 h	PBS vs hum. plasma EVs exercise	TTC	↓	<0.05	1.00
[60]	mouse	perm / 24 h	WT vs GLO1 OE	TTC	→	n.s.	0.18
[60]	mouse	perm / 4 wk	WT vs GLO1 OE	Histo	↓	0.038	0.75
[61]	mouse	perm / 21 d	young vs old (empty-v)	Histo	↑	<0.001	0.95
[61]	mouse	perm / 21 d	old (empty V) vs old+GDF11	Histo	↓	<0.05	0.54
[62]	mouse	30 min / 120 min	con vs GAP26	TTC	↓	<0.05	0.67
[62]	mouse	30 min / 120 min	con vs GAP19	TTC	↓	<0.05	0.62
[62]	mouse	30 min / 120 min	con vs RRNY	TTC	↓	<0.01	0.99
[62]	mouse	30 min / 120 min	GAP19 vs RRNY	TTC	↓	<0.05	0.99
[63]	rat	30 min / 60 min	con vs PoCo	TTC	↓	<0.05	1.00
[63]	rat	30 min / 60 min	PoCo vs PoCo+methyl-ß cyclodextrin	TTC	↑	<0.05	0.58
[64]	pig	45 min / 1 wk	con vs 12 min	MRI	→	n.s.	0.29
[64]	pig	45 min / 1 wk	con vs 17 min	MRI	→	n.s.	0.09
[65]	mouse	45 min / 24 h	con vs AZP-531	TTC	↓	<0.001	0.98
[65]	mouse	45 min / 24 h	con vs CsA	TTC	↓	<0.01	0.70
[65]	mouse	45 min / 24 h	con vs UAG	TTC	↓	<0.001	1.00
[66]	mouse	8 h / 0	MI vs MI+antagomiR143	TTC	↓	<0.01	1.00
[66]	mouse	8 h / 0	MI vs MI+neg. con	TTC	→	n.s.	0.19
[67]	mouse	30 min / 24 h	con vs DCCI (1d)	TTC	↓	<0.05	0.92
[67]	mouse	30 min / 7 d	con vs DCCI (7d)	Histo	↓	<0.05	0.92
[68]	rat	perm / 3 d	MI vs MI+iVPCs (endo)	Histo	↓	<0.05	0.45
[68]	rat	perm / 3 d	MI vs MI+iVPCs+PLGA (endo)	Histo	↓	<0.05	0.42
[68]	rat	perm / 3 d	MI vs MI+iVPCs (epi)	Histo	↓	<0.05	0.36
[68]	rat	perm / 3 d	MI vs MI+iVPCs+PLGA (epi)	Histo	↓	<0.05	0.32

[69]	mouse	perm / 7 d	con vs IL10	TTC	→	n.s.	0.08
[70]	mouse	48 h / 28 d	WT vs iNOS -/-	TTC	→	n.s.	0.06
[71]	rat	30 min / 120 min	con vs paroxetine	TTC	→	n.s.	0.27
[72]	rat	30 min / 60 min	con vs 0.25x	TTC	→	n.s.	0.07
[72]	rat	30 min / 60 min	con vs 0.5x	TTC	→	n.s.	0.74
[72]	rat	30 min / 60 min	con vs 0.75x	TTC	↓	<0.001	1.00
[72]	rat	30 min / 60 min	con vs 1x	TTC	↓	<0.001	1.00
[72]	rat	30 min / 60 min	con vs 2x	TTC	↓	<0.001	1.00
[72]	rat	30 min / 60 min	con vs IPC	TTC	↓	<0.001	1.00
[72]	rat	30 min / 60 min	con vs <3 kDa	TTC	↓	<0.001	1.00
[72]	rat	30 min / 60 min	con vs 3-5 kDa	TTC	→	n.s.	0.67
[72]	rat	30 min / 60 min	con vs 5-10 kDa	TTC	↓	<0.001	1.00
[72]	rat	30 min / 60 min	con vs <10 kDa	TTC	↓	<0.001	1.00
[72]	rat	30 min / 60 min	con vs 10-30 kDa	TTC	→	n.s.	0.75
[72]	rat	30 min / 60 min	con vs 30-50 kDa	TTC	→	n.s.	0.23
[72]	rat	30 min / 60 min	con vs >50 kDa	TTC	→	n.s.	0.12
[72]	rat	30 min / 60 min	5-10 vs 5-10+chelerytrine	TTC	↑	<0.001	1.00
[72]	rat	30 min / 60 min	5-10 vs 5-10+glibenclamide	TTC	↑	<0.001	1.00
[72]	rat	30 min / 60 min	5-10 vs 5-10+5-HD	TTC	↑	<0.001	1.00
[72]	rat	30 min / 60 min	con vs 5-10 kDa	TTC	↓	<0.001	1.00
[72]	rat	30 min / 60 min	con vs 5-10 Hphob	TTC	↓	<0.001	1.00
[72]	rat	30 min / 60 min	con vs 5-10 Hphil	TTC	↓	<0.05	0.36
[72]	rat	30 min / 60 min	con vs 5-10 kDa	TTC	↓	<0.001	1.00
[72]	rat	30 min / 60 min	con vs HSP10	TTC	↓	<0.001	1.00
[72]	rat	30 min / 60 min	con vs 5-10+PU-H71	TTC	↓	<0.05	0.97
[72]	rat	30 min / 60 min	HSP10 vs HSP10+PU-H-71	TTC	↑	<0.001	0.97
[72]	rat	30 min / 60 min	5-10 vs 5-10+PU-H71	TTC	↑	<0.01	1.00
[73]	rat	35 min / 60 min	con vs hexamethonium	TTC	→	n.s.	0.25
[73]	rat	35 min / 60 min	con vs IPC	TTC	↓	<0.05	1.00
[73]	rat	35 min / 60 min	con vs IPC +hex	TTC	↓	<0.05	0.43
[73]	rat	35 min / 60 min	IPC vs IPC +hex	TTC	↑	<0.05	0.91
[73]	rat	35 min / 60 min	con vs IPC	TTC	↓	<0.05	1.00
[73]	rat	35 min / 60 min	con vs IPC +atropine	TTC	→	n.s.	0.79
[73]	rat	35 min / 60 min	IPC vs IPC +atropine	TTC	↑	<0.05	1.00
[73]	rat	35 min / 60 min	con vs IPC (effluent)	TTC	↓	<0.05	0.82
[73]	rat	35 min / 60 min	IPC (eff) vs IPC (eff)+hex	TTC	↑	<0.05	0.94
[74]	pig	40 min / 240 min	pla vs coversin	TTC	↓	0.03	0.83
[75]	mouse	perm / 2 wk	WT vs Sema3A KO	Histo	→	n.s.	0.12
[76]	mouse	40 min / 120 min	con vs 1x IPC	TTC	→	n.s.	0.17
[76]	mouse	40 min / 120 min	con vs 4x IPC	TTC	↓	<0.01	0.99
[76]	mouse	40 min / 120 min	con vs IPC (PI3kK inh. dur. prot.)	TTC	↓	<0.001	1.00
[76]	mouse	40 min / 120 min	con vs IPC +G326 (PI3kK inh. dur. prot.)	TTC	↓	<0.001	1.00
[76]	mouse	40 min / 120 min	con vs IPC +BYL719 (PI3kK inh. dur. prot.)	TTC	↓	<0.001	0.99
[76]	mouse	40 min / 120 min	con vs IPC (PI3kK inh. dur. rep.)	TTC	↓	<0.001	1.00
[76]	mouse	40 min / 120 min	con vs IPC +G326 (PI3kK inh. dur. rep.)	TTC	→	n.s.	0.14
[76]	mouse	40 min / 120 min	con vs IPC +BYL719 (PI3kK inh. dur. rep.)	TTC	→	n.s.	0.12
[76]	mouse	40 min / 120 min	con vs IPC (PI3kK inh. dur. rep.)	TTC	↓	<0.001	0.95
[76]	mouse	40 min / 120 min	con vs IPC +G326 (PI3kK inh. dur. rep.)	TTC	→	n.s.	0.11
[76]	mouse	40 min / 120 min	con vs bradykinin	TTC	↓	<0.001	1.00
[76]	mouse	40 min / 120 min	con vs insulin	TTC	↓	<0.001	1.00
[76]	mouse	40 min / 120 min	con vs insulin+G326	TTC	→	n.s.	0.35
[77]	rat	20 min / 120 min	sham vs nephrectomy	TTC	↑	<0.05	0.86
[78]	pig	120 min / 3 d	con vs intermittent pacing	MRI	→	n.s.	0.05
[78]	pig	120 min / 37 d	con vs intermittent pacing	MRI	→	n.s.	0.40
[79]	mouse	45 min / 24 h	WT vs Suv39h1 -/-	TTC	↓	<0.01	0.95
[79]	mouse	45 min / 24 h	WT vs Suv39h2 -/-	TTC	→	n.s.	0.24
[79]	mouse	45 min / 24 h	veh vs chaetocin	TTC	↓	<0.001	1.00
[80]	rat	60 min / 120 min	veh vs ticagrelor	TTC	↓	0.01999	0.92
[80]	rat	60 min / 120 min	veh vs VX-765	TTC	↓	0.01999	1.00
[80]	rat	60 min / 120 min	veh vs IPOC	TTC	↓	0.01999	0.92
[80]	rat	60 min / 120 min	veh vs cangrelor+VX-765	TTC	↓	0.01999	1.00
[80]	rat	60 min / 120 min	veh vs ticagrelor+VX-765	TTC	↓	0.01999	1.00
[80]	rat	60 min / 120 min	veh vs ticagrelor+IPOC	TTC	↓	0.01999	0.84
[80]	rat	60 min / 120 min	ticagrelor vs cangrelor+VX-765	TTC	↓	<0.001	1.00
[80]	rat	60 min / 120 min	ticagrelor vs ticagrelor+VX-765	TTC	↓	<0.001	1.00
[80]	rat	60 min / 120 min	ticagrelor vs ticagrelor+IPOC	TTC	↑	<0.001	0.06
[80]	rat	60 min / 3 d	chronic veh vs chronic cangrelor+VX-765	TTC	↓	<0.001	1.00
[80]	rat	40 min / 120 min	veh vs VX-765	TTC	↓	<0.001	1.00
[80]	rat	40 min / 90 min	veh vs VRT-043198	TTC	↓	<0.001	1.00
[81]	pig	60 min / 180 min	con vs IPC	TTC	↓	<0.05	1.00
[81]	pig	60 min / 180 min	con vs PoCo	TTC	↓	<0.05	1.00
[81]	pig	60 min / 180 min	con vs RIPC	TTC	↓	<0.05	1.00
[82]	mouse	60 min / 120 min	con vs IPC	TTC	↓	<0.001	1.00
[82]	mouse	60 min / 120 min	con vs VASP-/-	TTC	↓	<0.001	1.00
[82]	mouse	60 min / 120 min	con vs IPC+VASP -/-	TTC	↓	<0.001	1.00
[82]	mouse	60 min / 120 min	VASP -/- vs IPC	TTC	↓	<0.001	1.00
[82]	mouse	60 min / 120 min	IPC vs IPc+VASP -/-	TTC	↑	<0.001	1.00
[82]	mouse	60 min / 120 min	IPC vs IPC+VASP KO	TTC	→	n.s.	0.50
[83]	pig	60 min / 120 min	con vs LC-VNS	TTC	↓	<0.05	1.00
[83]	pig	60 min / 120 min	con vs LC-VNS+LtVNX	TTC	↓	<0.05	1.00
[83]	pig	60 min / 120 min	con vs LC-VNS+RtVNX	TTC	↓	<0.05	1.00
[83]	pig	60 min / 120 min	con vs LC-VNS+atropine	TTC	→	n.s.	0.27
[83]	pig	60 min / 120 min	LC-VNS+LC-VNS vs LC-VNS+RtVNX	TTC	↑	<0.05	1.00
[83]	pig	60 min / 120 min	LC-VNS+LtVNX vs LC-VNS+RtVNX	TTC	↑	<0.05	1.00
[83]	pig	60 min / 120 min	LC-VNS vs atropine	TTC	↑	<0.05	1.00
[83]	pig	60 min / 120 min	LtVNX vs atropine	TTC	↑	<0.05	1.00
[83]	pig	60 min / 120 min	RtVNX vs atropine	TTC	↑	<0.05	1.00
[84]	rat	45 min / 120 min	lean con vs lean RIPC	TTC	↓	<0.01	1.00

[84]	rat	45 min / 120 min	lean con vs fatty con	TTC	→	n.s.	0.06	
[84]	rat	45 min / 120 min	lean con vs fatty RIPC	TTC	→	n.s.	0.11	
[85]	mouse	40 min / 120 min	con vs GNTN patch (before isch)	TTC	↓	<0.001	1.00	
[85]	mouse	40 min / 120 min	con vs RIPC	TTC	↓	<0.001	0.98	
[85]	mouse	40 min / 120 min	con vs GNTN patch (before rep)	TTC	↓	<0.01	1.00	
[86]	mouse	30 min / 24 h	WT vs Spry1 cKO	TTC	↓	<0.05	0.83	
[86]	mouse	30 min / 24 h	Spry fl/fl vs Spry1 cKO	TTC	↓	<0.01	0.99	
[87]	pig	60 min / 180 min	pla vs IPC	TTC	↓	<0.05	1.00	
[87]	pig	60 min / 180 min	pla vs RIPC	TTC	↓	<0.05	1.00	
[87]	pig	60 min / 180 min	pla vs RPER	TTC	↓	<0.05	1.00	
[87]	pig	60 min / 180 min	pla vs PoCo	TTC	↓	<0.05	1.00	
[88]	pig	60 min / 120 min	con vs canagliflozin	TTC	↓	<0.05	0.81	
[89]	rat	30 min / 24 h	con vs RIPC	TTC	↓	<0.05	0.94	
[89]	rat	30 min / 24 h	con vs ED5 SCR-RIPC	TTC	↓	<0.05	1.00	
[89]	rat	30 min / 24 h	RIPC vs ED5-RIPC	TTC	↑	<0.0001	1.00	
[89]	rat	30 min / 24 h	ED5-RIPC vs ED5 SCR-RIPC	TTC	↓	<0.0001	1.00	
[90]	mouse	30 min / 24 h	WT-contol vs WT-linagliptin	TTC	↓	<0.001	1.00	
[90]	mouse	30 min / 24 h	WT-contol vs WT-exendin-4	TTC	↓	<0.001	0.99	
[90]	mouse	30 min / 24 h	db/db-contol vs db/db-linagliptin	TTC	↓	<0.001	1.00	
[90]	mouse	30 min / 24 h	db/db-contol vs db/db-exendin	TTC	↓	<0.001	1.00	
[91]	mouse	30 min / 24 h	(L5) WT/WT vs OE/OE+DOX	TTC	→	n.s.	0.15	
[91]	mouse	30 min / 24 h	(L5) WT/WT vs WT/WT+NS398	TTC	→	n.s.	0.22	
[91]	mouse	30 min / 24 h	(L5) WT/WT vs WT/OE	TTC	→	n.s.	0.16	
[91]	mouse	30 min / 24 h	(L5) WT/WT vs OE/OE	TTC	↓	<0.05	0.88	
[91]	mouse	30 min / 24 h	(L5) WT/WT vs OE/OE+NS398	TTC	→	n.s.	0.06	
[91]	mouse	30 min / 24 h	(L7) WT/WT vs WT/OE	TTC	→	n.s.	0.15	
[91]	mouse	30 min / 24 h	(L7) WT/WT vs OE/OE	TTC	↓	<0.05	1.00	
[91]	mouse	30 min / 24 h	(L8) WT/WT vs WT/OE	TTC	→	n.s.	0.13	
[91]	mouse	30 min / 24 h	(L8) WT/WT vs OE/OE	TTC	↓	<0.05	1.00	
[92]	rabbit	30 min / 180 min	con vs discrete hypothermia	TTC	↓	<0.05	1.00	
[92]	rabbit	30 min / 180 min	con vs continuous hypothermia	TTC	↓	<0.05	1.00	
[92]	rabbit	30 min / 180 min	discrete hypothermia vs continuous hypothermia	TTC	↓	<0.05	1.00	
[92]	rabbit	30 min / 180 min	con vs malonate	TTC	↓	<0.05	0.98	
[92]	rabbit	30 min / 180 min	malonate vs malonate+discrete hypothermia	TTC	↓	<0.05	1.00	
[92]	mouse	30 min / 120 min	normotherm vs hypotherm	TTC	↓	<0.05	1.00	
[93]	rat	30 min / 180 min	veh vs GDF11	TTC	↓	<0.05	1.00	
[93]	rat	30 min / 180 min	GDF11 vs FST+GDF11	TTC	↑	<0.05	0.99	
[93]	rat	30 min / 180 min	GDF11 vs FST	TTC	↑	<0.05	1.00	
[94]	rat	40 min / 28 d	28d vs 28d+INFa	Histo	↑	0.002	0.91	
[94]	rat	40 min / 4 d	4d vs 4d+INFa	Histo	→	n.s.	0.28	
[95]	mouse	25 min / 40 min	con vs E2 (male)	TTC	↓	<0.05	0.77	
[95]	mouse	25 min / 40 min	con vs E2 (OVX-female)	TTC	↓	<0.05	0.88	
[95]	mouse	25 min / 40 min	male vs fem	TTC	↓	<0.05	0.85	
[96]	rat	30 min / 120 min	con vs H2O2-Poc	TTC	↓	<0.001	1.00	
[96]	rat	30 min / 120 min	con vs Ctrl+AG490	TTC	→	n.s.	0.07	
[96]	rat	30 min / 120 min	con H2O2-Poc+AG490	TTC	→	n.s.	0.07	
[96]	rat	30 min / 120 min	H2O2-POC vs H2O2-POC+AG490	TTC	↑	<0.001	1.00	
[97]	rat	40 min / 120 min	con vs RIC	TTC	↓	<0.05	0.99	
[97]	rat	40 min / 120 min	RIC vs 1MT	TTC	↑	<0.05	0.97	
[97]	rat	40 min / 120 min	RIC vs RIC+1MT	TTC	↑	<0.05	0.87	
[98]	pig	90 min / 24 h	con vs DFP	MRI	→	n.s.	0.11	
[98]	pig	90 min / 1 wk	con vs DFP	MRI	→	n.s.	0.25	
[98]	pig	90 min / 28 d	con vs DFP	MRI	→	n.s.	0.07	
[99]	rat	30 min / 120 min	Veh vs RIC	TTC	↓	<0.001	0.99	
[99]	rat	30 min / 120 min	Veh vs emricasan	TTC	↓	<0.001	0.98	
[99]	rat	30 min / 120 min	Veh vs background drugs	TTC	↓	<0.001	0.95	
[99]	rat	30 min / 120 min	Veh vs background drugs+RIC	TTC	↓	<0.01	0.97	
[99]	rat	30 min / 120 min	Veh vs background drugs+emricasan	TTC	↓	<0.001	1.00	
[99]	rat	30 min / 120 min	background drugs vs background drugs+emricasan	TTC	↓	<0.01	0.86	
[99]	rat	30 min / 120 min	Veh vs background drugs	TTC	↓	<0.001	1.00	
[99]	rat	30 min / 120 min	Veh vs background drugs+wortmannin	TTC	→	n.s.	0.31	
[99]	rat	30 min / 120 min	Veh vs emricasan	TTC	↓	<0.01	0.99	
[99]	rat	30 min / 120 min	Veh vs emricasan+wortmannin	TTC	↓	<0.01	0.99	
[99]	rat	30 min / 120 min	background drugs vs background drugs+wortmannin	TTC	↑	<0.01	0.96	
[100]	mouse	60 min / min	con vs NEU1 OE	Histo	→	n.s.	0.09	
[101]	mouse	perm / 1 wk	con vs M0-Exo	TTC	→	n.s.	0.11	
[102]	pig	30 min / 1 wk	con vs metoprolol	MRI	↓	<0.05	0.79	②
[102]	pig	35 min / 1 wk	con vs metoprolol	MRI	↓	<0.05	0.93	②
[102]	pig	40 min / 1 wk	con vs metoprolol	MRI	↓	<0.01	0.98	②
[102]	pig	45 min / 1 wk	con vs metoprolol	MRI	↓	<0.01	0.90	②
[102]	pig	50 min / 1 wk	con vs metoprolol	MRI	↓	<0.01	0.99	②
[103]	mouse	45 min / 0 min	con vs iv-atorvastatin	TTC	↓	<0.05	1.00	
[103]	mouse	45 min / 0 min	con vs AMPK inhibition	TTC	↑	<0.05	0.79	
[103]	mouse	45 min / 0 min	con vs IV-ß-OH-simvastatin	TTC	↓	<0.05	1.00	
[103]	rat	perm / 30 d	con vs iv-atorvastatin	Histo	↓	<0.05	0.94	
[104]	rat	30 min / 120 min	con vs RIPC	TTC	↓	<0.05	0.90	
[104]	rat	30 min / 120 min	con vs UAG	TTC	↓	<0.05	0.93	
[104]	rat	30 min / 120 min	con vs RIPC +AG490	TTC	→	n.s.	0.13	
[104]	rat	30 min / 120 min	con vs UAG-AG490	TTC	→	n.s.	0.06	
[104]	rat	30 min / 120 min	RIPC vs RIPC +AG490	TTC	↑	<0.05	1.00	
[104]	rat	30 min / 120 min	UAG vs RIPC +AG490	TTC	↑	<0.05	1.00	
[104]	rat	30 min / 120 min	RIPC +AG490 vs UAG+AG490	TTC	→	n.s.	0.13	
[104]	rat	30 min / 120 min	RIPC vs UAG+AG490	TTC	↑	<0.05	0.97	
[104]	rat	30 min / 120 min	UAG vs UAG+AG490	TTC	↑	<0.05	0.98	
[105]	mouse	45 min / 3 d	renal innervated vs denervated	TTC	↓	<0.001	0.63	
[106]	rat	30 min / 120 min	veh vs bradykinin	TTC	↓	<0.05	0.81	
[106]	rat	30 min / 120 min	veh vs small extracellular vesicles	TTC	↓	<0.05	0.84	
[107]	mouse	perm / 1 wk	WT vs sFRP2 OE	Histo	↓	<0.05	0.99	

[107]	mouse	perm / 1 wk	sFRP2-WT vs sFRP2 OE+ABSEF	Histo	→	n.s.	0.33	
[107]	mouse	perm / 1 wk	sFRP2 OE vs sFRP2 OE+ABSEF	Histo	↑	<0.05	0.94	
[108]	pig	90 min / 2 d	con vs MSC	MRI	→	n.s.	0.80	
[108]	pig	90 min / 7 d	con vs MSC	MRI	↓	<0.05	1.00	
[109]	mouse	45 min / 0-24h	PKcs-f/f vs PKcs-cKO	TTC	↓	<0.05	1.00	
[109]	mouse	45 min / 0-24h	BL-1/DNA-PKcs-f/f vs DNA-PKcs-cKO	TTC	↓	<0.05	1.00	
[109]	mouse	45 min / 0-24h	DNA-PKcs-cKO vs DANN-PKcs/BL-1-cKO	TTC	↑	<0.05	1.00	
[109]	mouse	45 min / 0-24h	WT vs BL-1 OE	TTC	↓	<0.05	1.00	
[110]	mouse	45 min / 1 wk	scramble pept. vs TAG-23	TTC	↓	<0.0001	1.00	
[110]	mouse	45 min / 1 wk	scramble pept. vs TAG-23	Histo	↓	<0.05	1.00	
[111]	pig	60 min / 120 min	con vs DMX-10001	TTC	→	n.s.	0.55	
[111]	pig	60 min / 120 min	con vs DMX-10001 (w.o. outlier)	TTC	→	n.s.	0.62	
[112]	mouse	45 min / 120 min	con vs IPC	TTC	↓	<0.05	0.72	
[112]	mouse	45 min / 120 min	CX43-MAPKmut vs CX43-MAPKmut+IPC	TTC	↓	<0.05	1.00	
[112]	mouse	45 min / 120 min	CX43-PKCmut vs CX43-PKCmut+IPC	TTC	↓	<0.05	1.00	
[112]	mouse	45 min / 120 min	CX43-CK1mut-con vs CX43-CK1mut-IPC	TTC	→	n.s.	0.06	
[113]	mouse	30 min / 24 h	pla vs GUE1654-0.3 mg/kg	TTC	↓	<0.05	0.70	
[113]	mouse	30 min / 24 h	pla vs GUE1654-1 mg/kg	TTC	↓	<0.01	0.90	
[113]	mouse	30 min / 24 h	pla vs GUE1654-3 mg/kg	TTC	↓	<0.01	0.98	
[113]	mouse	30 min / 24 h	pla vs metoprolol	TTC	↓	<0.01	1.00	
[113]	mouse	30 min / 24 h	con vs BCAT1 OE	TTC	↓	<0.01	0.96	
[113]	mouse	30 min / 24 h	con vs BCAT2 OE	TTC	↓	<0.01	1.00	
[114]	mouse	45 min / 6 h	AAV9-NC vs AAV9-HINT2	TTC	↓	<0.05	0.97	
[114]	mouse	45 min / 6 h	AAV9-NC vs AAV9-NC+Ru360	TTC	↓	<0.05	1.00	
[114]	mouse	45 min / 6 h	AAV9-HINT2 vs AAV9-HINT2+spermine	TTC	↑	<0.05	0.75	
[115]	rat	30 min / 120 min	con vs PoCo	TTC	↓	<0.001	1.00	
[115]	rat	30 min / 120 min	con vs T3-100 µg	TTC	↓	<0.001	1.00	
[115]	rat	30 min / 120 min	con vs T3-200 µg	TTC	↓	<0.001	1.00	
[115]	rat	30 min / 120 min	con vs T3-300 µg	TTC	↓	<0.001	1.00	
[115]	rat	30 min / 120 min	con vs T3-500 µg	TTC	↓	<0.001	1.00	
[115]	rat	30 min / 120 min	PoCo vs T3-100 µg	TTC	↑	<0.01	1.00	
[115]	rat	30 min / 120 min	T3-300 µg vs T3-100 µg	TTC	↑	<0.01	1.00	
[115]	rat	30 min / 120 min	T3-500 µg vs T3-100 µg	TTC	↑	<0.01	0.98	
[115]	rat	30 min / 120 min	T3-200 µg vs T3-100 µg	TTC	→	n.s.	0.05	
[115]	rat	30 min / 120 min	RISK-BL vs T3-300 µg	TTC	↓	<0.001	1.00	
[115]	rat	30 min / 120 min	SAFE-BL vs T3-300 µg	TTC	↓	<0.001	1.00	
[115]	rat	30 min / 120 min	T3-300 µg+PI3K-BL vs T3-300 µg	TTC	↓	<0.001	1.00	
[115]	rat	30 min / 120 min	T3-300 µg+ERK-BL vs T3-300 µg	TTC	↓	<0.001	0.98	
[115]	rat	30 min / 120 min	T3-300 µg+RISK-BL vs T3-300 µg	TTC	↓	<0.001	1.00	
[115]	rat	30 min / 120 min	T3-300 µg+SAFE-BL vs T3-300 µg	TTC	→	n.s.	0.18	
[115]	rat	30 min / 120 min	con vs T3-300 µg+SAFE-BL	TTC	↓	<0.01	1.00	
[115]	rat	30 min / 120 min	RISK-BL vs T3-300 µg+SAFE-BL	TTC	↓	<0.01	1.00	
[115]	rat	30 min / 120 min	SAFE-BL vs T3-300 µg+SAFE-BL	TTC	↓	<0.01	1.00	
[115]	rat	30 min / 120 min	T3-300 µg-Pi3K-BL vs T3-300 µg+SAFE-BL	TTC	↓	<0.01	0.99	
[115]	rat	30 min / 120 min	T3-300 µg-ERK-BL vs T3-300 µg+SAFE-BL	TTC	↓	<0.01	0.88	
[115]	rat	30 min / 120 min	T3-300 µg-RISK-BL vs T3-300 µg+SAFE-BL	TTC	↓	<0.01	1.00	
[116]	mouse	perm / 8 wk	con vs LncHrt	Histo	↓	<0.05	0.73	
[117]	mouse	45 min / 120 min	empty vector vs overexpression vector	TTC	↑	<0.001	0.95	
[117]	mouse	45 min / 120 min	con vs ZIP7 cKO	TTC	↓	<0.001	1.00	
[117]	mouse	45 min / 120 min	con vs siZIP7	TTC	↓	<0.001	1.00	
[117]	mouse	45 min / 120 min	ZIP7 cKO vs ZIP7 cKO+siPINK1	TTC	↑	<0.001	1.00	
[117]	mouse	45 min / 120 min	ZIP7 cKO vs siPINK1	TTC	↑	<0.001	1.00	
[117]	mouse	45 min / 120 min	siZIP7 vs ZIP7 cKO+siPINK1	TTC	↑	<0.001	1.00	
[117]	mouse	45 min / 120 min	siZIP7 vs siPINK1	TTC	↑	<0.001	1.00	
[118]	mouse	perm / 28 d	WT vs NPPA-AS KO	Histo	↓	<0.05	1.00	
[119]	mouse	60 min / 6 wk	PBS vs clondronate	SPECT	↑	0.0496	0.68	
[120]	rat	25 min / 60 min	h RBC vs STEMI RBC	TTC	↓	<0.001	1.00	
[120]	rat	25 min / 60 min	STEMI RBC vs STEMI RBC+LNAME	TTC	↑	<0.01	1.00	
[120]	rat	25 min / 60 min	STEMI RBC vs STEMI RBC+ODQ	TTC	↑	<0.01	0.97	
[120]	rat	25 min / 60 min	STEMI RBC vs KT5823+STEMI RBC	TTC	↑	<0.001	1.00	
[120]	rat	25 min / 60 min	STEMI RBC vs STEMI RBC+MRS2211	TTC	↑	<0.01	1.00	
[120]	rat	25 min / 60 min	h RBC vs h RBC+mATP	TTC	↓	<0.001	1.00	
[120]	rat	25 min / 60 min	h RBC+mATP vs h RBC+mATP+ODQ	TTC	↑	<0.05	0.78	
[120]	rat	25 min / 60 min	h RBC+mATP vs h RBC+mATP+MRS211	TTC	↑	<0.001	1.00	
[121]	pig	60 min / 180 min	con vs IPC	TTC	→	n.s.	0.28	
[122]	mouse	30 min / 120 min	con vs EMPA	TTC	↓	<0.01	0.99	
[122]	mouse	30 min / 120 min	con vs DAPA	TTC	↓	<0.001	0.99	
[122]	mouse	30 min / 120 min	con vs ERTU	TTC	→	n.s.	0.56	
[122]	mouse	30 min / 120 min	con (2) vs stattic	TTC	→	n.s.	0.23	
[122]	mouse	30 min / 120 min	con (2) vs EMPA	TTC	↓	<0.001	0.99	
[122]	mouse	30 min / 120 min	EMPA vs EMPA+stattic	TTC	↑	<0.05	0.78	
[122]	mouse	30 min / 120 min	con (2) vs DAPA	TTC	↓	<0.001	0.98	
[122]	mouse	30 min / 120 min	stattic vs DAPA	TTC	↓	<0.01	0.92	
[122]	mouse	30 min / 120 min	DAPA vs DAPA+stattic	TTC	↑	<0.05	0.74	
[122]	mouse	30 min / 120 min	wortmannin vs EMPA	TTC	↓	<0.01	0.96	
[122]	mouse	30 min / 120 min	EMPA vs EMPA+wortmannin	TTC	→	n.s.	0.46	
[122]	mouse	30 min / 120 min	wortmannin vs DAPA	TTC	↓	<0.001	0.95	
[122]	mouse	30 min / 120 min	DAPA vs DAPA+wortmannin	TTC	↑	<0.05	0.77	
[123]	mouse	35 min / 24 h	con vs p-selectin deficiency	TTC	→	n.s.	0.11	
[124]	rat	30 min / 120 min	con vs FC	TTC	↓	<0.0001	0.96	
[124]	rat	30 min / 120 min	con vs HM3Dq	TTC	↑	<0.0001	1.00	
[124]	rat	30 min / 120 min	con vs HM4Di	TTC	↓	<0.0001	1.00	
[124]	rat	30 min / 120 min	con vs CPZ	TTC	↓	<0.0001	1.00	
[124]	rat	30 min / 120 min	con vs L70306	TTC	↓	<0.0001	0.91	
[124]	rat	30 min / 120 min	con vs CGPR8-37	TTC	↓	<0.0001	0.91	
[125]	mouse	30 min / 24 h	veh vs 0.15 mg ML-SI3	TTC	↓	<0.001	1.00	
[125]	mouse	30 min / 24 h	veh vs 1.5 mg ML-SI3	TTC	↓	<0.001	1.00	
[125]	mouse	30 min / 24 h	0.15 mg vs 1.5 mg ML-SI3	TTC	↓	<0.001	1.00	

[125]	mouse	30 min / 24 h	con vs ML-SI3	Histo	↓	<0.05	1.00	
[125]	mouse	30 min / 24 h	1D AAV-cTNT-vector vs 1D OE-AAV-cTNT-TRPML1	TTC	↑	<0.05	0.90	
[125]	mouse	30 min / 24 h	IR-1D AAV-cTNT-vector vs 1D OE-AAV-cTNT-TRPML1V432P	TTC	↑	<0.001	0.94	
[125]	mouse	30 min / 24 h	IR-1D AAV-cTNT-vector vs NAC-1D OE-AAV-cTNT-vector	TTC	↓	<0.001	1.00	
[125]	mouse	30 min / 24 h	IR-1D OE-AAV-cTNT-TRPML1 vs NAC-1D OE-AAV-cTNT-TRPML1	TTC	↓	<0.001	1.00	
[125]	mouse	30 min / 24 h	IR-1D OE-AAV-cTNT-TRPML1V432P vs NAC-1D OE-AAV-cTNT-TRPML1V432P	TTC	→	n.s.	0.23	
[126]	mouse	perm / 24 h	veh vs EMPA 10 mg	Histo	↓	<0.05	0.87	
[126]	mouse	perm / 24 h	veh vs EMPA 30 mg	Histo	↓	<0.001	1.00	
[126]	mouse	perm / 24 h	WT vs Nhe1 KO	TTC	↓	<0.05	0.73	
[126]	mouse	perm / 24 h	Nhe1 KO vs EMPA	TTC	→	n.s.	0.24	
[127]	mouse	30 min / 40 min	veh vs ARP	TTC	↓	0.026	0.98	
[127]	mouse	30 min / 40 min	veh vs ONO	TTC	↓	0.026	0.92	
[128]	rat	30 min / 28 d	con vs RIC	TTC	↓	<0.05	1.00	
[128]	rat	perm / 28 d	Exo vs Exo+RIC	TTC	↓	<0.05	1.00	
[129]	pig	60 min / 180 min	con vs metoprolol	TTC	→	n.s.	0.28	
[130]	rat	40 min / 120 min	pla vs imatinib	TTC	↓	0.002	0.96	
[130]	rat	45 min / 180 min	pla vs imatinib	TTC	↓	0.013	0.85	
[130]	rat	45 min / 180 min	pla vs imatinib	MRI	↓	0.008	0.90	
[131]	mouse	45 min / 24 h	pla vs rivaroxaban	TTC	→	n.s.	0.08	②
[132]	rat	perm / 7 d	saline vs CNO	Histo	↓	<0.05	0.80	
[133]	mouse	perm / 5 wk	WT vs Smyd1a OE	Histo	↓	<0.05	0.81	
[134]	mouse	perm / 28 d	Tet ON vs Tet OFF	Histo	↓	<0.05	0.21	
[135]	mouse	perm / 14 wk	WT vs GSK3a KO	Histo	↓	0.026	0.93	
[136]	mouse	40 min / 24 h	veh vs CY-09	TTC	↓	<0.01	0.57	
[136]	mouse	40 min / 24 h	CY-09 vs CY-09 delayed	TTC	↑	<0.05	0.33	
[136]	mouse	40 min / 24 h	WT vs NLRP3 -/-	TTC	↓	<0.001	1.00	
[136]	mouse	40 min / 24 h	SPLX vs WT-SPAT	TTC	↑	<0.05	0.69	
[136]	mouse	40 min / 24 h	WT SPAT vs NLRP3-/- SPAT	TTC	↓	<0.01	0.72	
[136]	mouse	40 min / 24 h	SPLX vs WT-SPAT+Spl-leuKO	TTC	↑	<0.001	0.93	
[136]	mouse	40 min / 24 h	WT SPAT vs NLRP3-/- SPAT +Spl leuKO	TTC	↑	<0.01	0.89	
[137]	mouse	perm / 28 d	WT vs CD34 KO	Histo	↓	<0.05	0.27	
[138]	mouse	30 min / 4 h	veh vs GI-Y1 25 mg/kg (2h pre)	TTC	↓	<0.001	0.84	
[138]	mouse	30 min / 4 h	veh vs GI-Y1 50 mg/kg (2h pre)	TTC	↓	<0.001	1.00	
[138]	mouse	30 min / 4 h	veh vs GI-Y1 50 mg/kg (1 min rep)	TTC	↓	<0.01	0.85	
[138]	mouse	30 min / 2 wk	veh vs GI-Y1 50 mg/kg (1 min rep)	TTC	↓	<0.001	0.94	
[138]	mouse	30 min / 4 h	WT vs Gsdmd KO	TTC	↓	<0.01	1.00	
[138]	mouse	30 min / 4 h	WT vs GSdmd KO+GI-Y1	TTC	↓	<0.01	0.98	

Cardiovascular Research

ref.	species	I/R timing	intervention	method	effect	p-value	power	note
[139]	rat	25 min / 120 min	sedentary vs excercise (untreated)	TTC	↓	<0.05	0.91	
[139]	rat	25 min / 120 min	exercise vs excercise+BCNU	TTC	↑	<0.05	1.00	
[139]	rat	25 min / 120 min	sedentary vs exercise	TTC	↓	<0.05	1.00	
[139]	rat	25 min / 120 min	sedentary vs excercise+bendavia	TTC	↓	<0.05	0.86	
[139]	rat	25 min / 120 min	sedentary vs excercise+moto TEMPO	TTC	↓	<0.05	1.00	
[140]	mouse	45 min / 24 h	WT vs TRPM2 KO	TTC	↓	<0.01	1.00	
[140]	mouse	60 min / 120 min	heart +/+ 0 vs heart +/+ PMN+/+	TTC	↑	<0.05	1.00	
[140]	mouse	60 min / 120 min	heart +/+ 0 vs heart +/+ PMN-/-	TTC	↑	<0.05	0.98	
[140]	mouse	60 min / 120 min	heart +/+ PMN+/+ vs heart +/+ PMN-/-	TTC	↓	<0.05	1.00	
[140]	mouse	60 min / 120 min	heart -/- 0 vs heart -/- PMN+/+	TTC	↑	<0.05	1.00	
[140]	mouse	60 min / 120 min	heart -/- 0 vs heart -/- PMN-/-	TTC	↑	<0.05	0.92	
[140]	mouse	60 min / 120 min	heart +/+ PMN+/+ vs heart -/- PMN-/-	TTC	↓	<0.05	1.00	
[140]	mouse	60 min / 120 min	heart +/+ PMN-/- vs heart -/- PMN-/-	TTC	↓	<0.05	0.54	
[140]	mouse	45 min / 120 min	mouse +/+ PMN 0 vs mouse -/- PMN 0	TTC	↓	<0.05	0.99	
[140]	mouse	45 min / 120 min	mouse -/- PMN 0 vs mouse +/+ PMN +/+	TTC	↑	<0.05	1.00	
[140]	mouse	45 min / 120 min	mouse -/- PMN 0 vs mouse +/+ PMN-/-	TTC	↑	<0.05	1.00	
[140]	mouse	45 min / 120 min	mouse -/- PMN 0 vs mouse +/+ PMN-/-	TTC	↑	<0.05	0.96	
[140]	mouse	45 min / 120 min	mouse -/- PMN +/+ vs mouse -/- PMN-/-	TTC	↓	<0.05	0.78	
[140]	mouse	45 min / 120 min	mouse +/+ PMN-/- vs mouse -/- PMN-/-	TTC	↓	<0.05	0.99	
[140]	mouse	45 min / 120 min	mouse -/- PMN 0 vs mouse -/- PMN +/+	TTC	↑	<0.05	0.98	
[140]	mouse	45 min / 120 min	mouse -/- PMN +/+ vs mouse -/- PMN -/-	TTC	↑	<0.05	0.05	
[141]	mouse	30 min / 24 h	con vs Nucleolin OE Line52	TTC	↓	<0.05	0.37	
[141]	mouse	30 min / 24 h	con vs Nucleolin OE Line86	TTC	↓	<0.05	0.81	
[141]	mouse	30 min / 24 h	con vs nucleolin OE	TTC	↓	<0.05	0.99	
[141]	mouse	30 min / 24 h	Nucleolin OE vs Nucleolin OE+ZnPP	TTC	↑	<0.05	0.77	
[142]	mouse	perm / 24 h	WT vs human hspa12b gene OE	TTC	↓	<0.01	1.00	
[143]	mouse	perm / 14 d	saline vs parthenogenetic ESCs	Histo	→	n.s.	0.80	
[144]	mouse	perm / 24 h	WT vs CD13 KO	TTC	→	n.s.	0.05	
[145]	mouse	35 min / 60 min	WT vs ASC KO	TTC	→	n.s.	0.14	
[145]	mouse	35 min / 60 min	WT vs NLRP3 KO	TTC	↓	<0.05	0.77	
[146]	rat	perm / 8 wk	con vs SV-rSkM	Histo	→	n.s.	0.06	
[146]	rat	perm / 8 wk	WT-rSkM vs SV-rSkM	Histo	↓	<0.05	0.62	
[147]	mouse	60 min / 240 min	LmiR-con vs LmiR146a	TTC	↓	<0.05	0.99	
[147]	mouse	60 min / 240 min	con vs LmiR146a	TTC	↓	<0.05	1.00	
[148]	rat	35 min / 60 min	con vs IPC (3mo db Goto-Kakisaki)	TTC	↓	<0.005	1.00	
[148]	rat	35 min / 60 min	con vs IPC (8mo db Goto-Kakisaki)	TTC	↓	<0.005	0.97	
[148]	rat	35 min / 60 min	con vs IPC (12mo db Goto-Kakisaki)	TTC	→	n.s.	0.18	
[148]	rat	35 min / 60 min	con vs IPC (18mo db Goto-Kakisaki)	TTC	→	n.s.	0.36	
[148]	rat	35 min / 60 min	con vs IPC (3mo nd Wistar)	TTC	↓	<0.001	1.00	
[148]	rat	35 min / 60 min	con vs IPC (8mo nd Wistar)	TTC	↓	<0.005	0.99	
[148]	rat	35 min / 60 min	con vs IPC (12mo nd Wistar)	TTC	↓	<0.001	1.00	
[148]	rat	35 min / 60 min	con vs IPC (18mo nd Wistar)	TTC	↓	<0.005	0.71	
[149]	rat	30 min / 120 min	con vs IPC	TTC	↓	<0.01	1.00	
[149]	rat	30 min / 120 min	IPC vs IPC +L-NAME	TTC	↑	<0.01	1.00	
[149]	rat	30 min / 120 min	IPC vs IPC +LY	TTC	↑	<0.01	1.00	
[149]	rat	30 min / 120 min	IPC vs IPC +H89	TTC	↑	<0.01	1.00	
[150]	rat	perm / 1 wk	EV-F vs EV-CPC-HD	Histo	↓	<0.05	0.47	
[150]	rat	perm / 1 wk	PBS vs EV-CPC-HD	Histo	↓	<0.05	0.19	

[151]	rat	35 min / 120 min	con vs BAY41-2272 3µ	TTC	↓	<0.05	0.91
[151]	rat	35 min / 120 min	con vs BAY41-2272 3µ LNAME	TTC	↓	<0.05	0.76
[151]	rat	35 min / 120 min	con vs BAY41-2272 3µ+CPTIO	TTC	↓	<0.05	0.47
[151]	rat	35 min / 120 min	con vs NOC9 1 n	TTC	↓	<0.05	0.64
[151]	rat	35 min / 120 min	con vs NOC9 10 n	TTC	↓	<0.01	0.95
[151]	rat	35 min / 120 min	con vs NOC9 100 n	TTC	↓	<0.001	1.00
[151]	rat	35 min / 120 min	con vs NOC9 1 µ	TTC	↓	<0.001	1.00
[151]	rat	35 min / 120 min	con vs BAY60-2770 5 n	TTC	↓	<0.01	0.86
[151]	rat	35 min / 120 min	con vs BAY60-2770 500 n	TTC	↓	<0.01	0.87
[151]	rat	35 min / 120 min	con vs BAY60-2770 50 n	TTC	↓	<0.01	0.75
[151]	rat	35 min / 120 min	con vs BAY60-2770 1 µ	TTC	↓	<0.01	0.76
[151]	rat	35 min / 120 min	con vs BAY60+ODQ	TTC	↓	<0.01	0.96
[151]	rat	35 min / 120 min	con vs BAY60+C-PTIO	TTC	↓	<0.01	0.70
[151]	rat	35 min / 120 min	con vs BAY60+BAY41	TTC	↓	<0.05	0.49
[152]	mouse	30 min / 24 h	WT vs C5aR -/-	TTC	↓	0.017	0.69
[152]	mouse	30 min / 24 h	WT/WT BM vs C5aR-/- C5aR-/- BM	TTC	↓	0.05	0.66
[152]	mouse	30 min / 24 h	WT/WT BM vs WT C5aR-/- BM	TTC	↓	0.05	0.92
[153]	mouse	30 min / 24 h	SENEP +/+ vs SENEP +/-	TTC	↑	<0.01	0.95
[154]	mouse	30 min / 24 h	WT vs TMLeD/LeD	TTC	↑	<0.01	0.97
[154]	mouse	30 min / 24 h	WT+hydroxycarbamide vs TMLeD/LeD+hydroxycarbamide	TTC	↑	<0.01	1.00
[155]	rat	40 min / 2 h	con vs PoCo	TTC	↓	<0.05	1.00
[155]	rat	40 min / 2 h	con vs HMR1766 100nM	TTC	↓	<0.05	0.91
[155]	rat	40 min / 2 h	kbr7943 vs PoCo+KBR7943	TTC	↑	<0.05	1.00
[155]	rat	40 min / 2 h	con vs Ataciugat 0.1 ug/kg	TTC	↓	<0.05	0.78
[155]	rat	40 min / 2 h	con vs thapsigarin	TTC	↓	<0.05	1.00
[155]	rat	40 min / 2 h	con vs KT5720	TTC	↓	<0.05	0.96
[155]	rat	40 min / 2 h	con vs KN93	TTC	↓	<0.05	1.00
[155]	rat	40 min / 2 h	PoCo vs PoCo+KT5823	TTC	↑	<0.05	1.00
[156]	rat	30 min / 24 h	con vs IPC	TTC	↓	<0.05	0.94
[156]	rat	30 min / 24 h	nucleolin OEneg vs nucleolin Oeneg+IPC	TTC	↓	<0.05	0.98
[156]	rat	30 min / 24 h	nucleolin OE vs nucleolin OE+IPC	TTC	→	n.s.	0.25
[156]	rat	30 min / 24 h	nucleolin OEneg vs nucleolin OE	TTC	↓	<0.05	0.94
[157]	mouse	30 min / 24 h	sham vs early IPC (WT)	TTC	↓	<0.05	1.00
[157]	mouse	30 min / 24 h	sham vs late IPC (WT)	TTC	↓	<0.05	1.00
[157]	mouse	30 min / 24 h	sham vs early IPC (NOX1-/y)	TTC	↓	<0.05	0.99
[157]	mouse	30 min / 24 h	sham vs late IPC (NOX1-/y)	TTC	→	n.s.	0.16
[158]	mouse	perm / 14 d	PBS vs EPO-primed mob-PBMC	Histo	↓	<0.05	1.00
[158]	mouse	perm / 14 d	veh-primed mob-PBMC vs EPO-primed mob-PBMC	Histo	↓	<0.05	0.89
[159]	mouse	30 min / 24 h	fem XX vs fem XY (gonadectomized)	TTC	↓	<0.01	0.90
[159]	mouse	30 min / 24 h	fem XX vs male XY (gonadectomized)	TTC	↓	<0.05	0.97
[159]	mouse	30 min / 24 h	male XX vs female XY (gonadectomized)	TTC	↓	<0.05	0.64
[159]	mouse	30 min / 24 h	male XX vs male XY (gonadectomized)	TTC	↓	<0.05	0.85
[159]	mouse	30 min / 60 min	fem XX vs fem XY (gonadectomized)	TTC	↓	<0.05	0.79
[159]	mouse	30 min / 60 min	fem XX vs male XY (gonadectomized)	TTC	↓	<0.05	0.88
[159]	mouse	30 min / 60 min	male XX vs female XY (gonadectomized)	TTC	↓	<0.05	0.51
[159]	mouse	30 min / 60 min	male XX vs male XY (gonadectomized)	TTC	↓	<0.05	0.65
[159]	mouse	30 min / 60 min	fem XX vs fem X0	TTC	↓	<0.05	0.43
[159]	mouse	30 min / 60 min	fem XX vs male XY	TTC	↓	<0.05	0.34
[159]	mouse	30 min / 60 min	male XXY vs female X0	TTC	↓	<0.05	0.58
[159]	mouse	30 min / 60 min	male XXY vs male XY	TTC	↓	<0.05	0.47
[160]	mouse	30 min / 28 d	No DOX vs DOX pre MI (rtTA-CnAß1 mice)	Histo	↓	<0.05	0.71
[160]	mouse	30 min / 28 d	No DOX vs DOX post-MI (rtTA-CnAß1 mice)	Histo	↓	<0.05	0.90
[160]	mouse	30 min / 28 d	NoDox vs Dox pre MI (rtTA mice)	Histo	→	n.s.	0.08
[161]	mouse	perm / 24 h	WT vs RIP3 -/-	MRI	→	n.s.	0.11
[162]	mouse	30 min / 24 h	Tie2Cre+ApoE-/- vs Cx40-del	TTC	↑	<0.05	0.85
[162]	mouse	30 min / 24 h	Cx40fl/flApoE-/- vs Cx40-del	TTC	↑	<0.05	0.81
[162]	mouse	30 min / 24 h	Cx40-del vs Cx40-del+methotrexate	TTC	↓	<0.05	0.98
[163]	rat	30 min / 24 h	DMSO vs GSK360A	TTC	↓	<0.05	0.89
[163]	rat	30 min / 24 h	DMSO vs GSK360A+ctrl peptide	TTC	→	n.s.	0.25
[163]	mouse	30 min / 24 h	WT vs MHC-cre tamoxifen	TTC	↓	<0.05	0.90
[164]	rat	20 min / 120 min	nitrite vs nitrite+pki	TTC	↑	<0.01	0.99
[165]	mouse	60 min / 28 d	WT vs CXCR3 KO	Histo	→	n.s.	0.11
[166]	mouse	45 min / 1 wk	con vs miR-con	TTC	→	n.s.	0.07
[166]	mouse	45 min / 1 wk	con vs miR-125b	TTC	↓	<0.05	1.00
[166]	mouse	45 min / 1 wk	WT vs miR-125b OE	TTC	↓	<0.05	1.00
[166]	mouse	45 min / 1 wk	WT vs anti miR-125	TTC	↑	<0.05	0.73
[166]	mouse	45 min / 1 wk	anti miR con vs anti miR-125b	TTC	↑	<0.05	0.88
[167]	mouse	perm / 28 d	WT vs E2F1 -/-	Histo	↓	<0.01	1.00
[168]	mouse	perm / 4 wk	MI vs MI+AdsiScr	Histo	→	n.s.	0.09
[168]	mouse	perm / 4 wk	MI vs MI+AdsiPeli1	Histo	↓	<0.05	0.97
[169]	pig	40 min / 120 min	con vs RIC	TTC	↓	<0.001	0.99
[169]	pig	40 min / 120 min	con vs GIK	TTC	↓	<0.001	0.93
[169]	pig	40 min / 120 min	con vs RIC+GIK	TTC	↓	<0.001	1.00
[169]	pig	40 min / 120 min	con vs exenatide	TTC	↓	<0.001	1.00
[169]	pig	40 min / 120 min	con vs RIC+exenatide	TTC	↓	<0.001	1.00
[170]	mouse	45 min / 3 h	WT vs Rip22/2 KO	TTC	↑	<0.05	0.60
[171]	mouse	perm / 25 wk	WT vs FLNA KO	Histo	↑	<0.001	0.97
[172]	rabbit	30 min / 180 min	con vs PoCo	TTC	↓	<0.05	1.00
[172]	rabbit	30 min / 180 min	PoCo vs NaHs	TTC	↓	<0.05	1.00
[172]	rabbit	30 min / 180 min	PoCo vs PoCo+NaHS	TTC	↓	<0.05	1.00
[172]	rabbit	30 min / 180 min	con vs NaHS	TTC	↓	<0.05	1.00
[172]	rabbit	30 min / 180 min	con vs NaHs+TAT	TTC	↓	<0.05	1.00
[172]	rabbit	30 min / 180 min	con vs PoCo +TAT	TTC	↓	<0.05	1.00
[172]	rabbit	30 min / 180 min	con vs NaHS+5HD	TTC	↓	<0.05	1.00
[172]	rabbit	30 min / 180 min	con vs NaHS+glibenclamide	TTC	↓	<0.05	1.00
[172]	rabbit	30 min / 180 min	con vs NaHS+LNaME	TTC	↓	<0.05	1.00
[172]	mouse	30 min / 120 min	con vs PoCo 3x10s	TTC	↓	<0.05	1.00
[172]	mouse	30 min / 120 min	con vs PoCo 6x10s	TTC	↓	<0.05	1.00

[172]	mouse	30 min / 120 min	con vs NaHS	TTC	↓	<0.05	1.00	
[172]	mouse	30 min / 120 min	con vs NaHS	TTC	↓	<0.05	1.00	
[173]	rat	30 min / 120 min	con vs H2O2-PC	TTC	↓	<0.01	1.00	
[173]	rat	30 min / 120 min	con vs UCP3 OE	TTC	↓	<0.01	0.99	
[173]	rat	30 min / 120 min	H2O2-PC vs UCP3+H2O2-PC	TTC	↑	<0.05	0.98	
[173]	rat	30 min / 120 min	H2O2-PC vs H2O2-PC+ATR	TTC	↑	<0.05	0.95	
[173]	rat	30 min / 120 min	H2O2-PC vs UCP3 OE+ATR	TTC	↑	<0.01	0.95	
[173]	rat	30 min / 120 min	H2O2-PC vs H2O2-PC+WM	TTC	↑	<0.05	0.94	
[173]	rat	30 min / 120 min	UCP3 OE vs UCP3 OE+WM	TTC	↑	<0.05	0.60	
[173]	rat	30 min / 120 min	WM vs WM+H2O2-PC	TTC	↓	<0.05	0.73	
[173]	rat	30 min / 120 min	WM vs WM+UCP3 OE	TTC	↓	<0.05	0.75	
[174]	mouse	30 min / 120 min	Veh vs NED-K	TTC	↓	<0.05	0.80	
[174]	mouse	30 min / 120 min	WT vs TCP1 KO	TTC	↓	<0.05	0.86	
[175]	mouse	30 min / 24 h	con vs LSN2792613	TTC	↓	0.03	0.73	
[176]	mouse	30 min / 60 min	con vs renalase	TTC	↓	<0.05	1.00	
[176]	mouse	30 min / 60 min	con vs renalase siRNA	TTC	↓	<0.05	0.96	
[176]	mouse	30 min / 60 min	con vs HIF1a siRNA	TTC	↑	<0.05	1.00	
[176]	mouse	30 min / 60 min	con vs HIF1a siRNA+veh	TTC	↑	<0.05	1.00	
[176]	mouse	30 min / 60 min	con vs HIF1a siRNA+renalase	TTC	↓	<0.05	0.98	
[177]	mouse	perm / 24 h	con vs chronic hind limb ischemia	TTC	↑	<0.05	1.00	
[177]	mouse	perm / 24 h	con vs chronic hind limb ischemia	Histo	↑	<0.05	0.89	
[178]	mouse	perm / 48 h	WT vs Gadd45 KO	TTC	→	n.s.	0.22	
[179]	rat	30 min / 120 min	con vs RIPER	TTC	↓	<0.001	1.00	
[179]	rat	30 min / 120 min	RIPER vs cervical vagotomy+RIPER	TTC	↑	<0.01	1.00	
[179]	rat	30 min / 120 min	RIPER vs subdiaphragmatic vagotomy+RIPER	TTC	↑	<0.01	1.00	
[179]	rat	30 min / 120 min	con vs IPC	TTC	↓	<0.001	1.00	
[179]	rat	30 min / 120 min	con vs IPC +Exendin(9-39)	TTC	↓	<0.001	1.00	
[179]	rat	30 min / 120 min	con vs RIPC	TTC	↓	<0.001	1.00	
[179]	rat	30 min / 120 min	con vs RIPER	TTC	↓	<0.001	1.00	
[179]	rat	30 min / 120 min	RIPC vs RIPC+Exendin(9-39)	TTC	↑	<0.01	0.97	
[179]	rat	30 min / 120 min	RPER vs RPER+Exendin(9-39)	TTC	↑	<0.01	1.00	
[180]	mouse	45 min / 24 h	AKIP OE vs WT	TTC	↓	<0.05	0.80	
[181]	pig	75 min / 1 wk	rADAMTS13 vs con	Histo	→	n.s.	0.32	
[182]	mouse	perm / 28 d	MI vs MI+BAPN	Histo	↓	<0.01	0.61	
[183]	mouse	45 min / 180 min	KCNE2 +/+ vs KCNE2 -/-	TTC	↓	<0.05	0.78	
[183]	mouse	45 min / 180 min	KCNE2 +/+ vs KCNE2 -/-	TTC	↓	<0.05	0.88	
[183]	mouse	45 min / 180 min	KCNE2 +/+ vs KCNE2 +/+ +SB216763	TTC	↓	<0.05	0.77	
[183]	mouse	45 min / 180 min	KCNE2 +/+ vs KCNE2 -/- +SB216763	TTC	↓	<0.05	0.84	
[184]	mouse	perm / 28 d	WT vs IL-12p35 KO	Histo	↓	<0.05	1.00	
[185]	mouse	perm / 14 d	WT vs LRG KO	Histo	↑	<0.01	0.92	
[186]	rat	perm / 4 wk	veh+MI+rAAV9 vs SEN195+MI+rAAV9	Histo	↓	<0.01	0.99	
[187]	mouse	30 min / 24 h	WT vs ApoM OE	TTC	↓	0.01	0.93	
[187]	mouse	30 min / 60 min	con vs S1P	TTC	↓	0.01	1.00	
[188]	mouse	20 min / 90 min	con vs NaHS	TTC	↓	<0.01	1.00	
[188]	mouse	20 min / 90 min	con vs SNAP	TTC	↓	<0.01	1.00	
[188]	mouse	20 min / 90 min	con vs NaHS+SNAP	TTC	↓	<0.01	1.00	
[188]	mouse	20 min / 90 min	con vs 10uM SNAP	TTC	↓	<0.05	0.64	
[188]	mouse	20 min / 90 min	con vs 100uM NaHS	TTC	↓	<0.05	0.69	
[188]	mouse	20 min / 90 min	con vs 10uM SNAP+100uM NaHS	TTC	↓	<0.01	0.96	
[188]	mouse	20 min / 90 min	10uM SNAP vs 10uM SNAP+100uM NaHS	TTC	↓	<0.05	0.89	
[188]	mouse	20 min / 90 min	100uM NaHS vs 10uM SNAP+100uM NaHS	TTC	↓	<0.05	0.75	
[188]	mouse	20 min / 90 min	10uM SNAP+100uM NaHS vs 10uM SNAP+100uM NaHS+C-PTIO	TTC	↑	<0.01	0.99	
[189]	mouse	perm / 4 wk	MI+ND vs MI+MK-0626	Histo	→	n.s.	0.29	
[190]	mouse	35 min / 60 min	con vs malonate	TTC	↓	0.001	0.99	
[191]	mouse	35 min / 60 min	BL plasma vs postRIC plasma	TTC	↓	<0.05	0.79	
[191]	mouse	35 min / 60 min	con vs glycine 3mmol	TTC	↓	<0.05	0.90	
[192]	mouse	perm / 1 wk	anti-mIR con vs anti miR-5325p	Histo	↑	<0.001	1.00	
[193]	mouse	30 min / 120 min	con vs PF431396A	TTC	↓	<0.05	1.00	
[193]	mouse	30 min / 120 min	con vs PF431396B	TTC	↓	<0.05	1.00	
[194]	mouse	perm / 24 h	WT vs periostin KO	TTC	→	n.s.	0.05	
[195]	mouse	perm / 28 d	scramble vs LNA-anti miR-375	Histo	↓	<0.001	1.00	
[196]	rat	30 min / 120 min	saline vs heparin	TTC	↓	<0.05	0.95	②
[196]	rat	30 min / 120 min	saline vs FGF1	TTC	↓	<0.05	1.00	②
[196]	rat	30 min / 120 min	saline vs heparin+FGF1	TTC	↓	<0.05	1.00	②
[196]	rat	30 min / 120 min	saline vs FGF1DHBS	TTC	↓	<0.05	1.00	②
[196]	rat	30 min / 120 min	saline vs FGF1DHBS +heparin	TTC	↓	<0.05	1.00	②
[196]	rat	30 min / 120 min	heparin vs FGF1	TTC	↓	<0.05	0.63	②
[196]	rat	30 min / 120 min	heparin vs FGF1DHBS	TTC	↓	<0.05	1.00	②
[196]	rat	30 min / 120 min	FGF1 vs FGF1DHBS	TTC	↓	<0.05	0.86	②
[196]	rat	30 min / 120 min	FGF1 vs FGF1DHBS +heparin	TTC	↓	<0.05	0.14	②
[196]	rat	30 min / 120 min	FGF1+heparin vs FGF1	TTC	↓	<0.05	0.42	②
[196]	rat	30 min / 120 min	FGF1+heparin vs FGF1DHBS	TTC	↓	<0.05	1.00	②
[196]	rat	30 min / 120 min	FGF1+heparin vs FGF1DHBS+heparin	TTC	↓	<0.05	0.84	②
[197]	rat	30 min / 24 h	con vs SNJ-1945	TTC	↓	0.005	0.94	
[198]	mouse	30 min / 24 h	con vs serelaxin pretreatment	TTC	↓	<0.05	1.00	
[198]	mouse	30 min / 24 h	con vs serelaxin pretreatment (eNOS KO)	TTC	→	n.s.	0.43	
[198]	mouse	30 min / 24 h	con vs eNOS KO	TTC	→	n.s.	0.42	
[198]	mouse	30 min / 24 h	con vs serelaxin with reperfusion	TTC	↓	<0.05	1.00	
[198]	mouse	30 min / 24 h	con vs serelaxin with reperfusion (eNOS KO)	TTC	→	n.s.	0.29	
[198]	mouse	30 min / 24 h	con vs eNOS KO	TTC	→	n.s.	0.29	
[199]	mouse	perm / 28 d	Exo ct vs Exo isch	Histo	→	n.s.	0.06	
[200]	mouse	40 min / 24 h	con vs glutamate	TTC	↓	0.0063	1.00	
[200]	mouse	40 min / 24 h	glutamate vs glutamate+YM298198	TTC	→	n.s.	0.05	
[200]	mouse	40 min / 24 h	con vs DHPG	TTC	↓	<0.01	1.00	
[200]	mouse	40 min / 24 h	DHPG vs DHPG+YM298198	TTC	→	n.s.	0.27	
[200]	mouse	40 min / 60 min	con vs PoCo	TTC	↓	<0.0001	1.00	
[200]	mouse	40 min / 60 min	con vs DHPH	TTC	↓	<0.05	1.00	
[200]	mouse	40 min / 60 min	con vs DHPH+PoCo	TTC	↓	<0.01	1.00	

[200]	mouse	40 min / 60 min	con vs PoCo +YM298198	TTC	→	n.s.	0.44
[201]	mouse	30 min / 120 min	WT vs Arrb2 KO	TTC	↓	<0.01	0.98
[201]	mouse	30 min / 120 min	WT vs Arrb2 OE	TTC	↑	<0.05	0.83
[202]	mouse	perm / 14 d	con vs topiramate	TTC	→	n.s.	0.09
[203]	rat	60 min / 28 d	Exo-F vs Exo-BMC	Histo	↓	<0.01	0.86
[203]	rat	60 min / 28 d	Exo-CpC vs Exo-BMC	Histo	↑	<0.05	0.21
[203]	rat	60 min / 28 d	Exo-CpC vs Exo-F	Histo	↑	<0.01	0.97
[204]	rat	perm / 28 d	PBS vs EV	Histo	↑	<0.05	0.88
[204]	rat	perm / 28 d	PBS vs STG+EV	Histo	↑	<0.01	1.00
[204]	rat	perm / 28 d	PBS vs STG+EPC	Histo	↑	<0.01	1.00
[204]	rat	perm / 28 d	STG vs STG+EV	Histo	↑	<0.01	0.98
[204]	rat	perm / 28 d	STG vs STG+EPC	Histo	↑	<0.01	0.99
[205]	mouse	30 min / 180 min	con vs RIPC (STAT5 fl/fl)	TTC	↓	<0.01	1.00
[206]	mouse	perm / 1 wk	con vs tb4	MRI	→	n.s.	0.54
[207]	mouse	perm / 1 wk	saline vs EGF-A	Histo	↓	<0.05	0.99
[207]	mouse	perm / 1 wk	saline vs Pep2-8	Histo	↓	<0.05	0.99
[207]	mouse	perm / 1 wk	WT vs PCSK9 -/-	Histo	↓	<0.05	0.97
[208]	mouse	30 min / 120 min	WT vs pkn1 KO	TTC	↑	<0.001	1.00
[209]	mouse	30 min / 120 min	con vs PoCo (CM NO-GC +/fl)	TTC	↓	<0.001	1.00
[209]	mouse	30 min / 120 min	PoCo (CM NO-GC +/fl) vs PoCo (CM NO-GC -/fl)	TTC	↑	<0.001	1.00
[209]	mouse	30 min / 120 min	PoCo+cinaciugat (CM NO-GC +/fl) vs PoCo+cinaciugat (CM NO-GC -/fl)	TTC	↑	<0.001	1.00
[209]	mouse	30 min / 120 min	PoCo+sildenafil (CM NO-GC +/fl) vs PoCo +sildenafil (CM NO-GC -/fl)	TTC	↑	<0.001	1.00
[209]	mouse	30 min / 120 min	PoCo+TAD (CM NO-GC +/fl) vs PoCo+TAD (CM NO-GC -/fl)	TTC	↑	<0.001	1.00
[209]	mouse	30 min / 120 min	con vs NS11021 (+/fl)	TTC	↓	<0.001	1.00
[209]	mouse	30 min / 120 min	con vs NS11021 (-/fl)	TTC	↓	<0.001	1.00
[210]	mouse	35 min / 60 min	con vs acetaldehyde	TTC	↓	<0.05	0.89
[210]	mouse	35 min / 60 min	ALDH2*2 KI vs acetaldehyde	TTC	↓	<0.05	0.59
[210]	mouse	35 min / 60 min	WT vs ALDH2*2 KI+acetaldehyde	TTC	↑	<0.05	0.79
[210]	mouse	35 min / 60 min	ALDH2*2 KI vs ALDH2*2 KI +acetaldehyde	TTC	↑	<0.05	0.87
[210]	mouse	35 min / 60 min	con vs ETOH	TTC	↓	<0.05	0.97
[210]	mouse	35 min / 60 min	ALDH2*2 KI vs WT+ ETOH	TTC	↓	<0.05	1.00
[210]	mouse	35 min / 60 min	WT vsALDH2*2 KI + ETOH	TTC	↑	<0.05	0.62
[210]	mouse	35 min / 60 min	ALDH2*2 KI vs ALDH2*2 KI + ETOH	TTC	↑	<0.05	0.55
[210]	mouse	35 min / 60 min	WT vs WT+ ETOH+ALDA	TTC	↓	<0.05	0.91
[210]	mouse	35 min / 60 min	ALDH2*2 KI vs WT+ ETOH+ALDA	TTC	↓	<0.05	0.99
[210]	mouse	35 min / 60 min	WT vsALDH2*2 KI + ETOH+ALDA	TTC	↓	<0.05	0.46
[210]	mouse	35 min / 60 min	ALDH2*2 KI vs ALDH2*2 KI + ETOH*ALDA	TTC	↓	<0.05	0.75
[210]	mouse	35 min / 60 min	ALDH2*2 KI +ETOH vs ALDH2*2 KI +ETOH+ALDA	TTC	↓	<0.05	1.00
[210]	mouse	35 min / 60 min	con vs CVT	TTC	↑	<0.05	1.00
[210]	mouse	35 min / 60 min	CVT vs con ETOH	TTC	↓	<0.05	1.00
[210]	mouse	35 min / 60 min	CVT vs CVT+ETOH	TTC	↑	<0.05	1.00
[211]	pig	60 min / 3 d	con vs clopidogrel	MRI	↓	<0.05	1.00
[211]	pig	60 min / 3 d	con vs ticagrelor	MRI	↓	<0.05	1.00
[211]	pig	60 min / 42 d	con vs clopidogrel	MRI	→	n.s.	0.78
[211]	pig	60 min / 42 d	con vs ticagrelor	MRI	↓	<0.05	1.00
[211]	pig	60 min / 3 d	clopidogrel vs ticagrelor	MRI	↓	<0.05	1.00
[211]	pig	60 min / 3 d	clopidogrel vs ticagrelor	MRI	↓	<0.05	1.00
[211]	pig	60 min / 42 d	3d vs 42d (con)	MRI	↓	<0.05	1.00
[211]	pig	60 min / 42 d	3d vs 42d (clopidogrel)	MRI	↓	<0.05	1.00
[211]	pig	60 min / 42 d	3d vs 42d (ticagrelor)	MRI	↓	<0.05	1.00
[211]	pig	60 min / 42 d	con vs clopidogrel	TTC	→	n.s.	0.87
[211]	pig	60 min / 42 d	con vs ticagrelor	TTC	↓	<0.05	1.00
[211]	pig	60 min / 42 d	clopidogrel vs ticagrelor	TTC	↓	<0.05	1.00
[212]	mouse	45 min / 24 h	young vs aged	TTC	↑	<0.05	0.44
[212]	mouse	45 min / 24 h	young vs aged in SIRT1 KO	TTC	↑	<0.05	0.32
[212]	mouse	45 min / 24 h	young vs aged	TTC	↑	<0.05	0.43
[212]	mouse	45 min / 24 h	aged vs aged+AAVSirt1	TTC	↓	<0.05	0.36
[212]	mouse	45 min / 24 h	young vs aged (veh)	TTC	↑	<0.05	0.93
[212]	mouse	45 min / 24 h	young veh vs aged A769662	TTC	↑	<0.05	0.70
[212]	mouse	45 min / 24 h	young A769223 vs aged A769662	TTC	↑	<0.05	0.35
[212]	mouse	45 min / 24 h	aged veh vs aged A769662	TTC	↓	<0.05	0.60
[212]	mouse	45 min / 24 h	veh vs A769662 (icSIRT1 KO)	TTC	↓	<0.05	0.70
[213]	mouse	20 min / 60 min	WT vs MtCK OE	TTC	↓	0.045	0.40
[213]	mouse	45 min / 24 h	WT vs MtCK OE	TTC	↓	<0.0001	1.00
[214]	mouse	perm / 8 wk	WT vs apn KO	Histo	→	n.s.	0.71
[215]	mouse	perm / 14 d	con vs sympathetic denervation	Histo	→	n.s.	0.08
[216]	mouse	30 min / 120 min	veh vs NTG A	TTC	↓	<0.01	1.00
[216]	mouse	30 min / 120 min	NTG A vs NTG-tolerant	TTC	↑	<0.01	1.00
[216]	mouse	30 min / 120 min	veh vs NTG B	TTC	↓	<0.05	1.00
[216]	mouse	30 min / 120 min	veh vs CypD-/-	TTC	↓	<0.01	1.00
[216]	mouse	30 min / 120 min	veh vs CypD-/- +NTG B	TTC	↓	<0.001	1.00
[216]	mouse	30 min / 120 min	NTG B vs eNOS-/-	TTC	↑	<0.001	1.00
[216]	mouse	30 min / 120 min	NTG B vs eNOS-/- +NTG B	TTC	↑	<0.01	1.00
[216]	mouse	30 min / 120 min	veh vs ApOE -/- +NTG B	TTC	↓	<0.01	1.00
[216]	mouse	30 min / 120 min	NTG B vs ApOE-/-	TTC	↑	<0.05	1.00
[216]	mouse	30 min / 120 min	ApOE-/- vs ApOE -/- +NTG B	TTC	↓	<0.01	1.00
[217]	mouse	perm / 1 wk	p110βFlx vs p110β-Tie2	TTC	↓	<0.05	0.94
[217]	mouse	perm / 24 h	p110ß-aMHC vs P110ß-FLX	TTC	↑	<0.05	0.71
[217]	mouse	perm / 1 wk	p110ß-aMHC vs P110ß-FLX	TTC	↑	<0.05	0.53
[218]	mouse	30 min / 24 h	saline vs TAK-242 1 mg	TTC	→	n.s.	0.30
[218]	mouse	30 min / 24 h	saline vs TAK-242-NP 3 mg	TTC	↓	<0.001	1.00
[219]	mouse	45 min / 120 min	con vs HC067047	TTC	↓	<0.05	0.70
[220]	mouse	perm / 3 mo	WT vs tenascin-c KO	Histo	→	n.s.	0.20
[221]	mouse	45 min / 24 h	con vs AM-001 8 mg	TTC	↓	<0.01	0.94
[221]	mouse	45 min / 24 h	veh WT vs AM-001 8 mg WT	TTC	↓	<0.001	1.00
[221]	mouse	45 min / 24 h	veh WT vs veh Epac1 -/-	TTC	↓	<0.001	1.00
[221]	mouse	45 min / 24 h	veh WT vs AM-001 8 mg Epac1 -/-	TTC	↓	<0.001	1.00
[222]	mouse	30 min / 120 min	WT vs TGSGLT1-KO	TTC	↓	<0.05	0.85

[222]	mouse	20 min / 120 min	WT vs TGSGLT1 OE	TTC	↓	<0.05	0.34
[223]	mouse	perm / 5 d	con vs cxcl4	TTC	→	n.s.	0.07
[224]	rat	30 min / 120 min	con vs isoflurane	TTC	↓	<0.05	1.00
[224]	rat	30 min / 120 min	isoflurane vs nVEGF	TTC	↑	<0.05	1.00
[224]	rat	30 min / 120 min	isoflurane vs isoflurane +nVEGF	TTC	↑	<0.05	0.94
[224]	rat	30 min / 120 min	isoflurane vs IgG	TTC	↑	<0.05	1.00
[224]	rat	30 min / 120 min	nVEGF+ISO vs IgG+isoflurane	TTC	↓	<0.05	0.64
[224]	rat	30 min / 120 min	con vs IgG+isoflurane	TTC	↓	<0.05	0.99
[224]	rat	30 min / 120 min	IgG vs IgG+Isoflurane	TTC	↓	<0.05	0.94
[225]	rat	45 min / 24 h	con vs H/R-Exo	TTC	↓	<0.05	1.00
[225]	rat	45 min / 24 h	con vs H/R-p-Exo	TTC	↓	<0.01	1.00
[225]	rat	45 min / 24 h	H/R-Exo vs H/R-p-Exo	TTC	↓	<0.01	1.00
[226]	mouse	perm / 14 d	con vs Rat IgG	Histo	→	n.s.	0.12
[226]	mouse	perm / 14 d	con vs CAG/CATZ/Rae-1e mice	Histo	↓	<0.05	0.89
[227]	rabbit	30 min / 180 min	con vs Bio A	TTC	↓	<0.01	1.00
[227]	rabbit	30 min / 180 min	con vs Bio B	TTC	↓	<0.01	0.98
[227]	rabbit	30 min / 180 min	con vs MLS2776	TTC	↓	<0.001	1.00
[227]	rabbit	30 min / 180 min	con vs MLS2777	TTC	↓	<0.001	1.00
[227]	rabbit	30 min / 180 min	con vs MLS2778	TTC	↓	<0.001	1.00
[227]	rabbit	30 min / 180 min	con vs MLS2779	TTC	↓	<0.001	1.00
[227]	rabbit	30 min / 120 min	con vs MLS2776	TTC	↓	<0.001	1.00
[227]	rabbit	30 min / 120 min	con vs MLS2778	TTC	↓	<0.001	1.00
[227]	mouse	30 min / 120 min	con vs MLS2776+CsA	TTC	↓	<0.0001	1.00
[227]	mouse	30 min / 120 min	con vs MLS2778+CsA	TTC	↓	<0.0001	1.00
[227]	mouse	30 min / 120 min	con vs CsA	TTC	↓	<0.0001	1.00
[227]	mouse	30 min / 120 min	CsA vs MLS2776	TTC	↓	<0.001	1.00
[227]	mouse	30 min / 120 min	CsA vs MLS2778	TTC	↓	<0.001	1.00
[227]	mouse	30 min / 120 min	CsA vs MLS2776+CsA	TTC	↓	<0.0001	1.00
[227]	mouse	30 min / 120 min	CsA vs MLS2778+CsA	TTC	↓	<0.0001	1.00
[228]	mouse	perm / 28 d	DMSO vs JZL184	TTC	↑	<0.05	0.96
[229]	mouse	perm / 14 d	con vs Sp-8-pCPT	Histo	↓	<0.05	0.79
[230]	mouse	30 min / 24 h	FITC-NP vs PIO-NP 0.1 mg/kg	TTC	↓	<0.05	0.87
[230]	mouse	30 min / 24 h	FITC-NP vs PIO-NP 0.3 mg/kg	TTC	↓	<0.01	0.99
[230]	mouse	30 min / 24 h	FITC-NP vs PIO-NP 1 mg/kg	TTC	↓	<0.01	1.00
[230]	mouse	30 min / 24 h	FITC-NP vs PIO-NP 3 mg/kg	TTC	↓	<0.01	1.00
[230]	pig	60 min / 24 h	saline vs PIO-NP	TTC	↓	<0.005	1.00
[230]	pig	60 min / 24 h	FITC-NP vs PIO-NP	TTC	↓	<0.005	0.94
[231]	mouse	25 min / 120 min	con vs empagliflozin (with insulin)	TTC	→	n.s.	0.07
[231]	mouse	25 min / 120 min	con vs cariporide (with insulin)	TTC	→	n.s.	0.24
[231]	mouse	25 min / 120 min	con vs empagliflozin (w.o. insulin)	TTC	→	n.s.	0.06
[231]	mouse	25 min / 120 min	con vs cariporide (w.o. insulin)	TTC	↓	<0.05	0.73
[232]	mouse	15 min / 30 min	WT vs SD2814D	TTC	→	n.s.	0.71
[232]	mouse	15 min / 30 min	WT vs PLNKO	TTC	↑	<0.05	0.98
[232]	mouse	15 min / 30 min	WT vs SDKO	TTC	↑	<0.05	1.00
[232]	mouse	15 min / 30 min	SD2814D vs SDKO	TTC	↑	<0.05	1.00
[232]	mouse	15 min / 30 min	PLNKO vs SDKO	TTC	↑	<0.05	0.95
[233]	mouse	perm / 7 d	WT vs Hspb1 -/-	TTC	↑	<0.01	1.00
[234]	mouse	60 min / 1 wk	WT vs Ldlr-E06-scFv OE	TTC	↓	0.023	0.95
[235]	mouse	45 min / 3 d	PBS vs MSC-Exo	TTC	↓	<0.0001	0.84
[235]	mouse	45 min / 3 d	PBS +CL2MDP vs MSC-Exo	TTC	↓	<0.0001	1.00
[235]	mouse	45 min / 3 d	PBS +CL2MDP vs MSC-Exo+CL2MDP	TTC	↓	<0.05	0.48
[235]	mouse	45 min / 3 d	MSC-Exo vs MSC-Exo+CL2MDP	TTC	↑	<0.001	0.70
[236]	mouse	40 min / 60 min	con vs Tat-DAXXp-scramble	TTC	→	n.s.	0.08
[236]	mouse	40 min / 60 min	con vs Tat-DAXXp	TTC	↓	<0.01	0.99
[236]	mouse	40 min / 60 min	Tat-DAXXp-scaramble vs Tat-DAXXp	TTC	↓	<0.05	0.50
[236]	mouse	40 min / 60 min	Tat 1 mg vs Tat-DAXXp 1 mg	TTC	↓	<0.0001	0.62
[236]	mouse	40 min / 60 min	Tat 1 mg vs Tat-DAXXp 10 mg	TTC	↓	<0.01	0.48
[236]	mouse	40 min / 60 min	Tat-DAXXp-scramble 1 mg vs TD 1 mg	TTC	↓	<0.01	0.57
[236]	mouse	40 min / 24 h	Tat 1 mg vs Tat-DAXXp 1 mg	TTC	↓	<0.01	0.49
[236]	mouse	40 min / 24 h	Tat 1 mg vs Tat-DAXXp 10 mg	TTC	↓	<0.001	0.55
[236]	mouse	40 min / 24 h	Tat-DAXXp scramble 1 mg vs Tat-DAXXp 1 mg	TTC	↓	<0.05	0.67
[237]	mouse	40 min / 24 h	WT vs TXNIP KO	TTC	↓	<0.01	0.68
[237]	mouse	40 min / 24 h	WT vs TXNIP OE	TTC	↑	<0.01	0.70
[238]	rat	perm / 28 d	con vs MSC-Exo	Histo	↓	<0.001	0.98
[238]	rat	perm / 28 d	con vs MSC-ATV-Exo	Histo	↓	<0.0001	1.00
[238]	rat	perm / 28 d	MSC-Exo vs MSC-ATV-Exo	Histo	↓	<0.01	0.96
[238]	rat	perm / 28 d	con vs MSC-ATV-Exo	Histo	↓	<0.0001	1.00
[238]	rat	perm / 28 d	MSC-ATV-Exo vs MSC-ATV(Si)-Exo	Histo	↑	<0.01	0.96
[238]	rat	perm / 28 d	con vs MSC-(H19)-Exo	Histo	↓	<0.001	0.97
[239]	mouse	perm / 28 d	con vs WT-hiSPC-CM	Histo	↓	<0.05	0.88
[239]	mouse	perm / 28 d	con vs CDH2-hiSPC-CM	Histo	↓	<0.05	1.00
[239]	mouse	perm / 28 d	WT-hiSPC-CM vs CDH2-hiSPC-CM	Histo	↓	<0.05	1.00
[240]	rat	perm / 5 wk	medium MI vs large MI	Histo	↑	<0.05	0.85
[240]	rat	perm / 5 wk	medium MI+PBI vs large MI+PBI	Histo	↑	<0.05	1.00
[241]	mouse	30 min / 60 min	con vs rapamycin (c57)	TTC	↓	<0.001	1.00
[241]	mouse	30 min / 60 min	con vs rapamycin (db/db)	TTC	↓	<0.001	1.00
[241]	mouse	30 min / 60 min	con (c57) vs con (db/db)	TTC	↑	<0.001	0.79
[242]	mouse	perm / 30 d	MI vs MI+CPCs	Histo	↓	0.0096	0.95
[243]	mouse	60 min / 240 min	veh vs PVS	TTC	↓	<0.0001	0.44
[244]	mouse	20 min / 90 min	WT vs CypD KI C202s	TTC	↓	<0.001	0.94
[244]	mouse	20 min / 90 min	WT vs CypD KI C202s (DMSO)	TTC	↓	<0.001	1.00
[244]	mouse	20 min / 90 min	WT vs WT+CsA	TTC	↓	<0.001	1.00
[244]	mouse	20 min / 90 min	WT vs CypD KI C202s (CsA)	TTC	→	n.s.	0.35
[245]	rat	perm / 28 d	con vs B8B2+ CSCs	Histo	↓	<0.05	0.70
[246]	mouse	perm / 28 d	WT vs S1PR1 KO	Histo	↑	<0.05	1.00
[247]	mouse	perm / 28 d	con vs febuxostat	Histo	→	n.s.	0.07
[248]	rat	perm / 6 wk	con vs ANG1-hiPSC-aCM	Histo	↓	0.029	0.91
[249]	mouse	30 min / 24 h	WT vs SETD KO	TTC	↓	0.0039	0.85

[250]	mouse	perm / 24 h	WT vs GRK5 OE	TTC	→	n.s.	0.14	
[251]	mouse	45 min / 24 h	con vs selectin-targeting glycocalyx mimetic	TTC	↓	<0.05	0.63	
[252]	mouse	perm / 21 d	WT vs FUCCI R26Rp-mice	Histo	→	n.s.	0.14	
[253]	mouse	20 min / 90 min	WT vs Ogfod1 KO	TTC	↓	0.0001	1.00	
[253]	mouse	40 min / 24 h	WT vs Ogfod1 KO	TTC	↓	0.028	0.90	
[253]	mouse	20 min / 90 min	WT vs WT+carnosine	TTC	↓	<0.0001	0.83	
[254]	rat	30 min / 90 min	con vs CAA0225	TTC	↓	<0.05	1.00	
[254]	mouse	45 min / 2 wk	con vs CAA0225	TTC	↓	<0.05	0.87	
[254]	mouse	30 min / 120 min	con vs CAA0225	TTC	↓	<0.05	0.52	
[255]	mouse	perm / 21 d	WT vs FGF10 +/-	Histo	↑	<0.001	0.71	
[255]	mouse	perm / 5d	WT vs FGF10 +/-	Histo	↑	<0.01	0.51	
[255]	mouse	perm / 21 d	con vs DOX	Histo	↓	<0.05	0.29	
[256]	mouse	60 min / 120 min	con vs hydralacine	TTC	↓	0.0003	1.00	
[256]	mouse	60 min / 120 min	con vs hydralacine	TTC	↓	0.0083	0.89	
[257]	mouse	perm / 4 wk	WT vs Col1a2Cre(ER) OE	Histo	↓	0.0103	0.91	
[257]	mouse	perm / 4 wk	R26 Yap5SA vs Col1a2 cre(ER) OE	Histo	↑	0.0482	0.64	
[258]	rat	45 min / 120 min	con vs mCBS	TTC	↓	<0.05	0.30	
[258]	rat	45 min / 120 min	con vs IPC	TTC	↓	<0.001	0.99	
[258]	rat	30 min / 120 min	veh vs histones 20ug	TTC	↑	<0.05	0.98	
[258]	rat	30 min / 120 min	histones 10ug vs histones 20ug	TTC	↑	<0,01	1.00	
[258]	rat	30 min / 120 min	veh vs HIPe	TTC	↓	<0.01	0.84	
[259]	mouse	perm / 48 h	PBS vs IL-5	TTC	↓	<0.05	0.82	
[259]	mouse	perm / 28 d	PBS vs IL-5	Histo	↓	<0.05	0.94	
[259]	mouse	perm / 28 d	PBS vs PBS +TRFK5	Histo	↑	<0.05	0.96	
[259]	mouse	perm / 28 d	PBS vs IL5+TRFK5	Histo	↑	<0.05	0.87	
[259]	mouse	perm / 28 d	IL5 vs TRFK5+PBS	Histo	↑	<0.05	1.00	
[259]	mouse	perm / 28 d	IL5 vs TRFK5+IL5	Histo	↑	<0.05	1.00	
[260]	mouse	perm / 24 h	con vs miR126 antagomir	TTC	↓	<0.01	0.43	
[261]	pig	45 min / 1 wk	normothermia vs hypothermia	MRI	→	n.s.	0.11	
[262]	mouse	perm / 24 wk	con vs ferric carboxymaltose	Histo	→	n.s.	0.14	
[263]	mouse	0	WT vs 146a-5p -/-	TTC	↑	<0.001	1.00	
[263]	mouse	perm / 1 wk	adult vs aging	Histo	↑	<0.01	0.99	
[264]	rat	perm / 21 d	MI vs early RvD1	Histo	↓	0.009	1.00	
[265]	pig	60 min / 180 min	pla vs IPC (female)	TTC	↓	0.0003	0.91	
[265]	pig	60 min / 180 min	pla vs IPC (castrated male)	TTC	↓	0.0179	0.54	
[265]	pig	60 min / 180 min	pla vs IPC (male)	TTC	↓	<0.0001	0.95	
[266]	mouse	perm / 8 wk	WT vs clock D19/D19	Histo	↓	<0.05	0.72	②
[266]	mouse	perm / 8 wk	cre-WT vs Sm-Bmal KO	Histo	↓	<0.05	0.99	②
[267]	mouse	30 min / 24 h	WT vs p55γ OE	TTC	↓	0.0003	0.99	②
[268]	mouse	perm / 28 d	WT vs ILC2s deficiency	Histo	→	n.s.	0.11	
[269]	mouse	perm / 28 d	con vs 4TTC-hCMP	Histo	↓	<0.05	0.98	
[269]	mouse	perm / 28 d	3TTC-hCMP vs 4TTC-hCMP>	Histo	↓	<0.05	0.99	
[270]	mouse	30 min / 24 h	WT vs Panx -/-	TTC	→	n.s.	0.48	
[270]	mouse	30 min / 60 min	con vs IPC	TTC	↓	<0.05	0.55	
[271]	rat	30 min / 120 min	con vs IPC (Budapest study group)	TTC	↓	<0.05	1.00	
[271]	rat	30 min / 120 min	IPC vs RICuni (Budapest study group)	TTC	↑	<0.05	1.00	
[271]	rat	30 min / 120 min	IPC vs RICbi (Budapest study group)	TTC	↑	<0.05	1.00	
[271]	rat	30 min / 120 min	con vs IPC (Szeged study group)	TTC	↓	<0.05	0.96	
[271]	rat	30 min / 120 min	IPC vs RICbi (Szeged study group)	TTC	↑	<0.05	0.88	
[272]	rat	perm / 28 d	veh vs galectin-3	Histo	↓	<0.05	0.76	①
[272]	mouse	perm / 56 d	veh vs galectin-3	Histo	→	n.s.	0.81	①
[272]	mouse	perm / 56 d	losartan vs galectin-3	Histo	→	n.s.	0.74	①
[273]	mouse	60 min / 3 d	con vs mixture of antibiotics	TTC	↓	<0.001	1.00	
[273]	mouse	60 min / 3 d	con vs GLP-2	TTC	↓	<0.0001	1.00	

Circulation Research

ref.	species	I/R timing	intervention	method	effect	p-value	power	note
[274]	mouse	50 min / 24 h	WT vs CD73-/-	MRI	→	n.s.	0.12	
[275]	mouse	perm / 24 h	Saline vs CDC	TTC	→	n.s.	0.20	
[275]	mouse	perm / 24 h	Saline vs CBSC	TTC	→	n.s.	0.07	
[275]	mouse	perm / 6 wk	Saline vs CDC	Histo	↓	<0.05	0.89	
[275]	mouse	perm / 6 wk	Saline vs CBSC	Histo	↓	<0.01	1.00	
[276]	sheep	90 min / 8 wk	con vs all treated (salvage index)	TTC	↑	0.002	0.96	
[276]	sheep	90 min / 8 wk	con vs all treated	TTC	↓	<0.001	0.99	
[277]	mouse	45 min / 24 h	nSI vs mRNA-26a	TTC	↓	<0.05	0.44	
[278]	mouse	40 min / 60 min	WT vs HAX1-1 OE	TTC	↓	<0.05	0.87	
[278]	mouse	30 min / 24 h	WT vs HAX1-1 OE	TTC	↓	<0.05	0.27	
[278]	mouse	30 min / 24 h	WT vs HAX1-1 +/-	TTC	↑	<0.05	0.98	
[279]	mouse	60 min / 24 h	WT vs CaMKIIδ KO	TTC	↓	<0.05	0.43	
[279]	mouse	60 min / 24 h	fl/fl vs c-CaMKIIδ KO	TTC	↓	<0.05	0.74	
[280]	mouse	perm / 6 wk	WT vs creatine KO	Histo	→	n.s.	0.16	
[281]	mouse	perm / 24 h	WT vs MMP-28 -/- 1d	TTC	→	n.s.	0.08	
[281]	mouse	perm / 3 d	WT vs MMP-28 -/- 3d	TTC	→	n.s.	0.27	
[281]	mouse	perm / 5 d	WT vs MMP-28 -/- 5d	TTC	→	n.s.	0.08	
[281]	mouse	perm / 7 d	WT vs MMP-28 -/- 7d	TTC	→	n.s.	0.09	
[282]	mouse	30 min / 24 h	WT vs NOX4 KO	TTC	↓	<0.05	0.69	
[282]	mouse	30 min / 24 h	WT vs NOX2 KO	TTC	↓	<0.05	0.59	
[282]	mouse	30 min / 24 h	nonOE vs vs DN-NOX OE	TTC	↑	<0.05	0.86	
[282]	mouse	30 min / 24 h	WT vs DN NOX OE	TTC	↑	<0.01	1.00	
[282]	mouse	30 min / 24 h	DN-NOX OE vs DN-NOX OE +PHD2 +/-	TTC	↓	<0.05	0.97	
[282]	mouse	30 min / 24 h	WT vs DN NOX OE	TTC	↑	<0.01	1.00	
[282]	mouse	30 min / 24 h	DN NOX OE vs DN NOX OE PPAR2a -/-	TTC	↓	<0.05	0.97	
[283]	mouse	180 min / 0 min	con vs NOX4 KO	TTC	↑	<0.05	0.83	
[284]	mouse	perm / 24 h	disrupted MI vs normal MI	TTC	→	n.s.	0.17	
[284]	mouse	perm / 1 wk	disrupted MI vs normal MI	Histo	↑	<0.01	0.76	
[285]	mouse	perm / 24 h	con vs sorefenib	TTC	→	n.s.	0.49	
[286]	mouse	perm / 1 wk	con vs gene transfer of activated Notch1	Histo	→	n.s.	0.23	
[286]	mouse	perm / 1 wk	con vs gene transfer of activated sJ1	Histo	→	n.s.	0.12	

[286]	mouse	perm / 60 d	con vs gene transfer of activated Notch1	Histo	→	n.s.	0.19
[286]	mouse	perm / 60 d	con vs gene transfer of activated sJ1	Histo	→	n.s.	0.06
[287]	mouse	perm / 5 wk	con vs YAP OE	Histo	↓	0.018	0.84
[288]	mouse	perm / 24 h	WT vs pla 2R -/-	TTC	→	n.s.	0.24
[289]	mouse	perm / 24 h	con vs TGFβ receptor–KO	TTC	→	n.s.	0.62
[290]	mouse	30 min / 24 h	con vs RIPC	TTC	↓	<0.001	1.00
[290]	mouse	30 min / 24 h	con vs RIPC+NO scavenging	TTC	→	n.s.	0.08
[290]	mouse	30 min / 60 min	con vs RIPC	TTC	↓	<0.05	0.68
[290]	mouse	30 min / 60 min	con vs RIPC wo. RH+Exogen NO	TTC	↓	<0.05	0.90
[291]	mouse	perm / 1 wk	con vs Foxp3DTR Treg cell ablation	Histo	↑	<0.05	0.72
[292]	mouse	perm / 7 d	PBS vs diphtheria toxin	Histo	→	n.s.	0.12
[292]	mouse	perm / 7 d	PBS vs diphtheria toxin+IL10 KO	Histo	→	n.s.	0.10
[293]	mouse	20 min / 120 min	con vs IPC	TTC	↓	<0.05	1.00
[293]	mouse	20 min / 120 min	con+wortmannin vs IPC+wortmannin	TTC	→	n.s.	0.42
[293]	mouse	20 min / 120 min	con+KU63794 vs IPC+KU63794	TTC	→	n.s.	0.35
[293]	mouse	20 min / 120 min	con vs IPC+pp242	TTC	↓	<0.05	0.14
[293]	mouse	20 min / 120 min	con vs IPC+rapamycin	TTC	↓	<0.05	1.00
[294]	mouse	perm / 21 d	WT vs human TIMP-4 OE	Histo	→	n.s.	0.05
[295]	mouse	perm / 14 d	veh vs saphenous vein–derived pericytes (SVP)+cardiac stem cells (CSC)	Histo	↓	<0.05	0.93
[296]	mouse	30 min / 4 h	WT vs GSTP-null	TTC	↑	<0.05	0.86
[297]	mouse	30 min / 24 h	con vs Drp1-C KO	TTC	↑	<0.05	0.97
[298]	mouse	perm / 5-6 wk	WT vs protein 1 Cre-tandem dimer Tomato OE	Histo	↓	0.046	0.67
[299]	mouse	30 min / 48 h	con vs IPC in mice	TTC	↓	<0.0001	1.00
[299]	rabbit	45 min / 72 h	con vs IPC in rabbits	TTC	↓	<0.0001	1.00
[299]	pig	60 min / 72 h	con vs IPC in pigs	TTC	↓	0.0121	0.84
[300]	mouse	perm / 8 wk	PBS vs mES Ex-CPC	Histo	↓	<0.01	0.76
[300]	mouse	perm / 8 wk	PBS vs mEF EX-CPC	Histo	→	n.s.	0.11
[300]	mouse	perm / 8 wk	MEF Ex-CPC vs mES Ex-CPC	Histo	↓	<0.01	0.86
[301]	mouse	perm / 1 wk	con vs MIPC S	MRI	↓	<0.01	1.00
[301]	mouse	perm / 2 wk	con vs MIPC S	MRI	↓	<0.01	1.00
[301]	mouse	perm / 4 wk	con vs MIPC S	MRI	↓	<0.01	1.00
[301]	mouse	perm / 1/4 wk	MIPC S 1 wk vs MIPC S 4 wk	Histo	↓	<0.05	0.99
[302]	rat	60 min / 24 h	con vs eng. heart muscle	MRI	→	n.s.	0.14
[302]	rat	60 min / 28 d	con vs eng. heart muscle	MRI	→	n.s.	0.06
[302]	rat	60 min / 24 h	con vs eng. heart muscle (irradiated)	MRI	→	n.s.	0.07
[302]	rat	60 min / 28 d	con vs eng. heart muscle (irradiated)	MRI	→	n.s.	0.16
[303]	pig	60 min / 180 min	pla vs RIPC	TTC	↓	<0.05	1.00
[303]	pig	60 min / 180 min	pla vs RIPC+RISK-BL	TTC	↓	<0.05	1.00
[303]	pig	60 min / 180 min	pla vs RIPC+SAFE-BL	TTC	→	n.s.	0.11
[303]	pig	60 min / 180 min	RIPC vs RIPC +SAFE-BL	TTC	↑	<0.05	0.97
[303]	rat	30 min / 120 min	pPLA vs pRIPC	TTC	↓	<0.05	1.00
[303]	rat	30 min / 120 min	pPLA +RISK-BL vs pRIPC	TTC	↓	<0.05	1.00
[303]	rat	30 min / 120 min	pPLA +SAFE-BL vs pRIPC	TTC	↓	<0.05	1.00
[303]	rat	30 min / 120 min	pRIPC vs pRIPC +RISK-BL	TTC	↑	<0.05	1.00
[303]	rat	30 min / 120 min	pRIPC vs pRIPC +SAFE-BL	TTC	↑	<0.05	1.00
[304]	mouse	perm / 3 d	WT vs Osteoglycin null	Histo	→	n.s.	0.06
[305]	mouse	perm / 3-7 d	IS day 3 vs IS day 7 (neonates)	Histo	↓	<0.05	1.00
[305]	mouse	perm / 3-7 d	IS day 3 vs IS day 7 (adult)	Histo	→	n.s.	0.28
[306]	mouse	perm / 7 d	WT vs FOXO4 -/-	Histo	↓	<0.05	0.89
[307]	mouse	perm / 48 h	con vs Gca KO	TTC	↓	<0.05	0.88
[308]	rat	perm / 24 h	con vs renal denervation (SHR rat)	TTC	↓	<0.01	0.94
[308]	rat	perm / 24 h	con vs renal denervation (WKY rat)	TTC	→	n.s.	0.31
[309]	rat	90 min / 12 mo	veh vs CPC	Histo	↓	<0.01	0.90
[310]	mouse	30 min / 72 h	WT vs GRK2 KO	TTC	↓	<0.05	0.80
[311]	mouse	30 min / 24 h	con vs γ2-AMPK cKO	TTC	↑	<0.05	1.00
[311]	mouse	30 min / 24 h	WT vs γ2-AMPK OE	TTC	↓	<0.05	1.00
[311]	mouse	30 min / 24 h	WT veh vs WT+TUDCA	TTC	↓	<0.05	1.00
[311]	mouse	30 min / 24 h	WT veh vs γ2-AMPK OE+TUDCA	TTC	↓	<0.05	1.00
[312]	mouse	45 min / 4 h	MerTK+/+ vs MerTK-/-	TTC	↓	<0.01	*
[312]	mouse	45 min / 1 wk	MerTK+/+ vs CR/CR	TTC	↓	<0.01	*
[312]	mouse	45 min / 1 wk	MerTK++ vs MerTK-- in LysM-Cre	TTC	↑	<0.05	*
[313]	mouse	45 min / 24 h	WT vs MitEpac1 -/-	TTC	↓	<0.01	0.91
[314]	pig	40 min / 120 min	con vs PoCo	MRI	↑	<0.05	0.68
[314]	pig	40 min / 120 min	con vs IPC	MRI	↓	<0.05	1.00
[314]	pig	20 min / 120 min	40 min I vs 20 min I	MRI	↓	<0.05	1.00
[314]	pig	40 min / 24 h	con vs PoCo	MRI	→	n.s.	0.36
[314]	pig	40 min / 24 h	con vs IPC	MRI	↓	<0.05	0.72
[314]	pig	20 min / 24 h	40 min I vs 20 min I	MRI	↓	<0.05	1.00
[314]	pig	40 min / 4 d	con vs PoCo	MRI	↑	<0.05	0.43
[314]	pig	40 min / 4 d	con vs IPC	MRI	↓	<0.05	1.00
[314]	pig	20 min / 4 d	40 min I vs 20 min I	MRI	↓	<0.05	1.00
[314]	pig	40 min / 7 d	con vs PoCo	MRI	↑	<0.05	0.60
[314]	pig	40 min / 7 d	con vs IPC	MRI	↓	<0.05	1.00
[314]	pig	20 min / 7 d	40 min I vs 20 min I	MRI	↓	<0.05	1.00
[315]	mouse	perm / 4 wk	MI vs MI+hCMP	Histo	↓	<0.05	0.79
[315]	mouse	perm / 4 wk	MI+Scaffold vs MI+hCMP	Histo	↓	<0.05	0.78
[316]	mouse	perm / 8 wk	con vs miR199a-3p	Histo	↓	<0.01	0.69
[316]	mouse	perm / 8 wk	con vs miR590-3p	Histo	↓	<0.01	0.95
[317]	mouse	45 min / 20 d	no MSCs vs MSCs	TTC	→	n.s.	0.11
[318]	mouse	perm / 15 d	con vs syn MSC	Histo	↓	<0.05	0.84
[318]	mouse	perm / 15 d	con vs MSC	Histo	↓	<0.05	0.96
[319]	pig	90 min / 72 h	veh vs CBSCs (72h)	TTC	→	n.s.	0.07
[319]	pig	90 min / 3 mo	veh vs CBSCs (3wk)	TTC	↓	<0.05	0.82
[320]	mouse	perm / 28 d	con vs ANG-II	Histo	↑	<0.01	1.00
[321]	mouse	perm / 2/4 wk	con 2wk vs con 4wk	MRI	↑	<0.05	0.27
[321]	mouse	perm / 2/4 wk	hCMs 2wk vs hCMs 4wk	MRI	↓	<0.05	0.22
[321]	mouse	perm / 4 wk	con 4wk vs hCMs 4wk	MRI	↓	<0.05	0.72
[321]	mouse	perm / 4 wk	hESCs 4wk vs hCMs 4wk	MRI	↓	<0.05	0.54

[321]	mouse	perm / 2/4 wk	con 4wk vs iCMs 4 wk	MRI	↓	<0.05	0.35	
[321]	mouse	perm / 4 wk	IPSCs 4wk vs iCMs 4 wk	MRI	↓	<0.09	0.25	
[322]	mouse	perm / 48 h	WT vs D2 KO	TTC	↓	<0.001	0.99	
[322]	mouse	perm / 14 d	WT vs D2 KO	Histo	↓	<0.001	0.95	
[322]	mouse	perm / 28 d	WT vs D2 KO	Histo	→	n.s.	0.09	
[323]	mouse	20 min / 120 min	GSNO-R +/+ vs GSNO-R -/- in male	TTC	↓	<0.05	0.54	②
[323]	mouse	20 min / 120 min	GSNO-R +/+ vs GSNO-R -/- in female	TTC	↑	<0.05	0.70	②
[323]	mouse	25 min / 24 h	GSNO-R +/+ vs GSNO-R -/- in male	TTC	↓	<0.05	0.71	②
[323]	mouse	25 min / 24 h	GSNO-R +/+ vs GSNO-R -/- in female	TTC	↑	<0.05	0.80	②
[323]	mouse	20 min / 120 min	GSNO-R +/+ vs GSNO-R -/- in male	TTC	↓	<0.05	0.91	②
[323]	mouse	20 min / 120 min	GSNO-R +/+ vs GSNO-R +/+ in male+Alda-1	TTC	↓	<0.05	0.64	②
[323]	mouse	20 min / 120 min	GSNO-R +/+ vs GSNO-R -/- in male+Alda-1	TTC	↓	<0.05	0.83	②
[323]	mouse	20 min / 120 min	GSNO-R +/+ vs GSNO-R -/- in female	TTC	↑	<0.01	0.92	②
[323]	mouse	20 min / 120 min	GSNO-R +/+ vs GSNO-R -/- in female +Alda-1	TTC	↓	<0.05	0.18	②
[324]	pig	60 min / 180 min	con vs RIPC	TTC	↓	<0.001	1.00	
[325]	pig	60 min / 180 min	con vs RIPC	TTC	↓	<0.001	1.00	
[325]	pig	60 min / 180 min	vagotomy vs RIPC	TTC	↓	<0.001	1.00	
[325]	pig	60 min / 180 min	SPLX vs RIPC	TTC	↓	<0.001	0.85	
[325]	pig	60 min / 180 min	vagotomy vs vagotomy+RIPC	TTC	↓	<0.001	0.91	
[325]	pig	60 min / 180 min	RIPC vs vagotomy+RIPC	TTC	↑	<0.001	0.75	
[325]	pig	60 min / 180 min	RIPC vs SPLX+RIPC	TTC	↑	<0.001	0.73	
[325]	pig	60 min / 180 min	RIPC vs splenic denervation+RIPC	TTC	↑	<0.001	0.96	
[325]	rat	30 min / 120 min	con vs RIPC	TTC	↓	<0.05	1.00	
[325]	rat	30 min / 120 min	RIPC vs vagotomy+RIPC	TTC	↑	<0.05	1.00	
[325]	rat	30 min / 120 min	RIPC vs SPLX+RIPC	TTC	↑	<0.05	1.00	
[325]	rat	30 min / 120 min	RIPC vs splenic denervation+RIPC	TTC	↑	<0.05	1.00	
[325]	rat	30 min / 120 min	ppla vs pRIPC	TTC	↓	<0.05	1.00	
[325]	rat	30 min / 120 min	ppla+p(vagotomy vs RIPC)	TTC	↓	<0.05	0.98	
[325]	rat	30 min / 120 min	pSPLX vs pRIPC	TTC	↓	<0.001	0.95	
[325]	rat	30 min / 120 min	pRIPC vs pRIPC+vagotomy	TTC	↑	<0.05	1.00	
[325]	rat	30 min / 120 min	pRIPC vs p(RIPC+SPLX)	TTC	↑	<0.05	0.83	
[325]	rat	30 min / 120 min	pRIPC vs p(splenic denervation)	TTC	↑	<0.001	0.98	
[325]	rat	30 min / 120 min	saline vs carbachol	TTC	↓	<0.05	1.00	
[325]	rat	30 min / 120 min	TC vs saline	TTC	↑	<0.05	1.00	
[325]	rat	30 min / 120 min	TC vs 1pM carbachol	TTC	↑	<0.05	1.00	
[325]	rat	30 min / 120 min	TC vs 10pM carbachol	TTC	↑	<0.05	1.00	
[325]	rat	30 min / 120 min	TC vs 20pM carbachol	TTC	↑	<0.05	1.00	
[325]	rat	30 min / 120 min	TC vs 100pM carbachol	TTC	↑	<0.05	1.00	
[325]	rat	30 min / 120 min	TC vs 1000pM carbachol	TTC	↑	<0.05	1.00	
[325]	rat	30 min / 120 min	TC vs 100000pM carbachol	TTC	↑	<0.05	1.00	
[325]	rat	30 min / 120 min	saline vs 100pM carbachol	TTC	↓	<0.05	1.00	
[325]	rat	30 min / 120 min	saline vs 1000pM carbachol	TTC	↓	<0.05	1.00	
[325]	rat	30 min / 120 min	saline vs 100000pM carbachol	TTC	↓	<0.05	1.00	
[325]	rat	30 min / 120 min	1pM carbachol vs 100pM carbachol	TTC	↓	<0.05	1.00	
[325]	rat	30 min / 120 min	1pM carbachol vs 100pM carbachol	TTC	↓	<0.05	1.00	
[325]	rat	30 min / 120 min	1pM carbachol vs 100pM carbachol	TTC	↓	<0.05	1.00	
[325]	rat	30 min / 120 min	10pM carbachol vs 100pM carbachol	TTC	↓	<0.05	0.99	
[325]	rat	30 min / 120 min	10pM carbachol vs 1000pM carbachol	TTC	↓	<0.05	0.98	
[325]	rat	30 min / 120 min	10pM carbachol vs 100000pM carbachol	TTC	↓	<0.05	1.00	
[325]	rat	30 min / 120 min	20pM carbachol vs 100pM carbachol	TTC	↓	<0.05	1.00	
[325]	rat	30 min / 120 min	20pM carbachol vs 1000pM carbachol	TTC	↓	<0.05	0.99	
[325]	rat	30 min / 120 min	20pM carbachol vs 100000pM carbachol	TTC	↓	<0.05	1.00	
[326]	mouse	perm / 28 d	WT vs OSTN (osteocrin) OE	Histo	↓	<0.05	0.70	
[327]	mouse	60 min / 24 h	WT vs myonectin KO	TTC	↑	<0.01	0.95	
[327]	mouse	60 min / 24 h	WT sedentary vs WT exercise	TTC	↓	<0.05	0.99	
[327]	mouse	60 min / 24 h	myonektin KO sedentary vs moynektin KO exercise	TTC	→	n.s.	0.30	
[327]	mouse	60 min / 24 h	WT vs myonectin OE	TTC	↓	<0.01	0.89	
[328]	pig	60 min / 1 wk	con vs metformin	TTC	→	n.s.	0.18	
[329]	mouse	perm / 28 d	con vs Exo	Histo	↓	<0.05	0.86	
[329]	mouse	perm / 28 d	Exo-scramble vs Exo anti miR125b	Histo	↑	<0.05	0.54	
[330]	mouse	perm / 28 d	WT vs E2F1	Histo	↓	<0.01	0.94	②
[331]	rabbit	30 min / 2 wk	veh vs Muse	Histo	↓	<0.001	1.00	②
[331]	rabbit	30 min / 2 wk	veh vs non-Muse	Histo	↓	<0.001	0.90	②
[331]	rabbit	30 min / 2 wk	veh vs MSC	Histo	↓	<0.001	0.95	②
[331]	rabbit	30 min / 2 wk	Muse vs non-Muse	Histo	↑	<0.001	0.91	②
[331]	rabbit	30 min / 2 wk	Muse vs MSC	Histo	↑	<0.001	0.78	②
[331]	rabbit	30 min / 2 mo	veh vs Muse	Histo	↓	<0.001	1.00	②
[331]	rabbit	30 min / 2 mo	veh vs non-Muse	Histo	↓	<0.05	0.41	②
[331]	rabbit	30 min / 2 mo	veh vs MSC	Histo	↓	<0.05	0.65	②
[331]	rabbit	30 min / 2 mo	Muse vs non-Muse	Histo	↑	<0.001	1.00	②
[331]	rabbit	30 min / 2 mo	Muse vs MSC	Histo	↑	<0.01	1.00	②
[332]	mouse	perm / 28 d	PBS vs CCND2 OE	Histo	↓	<0.05	0.71	
[333]	mouse	perm / 8 wk	ctrl vs transient introduction of miR-294	Histo	↓	<0.01	0.99	
[334]	rat	30 min / 24 h	veh vs Exercise-Exo	TTC	↓	<0.01	0.99	
[334]	rat	30 min / 24 h	veh vs sedentary-Exo	TTC	→	n.s.	0.12	
[334]	rat	30 min / 24 h	sedentary - neg con vs exercise neg con	TTC	↓	<0.01	1.00	
[334]	rat	30 min / 24 h	exercise neg con vs exercise anti-342	TTC	↑	<0.05	0.92	
[334]	rat	30 min / 24 h	agomiR neg cog vs mir-342 agomir	TTC	↓	<0.05	1.00	
[335]	mouse	perm / 14 d	WT vs anti-IL35	Histo	→	n.s.	0.11	
[336]	mouse	perm / 28 d	WT vs sulf1 -/-	Histo	↑	<0.05	0.77	
[336]	mouse	perm / 28 d	WT vs sulf2 -/-	Histo	↑	<0.05	0.85	
[336]	mouse	perm / 28 d	con vs surfen	Histo	↑	<0.001	1.00	
[337]	mouse	60 min / 3 wk	con vs HAS2 KO	Histo	↑	<0.05	0.68	②
[337]	mouse	25 min / 60 d	con vs HAS2 KO	Histo	→	n.s.	0.06	②
[338]	mouse	perm / 15-21 d	Adm +/+ vs ADM hi/hi (male)	Histo	→	n.s.	0.20	
[338]	mouse	perm / 15-21 d	Adm +/+ vs ADM hi/hi (female)	Histo	→	n.s.	0.30	
[339]	rat	30 min / 35 d	veh vs CD45-Lin-	Histo	→	n.s.	0.44	
[340]	mouse	30 min / 24 h	AD vector vs LAPTM4B -/-	TTC	↓	0.0023	0.96	

[340]	mouse	30 min / 24 h	WT vs LAPTM4B -/-	TTC	↑	0.000000085	1.00	
[340]	mouse	30 min / 24 h	con vs LABTM4B OE	TTC	↓	0.000018	1.00	
[340]	mouse	30 min / 24 h	LABTM4B OE vs BAF+LABTM4B OE	TTC	↑	0.0015	0.92	
[340]	mouse	30 min / 24 h	WT vs LABTM4B -/-	TTC	↑	0.0022	0.96	
[340]	mouse	30 min / 24 h	WT vs WT RAPA	TTC	↓	0.0277	0.93	
[340]	mouse	30 min / 24 h	LABTM4B -/- vs LABTM4B -/- +rapamycin	TTC	↓	0.0001	1.00	
[340]	mouse	30 min / 24 h	WT vs LABTM4B -/-	TTC	↑	0.000000085	0.99	
[340]	mouse	30 min / 24 h	WT vs WT siRaptor	TTC	↓	0.003	0.97	
[340]	mouse	30 min / 24 h	LABTM4B -/- vs LABTM4B -/- +siRaptor	TTC	↓	0.00000051	1.00	
[340]	mouse	30 min / 24 h	vector WT vs LABTM4B -/-	TTC	↑	0.0005	0.97	
[340]	mouse	30 min / 24 h	vector WT vs WT TFEBr OE	TTC	↓	0.000024	1.00	
[340]	mouse	30 min / 24 h	vector LABTM4B -/- vs LABTM4B -/- TFEB OE	TTC	↓	0.000026	1.00	
[341]	mouse	perm / 21 d	con vs ABR-238901	Histo	↑	<0.05	0.79	
[342]	mouse	perm / 8 wk	veh vs ADSC-Ncad	Histo	↓	0.0024	1.00	
[342]	mouse	perm / 8 wk	ADSC-con vs ADSC-Ncad	Histo	↓	0.0305	1.00	
[342]	mouse	perm / 8 wk	ADSC-con vs ADSC-WT-Ncad	Histo	↓	0.0005	1.00	
[342]	mouse	perm / 8 wk	ADSC-WT-Ncad vs ADSC-KD-Ncad	Histo	↑	0.0334	0.81	
[343]	mouse	perm / 30 d	PBS vs WT-EPC-Exo	Histo	↓	0.0046	0.48	
[343]	mouse	perm / 30 d	WT-EPC-Exo vs IL10 KO EPC-Exo	Histo	↑	0.0005	0.48	
[343]	mouse	perm / 30 d	PBS vs WT-EPC-Exo	Histo	↓	<0.0001	0.50	
[343]	mouse	perm / 30 d	PBS vs ILK-KD-EPC-Exo	Histo	↓	<0.0001	0.43	
[343]	mouse	perm / 30 d	IL10 KO EPC-Exo vs wt-EPC-Exo	Histo	↓	<0.0001	0.36	
[343]	mouse	perm / 30 d	IL10 KO EPC-Exo vs ILK-KD-EPC-Exo	Histo	↓	<0.001	0.30	
[344]	mouse	perm / 28 d	saline vs SNC pre	Histo	↓	<0.05	0.88	
[344]	mouse	perm / 28 d	saline vs SNC interv	Histo	↓	<0.05	1.00	
[344]	mouse	perm / 28 d	SNC interv vs SNC pre	Histo	↑	<0.05	0.99	
[345]	mouse	90 min / 24 h	con vs fIIa inh	TTC	↓	<0.01	0.99	
[345]	mouse	90 min / 24 h	con vs fXa inh	TTC	↓	<0.01	1.00	
[345]	mouse	90 min / 24 h	con vs fIIa inh	TTC	↓	<0.05	1.00	
[345]	mouse	90 min / 24 h	con vs fIIa inh+3K3A-aPC	TTC	↓	<0.01	1.00	
[345]	mouse	90 min / 24 h	fIIa inh vs fIIa inh+3K3A-aPC	TTC	↓	<0.01	1.00	
[345]	mouse	90 min / 24 h	con vs fXa inh	TTC	↓	<0.01	1.00	
[345]	mouse	90 min / 24 h	con vs fXa inh+MAPC1591	TTC	↓	<0.05	0.98	
[345]	mouse	90 min / 24 h	fXa inh vs fXa inh+MPC1609	TTC	↑	<0.01	1.00	
[345]	mouse	90 min / 24 h	fXa inh+MPC1609 vs fXa inh+MAPC1591	TTC	↓	<0.05	1.00	
[346]	mouse	30 min / 4-7 wk	WT vs miR30d OE	TTC	↓	0.0016	0.97	
[347]	mouse	perm / 28 d	Mst1/2 fl/fl vs LysMCre-Mst1/2 fl/fl	Histo	↑	0.013	0.92	
[347]	mouse	perm / 28 d	LysMCre-Mst1/2 fl/fl vs LysMCre-Mst1/2 fl/fl+CP	Histo	↓	0.013	0.99	
[347]	mouse	perm / 28 d	veh vs XMU	Histo	↑	0.0025	0.96	
[347]	mouse	perm / 28 d	XMU vs XMU+CP	Histo	↓	0.000000081	1.00	
[347]	mouse	perm / 28 d	veh vs xmu+CP	Histo	↓	0.0067	0.98	
[347]	mouse	perm / 28 d	CP vs XMU+CP	Histo	↓	0.043	0.95	
[348]	mouse	perm / 28 d	con vs MSC-Exo	Histo	↓	0.002	1.00	
[349]	mouse	30 min / 24 h	WT vs GSDMD KO	TTC	↓	0.000077	0.41	
[349]	mouse	30 min / 24 h	Myh6-cre vs GHDMD KO	TTC	↓	0.000049	0.52	
[349]	mouse	30 min / 24 h	GHDMD f/f vs GHDMD KO	TTC	↓	0.00015	0.46	
[350]	mouse	30 min / 24 h	veh vs MNF	TTC	↓	0.0025	0.83	
[351]	mouse	45 min / 24 h	veh vs TAT scratch	TTC	→	n.s.	0.28	
[351]	mouse	45 min / 24 h	veh vs TAT-Beclin	TTC	↓	0.0486	0.90	
[351]	mouse	45 min / 24 h	TAT scratch vs TAT-Beclin	TTC	↓	0.0011	0.96	
[352]	mouse	perm / 28 d	WT vs cilp1 KO	Histo	↓	<0.05	0.61	②
[352]	mouse	perm / 28 d	WT vs cilp1 OE	Histo	↑	<0.05	0.86	②
[353]	mouse	30 min / 120 min	L2HGDH +/+ vs L2HDGH +/-	TTC	↓	<0.05	0.56	①
[353]	mouse	30 min / 120 min	L2HGDH +/+ vs L2HDGH -/-	TTC	↓	<0.001	1.00	①
[353]	mouse	30 min / 60 min	L2HGDH +/+ vs L2HDGH +/-	TTC	↓	<0.001	0.83	①
[353]	mouse	30 min / 60 min	L2HGDH +/+ vs L2HDGH -/-	TTC	↓	<0.001	1.00	①
[353]	mouse	30 min / 60 min	L2HGDH +/- vs L2HDGH -/-	TTC	↓	<0.001	1.00	①
[353]	mouse	30 min / 60 min	L2HGDH +/+ vs L2HDGH +/-	TTC	↓	<0.01	1.00	①
[353]	mouse	30 min / 60 min	L2HGDH +/+ vs L2HDGH -/-	TTC	↓	<0.05	1.00	①
[354]	mouse	30 min / 24 h	HSA vs rh mg53-WT (male db/db 8 wk)	TTC	↓	0.0057	0.60	
[354]	mouse	30 min / 24 h	HSA vs rh mg53-S255A (male db/db 8 wk)	TTC	↓	0.0032	0.95	
[354]	mouse	30 min / 24 h	rh mg53-WT vs rh mg53-S255A (male db/db 8 wk)	TTC	↓	0.000012	0.68	
[354]	mouse	30 min / 24 h	HSA vs rh mg53-WT (male db/db 12 wk)	TTC	→	n.s.	0.06	
[354]	mouse	30 min / 24 h	HSA vs rh mg53-S255A (male db/db 12 wk)	TTC	↓	0.00023	0.97	
[354]	mouse	30 min / 24 h	rh mg53-WT vs rh mg53-S255A (male db/db 12 wk)	TTC	↓	0.0014	0.80	
[354]	mouse	30 min / 24 h	HSA vs rh mg53-WT (female db/db 12 wk)	TTC	→	n.s.	0.06	
[354]	mouse	30 min / 24 h	HSA vs rh mg53-S255A (female db/db 12 wk)	TTC	↓	0.023	0.70	
[354]	mouse	30 min / 24 h	rh mg53-WT vs rh mg53-S255A (female db/db 12 wk)	TTC	↓	0.00076	0.82	
[354]	mouse	30 min / 24 h	HSA vs rh mg53-WT (mg53 KO)	TTC	↓	0.0091	0.64	
[354]	mouse	30 min / 24 h	HSA vs rh mg53-S255A (mg53 KO)	TTC	↓	0.00047	1.00	
[354]	mouse	30 min / 24 h	rh mg53-WT vs rh mg53-S255A (mg53 KO)	TTC	→	n.s.	0.10	
[355]	mouse	perm / 1 wk	WT vs Arrdc4 KO	TTC	↓	0.00024	0.54	
[356]	monkey	perm / 30 d	nc vs LV-MnmFMO2	MRI	↓	0.032	0.91	
[356]	rat	perm / 30 d	nc vs LV-MnmFMO2	Histo	↓	0.019	0.91	
[357]	mouse	perm / 8 wk	WT vs LAPR2 KO	Histo	↑	0.0014	0.87	
[357]	mouse	perm / 23 d	WT vs LAPR2 KO	Histo	↑	0.00076	0.99	
[357]	mouse	perm / 3 d	WT vs LAPR2 KO	TTC	↑	<0.001	1.00	
[358]	mouse	30 min / 120 min	con vs DSM isch	TTC	→	n.s.	0.09	
[358]	mouse	30 min / 120 min	con vs DSM rep	TTC	↓	0.0035	1.00	
[358]	mouse	30 min / 120 min	veh vs CsA	TTC	↓	0.0379	1.00	
[358]	mouse	30 min / 120 min	veh vs DSM	TTC	↓	0.0132	1.00	
[358]	mouse	30 min / 120 min	veh vs CsA+DSM	TTC	↓	0.000143	1.00	
[359]	mouse	30 min / 24 h	WT vs het PDE4B	TTC	→	n.s.	0.11	
[359]	mouse	30 min / 24 h	WT vs PDB4E KO	TTC	↓	0.029	0.88	
[359]	mouse	30 min / 24 h	rcp WT / don WT vs rcp WT / don KO	TTC	↓	0.033	0.72	
[359]	mouse	30 min / 24 h	rcp WT / don WT vs rcp KO / don KO	TTC	↓	0.00000011	1.00	
[359]	mouse	30 min / 24 h	rcp KO / don WT vs rcp KO / don KO	TTC	↓	0.0013	0.87	
[359]	mouse	30 min / 24 h	rcp WT / don KO vs rcp KO / don KO	TTC	↓	0.024	0.80	

[359]	mouse	30 min / 24 h	veh vs PICL 5 mg/kg	TTC	↓	0.00074	0.71	
[359]	mouse	30 min / 24 h	veh vs PICL 10 mg/kg	TTC	↓	0.034	0.94	
[360]	mouse	45 min / 24 h	WT vs CIRCIL OE	TTC	↑	0.0000085	1.00	
[360]	mouse	45 min / 24 h	WT vs CIRCIL KO	TTC	↓	0.0000013	0.98	
[360]	mouse	45 min / 24 h	WT vs CIRCIL OE	TTC	↑	0.000051	1.00	
[360]	mouse	45 min / 24 h	CIRCIL OE+KU70 vs CIRCIL OE+vector	TTC	↑	0.0000049	1.00	
[361]	mouse	60 min / 2 wk	con vs harmine	TTC	→	n.s.	0.18	①
[361]	mouse	60 min / 2 wk	con vs DYRK1a KO	TTC	→	n.s.	0.21	①
[362]	mouse	40 min / 24 h	sedentary vs exercise	TTC	↓	0.0012	1.00	
[362]	mouse	40 min / 24 h	exercise vs exercise+BAT ablation	TTC	↑	0.014	0.99	
[362]	mouse	40 min / 24 h	shRab27a#2+exercise sham vs shRab27a#2	TTC	↓	0.0000057	1.00	
[362]	mouse	40 min / 24 h	shRab27a#2 sedentary vs scramble exercise	TTC	↑	0.028	0.96	
[362]	mouse	40 min / 24 h	shRab27a#2 sedentary vs shRab26a#3 sedentary	TTC	↑	0.018	0.95	
[362]	mouse	40 min / 24 h	veh vs BATsEVs	TTC	↓	0.000000000097	1.00	
[362]	mouse	40 min / 24 h	sedentary NC vs exercise NC	TTC	↓	0.00019	1.00	
[362]	mouse	40 min / 24 h	exercise NC vs exercise antagomiRs	TTC	↑	0.0013	1.00	
[363]	mouse	30 min / 120 min	con vs LM22A-4	TTC	↓	0.0008	1.00	
[363]	mouse	30 min / 120 min	con vs LM22A-4 (myoBDNF KO)	TTC	↓	0.0012	1.00	
[363]	mouse	30 min / 120 min	WT vs myoBDNF KO	TTC	↑	0.0024	1.00	
[363]	mouse	30 min / 120 min	LM22A-4 WT vs LM22A-4 myoBDNF KO	TTC	↑	0.0012	1.00	
[363]	mouse	30 min / 120 min	con vs BRL37344 (WT)	TTC	↓	0.02	1.00	
[363]	mouse	30 min / 120 min	WT vs myoBDNF KO	TTC	↑	0.003	1.00	
[363]	mouse	30 min / 120 min	WT vs BRL37344+myoBDNF KO	TTC	↑	0.0024	1.00	
[363]	mouse	30 min / 120 min	BRL37344 WT vs BRL37344+myoBDNF KO	TTC	↑	0.0012	1.00	
[364]	mouse	perm / 28 d	WT vs ZBP1 KO	Histo	→	n.s.	0.07	
[365]	rat	perm / 28 d	PBS vs tropoelastin	Histo	↓	0.0001	1.00	②
[366]	rat	30 min / 120 min	PBS vs GSH 10mM	TTC	↓	0.0004	0.98	
[366]	rat	30 min / 120 min	PBS vs GSSG 10mM	TTC	↓	0.00051	1.00	
[366]	rat	30 min / 120 min	PBS vs Fer-1	TTC	↓	0.000024	0.99	
[366]	rat	30 min / 120 min	PBS vs delayed Fer-1	TTC	↓	0.023	0.42	
[366]	rat	30 min / 120 min	Fer1 vs delayed Fer-1	TTC	↑	0.00052	0.72	
[366]	rat	30 min / 120 min	PBS vs MK571	TTC	↓	0.0036	0.79	
[366]	rat	30 min / 120 min	PBS vs delayed MK571	TTC	↓	0.041	0.42	
[366]	rat	30 min / 120 min	PBS vs MK571+Fer-1	TTC	↓	0.035	0.77	
[366]	rat	30 min / 120 min	MK571 vs delayed MK571	TTC	↑	0.013	0.57	
[367]	mouse	45 min / 24 h	WT vs ucp1 -/-	TTC	↑	0.03	0.73	
[367]	mouse	45 min / 24 h	WT vs BAT	TTC	↓	0.04	0.69	
[367]	mouse	45 min / 24 h	sham vs WT BAT (Bmp3-/-)	TTC	↑	0.005	0.94	
[367]	mouse	45 min / 24 h	WT BAT (Bmp3-/-) vs BMP3-/- BAT (Bmp3-/-)	TTC	↓	0.00001	1.00	
[368]	mouse	30 min / 1 wk	lox vs Pf4D	TTC	↑	0.0017	0.97	②
[368]	mouse	30 min / 1 wk	Pf4D con vs Pf4D ticagrelor	TTC	↓	0.0244	1.00	②
[368]	mouse	30 min / 4 wk	lox vs Pf4D	TTC	↓	0.0082	1.00	②
[369]	pig	60 min / 28 d	veh vs nGFP-CM SMRTs	MRI	↓	0.00194	1.00	
[369]	pig	60 min / 28 d	veh vs CCND2-CM SMRTs	MRI	↓	0.00477	1.00	
[369]	mouse	perm min / 28d	veh vs L7Ae-modRNA	Histo	↓	0.0474	1.00	
[369]	mouse	perm min / 28d	veh vs CCND2-CM SMRTs	Histo	↓	0.00000355	1.00	
[369]	mouse	perm min / 28d	L7Ae-modRNA vs LUC-CM SMRTs	Histo	↓	0.00256	1.00	
[369]	mouse	perm min / 28d	LUC-CM SMRTs vs CCND2-CM SMRTS	Histo	↓	0.00144	1.00	
[370]	mouse	perm / 7 d	FAP+/+ vs FAP-/-	Histo	↓	0.043	0.94	②
[370]	mouse	perm / 7 d	veh vs FAPi (Nppb +/+)	Histo	↓	0.013	1.00	②
[370]	mouse	perm / 7 d	veh vs FAPi (Npr1 +/+)	Histo	→	n.s.	0.89	②
[370]	mouse	perm / 7 d	FAPi vs FAPi+Nppb +/+	Histo	↑	0.0046	0.95	②
[370]	mouse	perm / 7 d	FAPi vs FAPi Npr1 +/+	Histo	↑	0.00000058	1.00	②
[371]	mouse	45 min / 24 h	WT vs ASPP1 OE	TTC	↑	0.0000052	1.00	
[371]	mouse	45 min / 24 h	WT vs ASPP1 KO	TTC	↓	0.00000013	1.00	
[371]	mouse	45 min / 24 h	ASPP1 OE+AAV9-NC vs AAV9-shp53	TTC	↓	0.000000033	1.00	

↑ increased infarct size
↓ decreased infarct size
→ infarct size not changed
① endpoint for prospective power calculation other than infarct size
② endpoint for prospective power calculation not specified

* no n-values reported
gray shaded: studies without prospective power analysis and using the most reasonable infarct size endpoint
red shaded: studies with a prospective power analysis and using the most reasonable infarct size endpoint

Supplemental references

1. Belaidi, E; Decorps, J; Augeul, L; Durand, A; Ovize, M (2013) Endoplasmic reticulum stress contributes to heart protection induced by cyclophilin D inhibition. Basic Res Cardiol 108:363. doi:10.1007/s00395-013-0363-z

2. Bell, RM; Kunuthur, SP; Hendry, C; Bruce-Hickman, D; Davidson, S; Yellon, DM (2013) Matrix metalloproteinase inhibition protects CyPD knockout mice independently of RISK/mPTP signalling: a parallel pathway to protection. Basic Res Cardiol 108:331. doi:10.1007/s00395-013-0331-7

3. Contractor, H; Stottrup, NB; Cunnington, C; Manlhiot, C; Diesch, J; Ormerod, JOM; Jensen, R; Botker, HE; Redington, A; Schmidt, MR; Ashrafian, H; Kharbanda, RK (2013) Aldehyde dehydrogenase-2 inhibition blocks remote preconditioning in experimental and human models. Basic Res Cardiol 108:343. doi:10.1007/s00395-013-0343-3

4. Davidson, SM; Selvaraj, P; He, D; Boi-Doku, C; Yellon, RL; Vicencio, JM; Yellon, DM (2013) Remote ischaemic preconditioning involves signalling through the SDF-1 alpha/CXCR4 signalling axis. Basic Res Cardiol 108:377. doi:10.1007/s00395-013-0377-6

5. De Paulis, D; Chiari, P; Teixeira, G; Couture-Lepetit, E; Abrial, M; Argaud, L; Gharib, A; Ovize, M (2013) Cyclosporine A at reperfusion fails to reduce infarct size in the in vivo rat heart. Basic Res Cardiol 108:379. doi:10.1007/s00395-013-0379-4

6. Farah, C; Kleindienst, A; Bolea, G; Meyer, G; Gayrard, S; Geny, B; Obert, P; Cazorla, O; Tanguy, S; Reboul, C (2013) Exercise-induced cardioprotection: a role for eNOS uncoupling and NO metabolites. Basic Res Cardiol 108:389. doi:10.1007/s00395-013-0389-2

7. Gross, ER; Hsu, AK; Urban, TJ; Mochly-Rosen, D; Gross, GJ (2013) Nociceptive-induced myocardial remote conditioning is mediated by neuronal gamma protein kinase C. Basic Res Cardiol 108:381. doi:10.1007/s00395-013-0381-x

8. Lei, P; Baysa, A; Nebb, HI; Valen, G; Skomedal, T; Osnes, JB; Yang, ZQ; Haugen, F (2013) Activation of Liver X receptors in the heart leads to accumulation of intracellular lipids and attenuation of ischemia-reperfusion injury. Basic Res Cardiol 108:323. doi:10.1007/s00395-012-0323-z

9. Luo, T; Chen, BH; Zhao, ZL; He, NQ; Zeng, Z; Wu, B; Fukushima, Y; Dai, M; Huang, QB; Xu, DL; Bin, JP; Kitakaze, M; Liao, YL (2013) Histamine H2 receptor activation exacerbates myocardial ischemia/reperfusion injury by disturbing mitochondrial and endothelial function. Basic Res Cardiol 108:342. doi:10.1007/s00395-013-0342-4

10. Markowski, P; Boehm, O; Goelz, L; Haesner, AL; Ehrentraut, H; Bauerfeld, K; Tran, N; Zacharowski, K; Weisheit, C; Langhoff, P; Schwederski, M; Hilbert, T; Klaschik, S; Hoeft, A; Baumgarten, G; Meyer, R; Knuefermann, P (2013) Pre-conditioning with synthetic CpG-oligonucleotides attenuates myocardial ischemia/reperfusion injury via IL-10 up-regulation. Basic Res Cardiol 108:376. doi:10.1007/s00395-013-0376-7

11. Methner, C; Lukowski, R; Grube, K; Loga, F; Smith, RAJ; Murphy, MP; Hofmann, F; Krieg, T (2013) Protection through postconditioning or a mitochondria-targeted S-nitrosothiol is unaffected by cardiomyocyte-selective ablation of protein kinase G. Basic Res Cardiol 108:337. doi:10.1007/s00395-013-0337-1

12. Pei, HF; Yu, QJ; Xue, Q; Guo, YP; Sun, L; Hong, ZB; Han, H; Gao, EH; Qu, Y; Tao, L (2013) Notch1 cardioprotection in myocardial ischemia/reperfusion involves reduction of oxidative/nitrative stress. Basic Res Cardiol 108:373. doi:10.1007/s00395-013-0373-x

13. Pei, HF; Qu, Y; Lu, XY; Yu, QJ; Lian, K; Liu, PL; Yan, WJ; Liu, JY; Ma, YZ; Liu, Y; Li, CX; Li, WJ; Lau, WB; Zhang, HF; Tao, L (2013) Cardiac-derived adiponectin induced by long-term insulin treatment ameliorates myocardial ischemia/reperfusion injury in type 1 diabetic mice via AMPK signaling. Basic Res Cardiol 108:322. doi:10.1007/s00395-012-0322-0

14. Penna, C; Perrelli, MG; Tullio, F; Angotti, C; Camporeale, A; Poli, V; Pagliaro, P (2013) Diazoxide postconditioning induces mitochondrial protein S-Nitrosylation and a redox-sensitive mitochondrial phosphorylation/translocation of RISK elements: no role for SAFE. Basic Res Cardiol 108:371. doi:10.1007/s00395-013-0371-z

15. Sanchez, JA; Rodriguez-Sinovas, A; Barba, I; Miro-Casas, E; Fernandez-Sanz, C; Ruiz-Meana, M; Alburquerque-Bejar, JJ; Garcia-Dorado, D (2013) Activation of RISK and SAFE pathways is not involved in the effects of Cx43 deficiency on tolerance to ischemia-reperfusion injury and preconditioning protection. Basic Res Cardiol 108:351. doi:10.1007/s00395-013-0351-3

16. Su, H; Yuan, YX; Wang, XM; Lau, WB; Wang, YJ; Wang, XL; Gao, EH; Koch, WJ; Ma, XL (2013) Inhibition of CTRP9, a novel and cardiac-abundantly expressed cell survival molecule, by TNF alpha-initiated oxidative signaling contributes to exacerbated cardiac injury in diabetic mice. Basic Res Cardiol 108:315. doi:10.1007/s00395-012-0315-z

17. Sun, ZC; Tong, G; Ma, N; Li, JY; Li, XJ; Li, S; Zhou, JY; Xiong, LZ; Cao, F; Yao, LB; Wang, HC; Shen, L (2013) NDRG2: a newly identified mediator of insulin cardioprotection against myocardial ischemia-reperfusion injury. Basic Res Cardiol 108:341. doi:10.1007/s00395-013-0341-5

18. Wang, N; De Vuyst, E; Ponsaerts, R; Boengler, K; Palacios-Prado, N; Wauman, J; Lai, CP; De Bock, M; Decrock, E; Bol, M; Vinken, M; Rogiers, V; Tavernier, J; Evans, WH; Naus, CC; Bukauskas, FF; Sipido, KR; Heusch, G; Schulz, R; Bultynck, G; Leybaert, L (2013) Selective inhibition of Cx43 hemichannels by Gap19 and its impact on myocardial ischemia/reperfusion injury. Basic Res Cardiol 108:309. doi:10.1007/s00395-012-0309-x

19. Zhao, M; He, X; Bi, XY; Yu, XJ; Wier, WG; Zang, WJ (2013) Vagal stimulation triggers peripheral vascular protection through the cholinergic anti-inflammatory pathway in a rat model of myocardial ischemia/reperfusion. Basic Res Cardiol 108:345. doi:10.1007/s00395-013-0345-1

20. Garcia-Prieto, J; Garcia-Ruiz, JM; Sanz-Rosa, D; Pun, A; Garcia-Alvarez, A; Davidson, SM; Fernandez-Friera, L; Nuno-Ayala, M; Fernandez-Jimenez, R; Bernal, JA; Izquierdo-Garcia, JL; Jimenez-Borreguero, J; Pizarro, G; Ruiz-Cabello, J; Macaya, C; Fuster, V; Yellon, DM; Ibanez, B (2014) beta(3) adrenergic receptor selective stimulation during ischemia/reperfusion improves cardiac function in translational models through inhibition of mPTP opening in cardiomyocytes. Basic Res Cardiol 109:422. doi:10.1007/s00395-014-0422-0

21. Li, J; Rohailla, S; Gelber, N; Rutka, J; Sabah, N; Gladstone, RA; Wei, C; Hu, PZ; Kharbanda, RK; Redington, AN (2014) MicroRNA-144 is a circulating effector of remote ischemic preconditioning. Basic Res Cardiol 109:423. doi:10.1007/s00395-014-0423-z

22. Liu, Y; Lian, K; Zhang, LJ; Wang, RT; Yi, F; Gao, C; Xin, C; Zhu, D; Li, Y; Yan, WJ; Xiong, LZ; Gao, EH; Wang, HC; Tao, L (2014) TXNIP mediates NLRP3 inflammasome activation in cardiac microvascular endothelial cells as a novel mechanism in myocardial ischemia/reperfusion injury. Basic Res Cardiol 109:415. doi:10.1007/s00395-014-0415-z

23. Lu, MJ; Chen, YS; Huang, HS; Ma, MC (2014) Hypoxic preconditioning protects rat hearts against ischemia-reperfusion injury via the arachidonate12-lipoxygenase/transient receptor potential vanilloid 1 pathway. Basic Res Cardiol 109:414. doi:10.1007/s00395-014-0414-0

24. Merlocco, AC; Redington, KL; Disenhouse, T; Strantzas, SC; Gladstone, R; Wei, C; Tropak, MB; Manlhiot, C; Li, J; Redington, AN (2014) Transcutaneous electrical nerve stimulation as a novel method of remote preconditioning: in vitro validation in an animal model and first human observations. Basic Res Cardiol 109:406. doi:10.1007/s00395-014-0406-0

25. Merx, MW; Gorressen, S; van de Sandt, AM; Cortese-Krott, MM; Ohlig, J; Stern, M; Rassaf, T; Godecke, A; Gladwin, MT; Kelm, M (2014) Depletion of circulating blood NOS3 increases severity of myocardial infarction and left ventricular dysfunction. Basic Res Cardiol 109:398. doi:10.1007/s00395-013-0398-1

26. Muller, J; Gorressen, S; Grandoch, M; Feldmann, K; Kretschmer, I; Lehr, S; Ding, Z; Schmitt, JP; Schrader, J; Garbers, C; Heusch, G; Kelm, M; Scheller, J; Fischer, JW (2014) Interleukin-6-dependent phenotypic modulation of cardiac fibroblasts after acute myocardial infarction. Basic Res Cardiol 109:440. doi:10.1007/s00395-014-0440-y

27. Penna, C; Brancaccio, M; Tullio, F; Rubinetto, C; Perrelli, MG; Angotti, C; Pagliaro, P; Tarone, G (2014) Overexpression of the muscle-specific protein, melusin, protects from cardiac ischemia/reperfusion injury. Basic Res Cardiol 109:418. doi:10.1007/s00395-014-0418-9

28. Sun, LL; Chen, C; Jiang, BB; Li, YL; Deng, QP; Sun, M; An, XB; Yang, X; Yang, Y; Zhang, RL; Lu, Y; Zhu, DS; Huo, YQ; Feng, GS; Zhang, YY; Luo, JC (2014) Grb2-associated binder 1 is essential for cardioprotection against ischemia/reperfusion injury. Basic Res Cardiol 109:420. doi:10.1007/s00395-014-0420-2

29. Tseliou, E; Reich, H; de Couto, G; Terrovitis, J; Sun, BM; Liu, WX; Marban, E (2014) Cardiospheres reverse adverse remodeling in chronic rat myocardial infarction: roles of soluble endoglin and Tgf-beta signaling. Basic Res Cardiol 109:443. doi:10.1007/s00395-014-0443-8

30. Zhang, Y; Liu, XX; She, ZG; Jiang, DS; Wan, NA; Xia, H; Zhu, XH; Wei, X; Zhang, XD; Li, HL (2014) Interferon regulatory factor 9 is an essential mediator of heart dysfunction and cell death

following myocardial ischemia/reperfusion injury. Basic Res Cardiol 109:434. doi:10.1007/s00395-014-0434-9

31. Bao, MW; Cai, ZX; Zhang, XJ; Li, LP; Liu, XX; Wan, N; Hu, GY; Wan, FW; Zhang, R; Zhu, XY; Xia, H; Li, HL (2015) Dickkopf-3 protects against cardiac dysfunction and ventricular remodelling following myocardial infarction. Basic Res Cardiol 110:25. doi:10.1007/s00395-015-0481-x

32. Barsukevich, V; Basalay, M; Sanchez, J; Mrochek, A; Whittle, J; Ackland, GL; Gourine, AV; Gourine, A (2015) Distinct cardioprotective mechanisms of immediate, early and delayed ischaemic postconditioning. Basic Res Cardiol 110:452. doi:10.1007/s00395-014-0452-7

33. Baysa, A; Sagave, J; Carpi, A; Zaglia, T; Campesan, M; Dahl, CP; Bilbija, D; Troitskaya, M; Gullestad, L; Giorgio, M; Mongillo, M; Di Lisa, F; Vaage, JI; Valen, G (2015) The p66ShcA adaptor protein regulates healing after myocardial infarction. Basic Res Cardiol 110:13. doi:10.1007/s00395-015-0470-0

34. Das, A; Samidurai, A; Hoke, NN; Kukreja, RC; Salloum, FN (2015) Hydrogen sulfide mediates the cardioprotective effects of gene therapy with PKG-I alpha. Basic Res Cardiol 110:42. doi:10.1007/s00395-015-0500-y

35. Das, A; Salloum, FN; Filippone, SM; Durrant, DE; Rokosh, G; Bolli, R; Kukreja, RC (2015) Inhibition of mammalian target of rapamycin protects against reperfusion injury in diabetic heart through STAT3 signaling. Basic Res Cardiol 110:31. doi:10.1007/s00395-015-0486-5

36. Eisenhardt, SU; Weiss, JBW; Smolka, C; Maxeiner, J; Pankratz, F; Bemtgen, X; Kustermann, M; Thiele, JR; Schmidt, Y; Stark, GB; Moser, M; Bode, C; Grundmann, S (2015) MicroRNA-155 aggravates ischemia-reperfusion injury by modulation of inflammatory cell recruitment and the respiratory oxidative burst. Basic Res Cardiol 110:32. doi:10.1007/s00395-015-0490-9

37. Hernandez-Resendiz, S; Palma-Flores, C; De los Santos, S; Roman-Anguiano, NG; Flores, M; de la Pena, A; Flores, PL; Fernandez, JM; Coral-Vazquez, RM; Zazueta, C (2015) Reduction of no-reflow and reperfusion injury with the synthetic 17 beta-aminoestrogen compound Prolame is associated with PI3K/Akt/eNOS signaling cascade. Basic Res Cardiol 110:1. doi:10.1007/s00395-015-0464-y

38. Li, LP; Wang, XD; Chen, W; Qi, HY; Jiang, DS; Huang, L; Huang, FH; Wang, LM; Li, HL; Chen, X (2015) Regulatory role of CARD3 in left ventricular remodelling and dysfunction after myocardial infarction. Basic Res Cardiol 110:56. doi:10.1007/s00395-015-0515-4

39. Niu, JL; Jin, ZQ; Kim, H; Kolattukudy, PE (2015) MCP-1-induced protein attenuates post-infarct cardiac remodeling and dysfunction through mitigating NF-kappa B activation and suppressing inflammation-associated microRNA expression. Basic Res Cardiol 110:26. doi:10.1007/s00395-015-0483-8

40. Robinson, E; Cassidy, RS; Tate, M; Zhao, YY; Lockhart, S; Calderwood, D; Church, R; McGahon, MK; Brazil, DP; McDermott, BJ; Green, BD; Grieve, DJ (2015) Exendin-4 protects against post-myocardial infarction remodelling via specific actions on inflammation and the extracellular matrix. Basic Res Cardiol 110:20. doi:10.1007/s00395-015-0476-7

41. Szczepanek, K; Xu, AJ; Hu, Y; Thompson, J; He, J; Larner, AC; Salloum, FN; Chen, Q; Lesnefsky, EJ (2015) Cardioprotective function of mitochondrial-targeted and transcriptionally inactive STAT3 against ischemia and reperfusion injury. Basic Res Cardiol 110:53. doi:10.1007/s00395-015-0509-2

42. Tian, YK; French, BA; Kron, IL; Yang, ZQ (2015) Splenic leukocytes mediate the hyperglycemic exacerbation of myocardial infarct size in mice. Basic Res Cardiol 110:39. doi:10.1007/s00395-015-0496-3

43. Tian, YK; Marshall, M; French, BA; Linden, J; Yang, ZQ (2015) The infarct-sparing effect of IB-MECA against myocardial ischemia/reperfusion injury in mice is mediated by sequential activation of adeNOSine A(3) and A(2A) receptors. Basic Res Cardiol 110:16. doi:10.1007/s00395-015-0473-x

44. Uitterdijk, A; Yetgin, T; Hekkert, MTL; Sneep, S; Krabbendam-Peters, I; van Beusekom, HMM; Fischer, TM; Cornelussen, RN; Manintveld, OC; Merkus, D; Duncker, DJ (2015) Vagal nerve stimulation started just prior to reperfusion limits infarct size and no-reflow. Basic Res Cardiol 110:51. doi:10.1007/s00395-015-0508-3

45. Yan, L; Kudej, RK; Vatner, DE; Vatner, SF (2015) Myocardial ischemic protection in natural mammalian hibernation. Basic Res Cardiol 110:9. doi:10.1007/s00395-015-0462-0

46. Yang, XM; Cui, L; White, J; Kuck, J; Ruchko, MV; Wilson, GL; Alexeyev, M; Gillespie, MN; Downey, JM; Cohen, MV (2015) Mitochondrially targeted Endonuclease III has a powerful anti-infarct

effect in an in vivo rat model of myocardial ischemia/reperfusion. Basic Res Cardiol 110:3. doi:10.1007/s00395-014-0459-0

47. Zalewski, J; Claus, P; Bogaert, J; Vanden Driessche, N; Driesen, RB; Galan, DT; Sipido, KR; Buszman, P; Milewski, K; Van de Werf, F (2015) Cyclosporine A reduces microvascular obstruction and preserves left ventricular function deterioration following myocardial ischemia and reperfusion. Basic Res Cardiol 110:18. doi:10.1007/s00395-015-0475-8

48. Borrell-Pages, M; Vilahur, G; Romero, JC; Casani, L; Bejar, MT; Badimon, L (2016) LRP5/canonical Wnt signalling and healing of ischemic myocardium. Basic Res Cardiol 111:67. doi:10.1007/s00395-016-0585-y

49. Estrada, JA; Williams, AG; Sun, J; Gonzalez, L; Downey, HF; Caffrey, JL; Mallet, RT (2016) delta-Opioid receptor (DOR) signaling and reactive oxygen species (ROS) mediate intermittent hypoxia induced protection of canine myocardium. Basic Res Cardiol 111:17. doi:10.1007/s00395-016-0538-5

50. Fang, J; Hu, FD; Ke, D; Yan, YM; Liao, ZM; Yuan, X; Wu, LZ; Jiang, Q; Chen, LL (2016) N,N-dimethylsphingosine attenuates myocardial ischemia-reperfusion injury by recruiting regulatory T cells through PI3K/Akt pathway in mice. Basic Res Cardiol 111:32. doi:10.1007/s00395-016-0548-3

51. Inserte, J; Cardona, M; Poncelas-Nozal, M; Hernando, V; Vilardosa, U; Aluja, D; Parra, VM; Sanchis, D; Garcia-Dorado, D (2016) Studies on the role of apoptosis after transient myocardial ischemia: genetic deletion of the executioner caspases-3 and-7 does not limit infarct size and ventricular remodeling. Basic Res Cardiol 111:18. doi:10.1007/s00395-016-0537-6

52. Johnsen, J; Pryds, K; Salman, R; Lofgren, B; Kristiansen, SB; Botker, HE (2016) The remote ischemic preconditioning algorithm: effect of number of cycles, cycle duration and effector organ mass on efficacy of protection. Basic Res Cardiol 111:10. doi:10.1007/s00395-016-0529-6

53. Kleindienst, A; Battault, S; Belaidi, E; Tanguy, S; Rosselin, M; Boulghobra, D; Meyer, G; Gayrard, S; Walther, G; Geny, B; Durand, G; Cazorla, O; Reboul, C (2016) Exercise does not activate the beta(3) adrenergic receptor-eNOS pathway, but reduces inducible NOS expression to protect the heart of obese diabetic mice. Basic Res Cardiol 111:40. doi:10.1007/s00395-016-0559-0

54. Marchini, T; Wolf, D; Michel, NA; Mauler, M; Dufner, B; Hoppe, N; Beckert, J; Jackel, M; Magnani, N; Duerschmied, D; Tasat, D; Alvarez, S; Reinohl, J; von zur Muhlen, C; Idzko, M; Bode, C; Hilgendorf, I; Evelson, P; Zirlik, A (2016) Acute exposure to air pollution particulate matter aggravates experimental myocardial infarction in mice by potentiating cytokine secretion from lung macrophages. Basic Res Cardiol 111:44. doi:10.1007/s00395-016-0562-5

55. Pickard, JMJ; Davidson, SM; Hausenloy, DJ; Yellon, DM (2016) Co-dependence of the neural and humoral pathways in the mechanism of remote ischemic conditioning. Basic Res Cardiol 111:50. doi:10.1007/s00395-016-0568-z

56. Reforgiato, MR; Milano, G; Fabrias, G; Casas, J; Gasco, P; Paroni, R; Samaja, M; Ghidoni, R; Caretti, A; Signorelli, P (2016) Inhibition of ceramide de novo synthesis as a postischemic strategy to reduce myocardial reperfusion injury. Basic Res Cardiol 111:12. doi:10.1007/s00395-016-0533-x

57. Tian, YK; Pan, DF; Chordia, MD; French, BA; Kron, IL; Yang, ZQ (2016) The spleen contributes importantly to myocardial infarct exacerbation during post-ischemic reperfusion in mice via signaling between cardiac HMGB1 and splenic RAGE. Basic Res Cardiol 111:62. doi:10.1007/s00395-016-0583-0

58. Wang, XX; Wang, XL; Tong, MM; Gan, L; Chen, HL; Wu, SS; Chen, JX; Li, RL; Wu, Y; Zhang, HY; Zhu, Y; Li, YX; He, JH; Wang, MJ; Jiang, W (2016) SIRT6 protects cardiomyocytes against ischemia/reperfusion injury by augmenting FoxO3 alpha-dependent antioxidant defense mechanisms. Basic Res Cardiol 111:13. doi:10.1007/s00395-016-0531-z

59. Bei, YH; Xu, TZ; Lv, DC; Yu, PJ; Xu, JH; Che, L; Das, A; Tigges, J; Toxavidis, V; Ghiran, I; Shah, R; Li, YQ; Zhang, YH; Das, S; Xiao, JJ (2017) Exercise-induced circulating extracellular vesicles protect against cardiac ischemia-reperfusion injury. Basic Res Cardiol 112:38. doi:10.1007/s00395-017-0628-z

60. Blackburn, NJR; Vulesevic, B; McNeill, B; Cimenci, CE; Ahmadi, A; Gonzalez-Gomez, M; Ostojic, A; Zhong, ZY; Brownlee, M; Beisswenger, PJ; Milne, RW; Suuronen, EJ (2017) Methylglyoxal-derived advanced glycation end products contribute to negative cardiac remodeling and dysfunction post-myocardial infarction. Basic Res Cardiol 112:57. doi:10.1007/s00395-017-0646-x

61. Du, GQ; Shao, ZB; Wu, J; Yin, WJ; Li, SH; Wu, J; Weisel, RD; Tian, JW; Li, RK (2017) Targeted myocardial delivery of GDF11 gene rejuvenates the aged mouse heart and enhances myocardial

regeneration after ischemia-reperfusion injury. Basic Res Cardiol 112:7. doi:10.1007/s00395-016-0593-y

62. Gadicherla, AK; Wang, N; Bulic, M; Agullo-Pascual, E; Lissoni, A; De Smet, M; Delmar, M; Bultynck, G; Krysko, DV; Camara, A; Schluter, KD; Schulz, R; Kwok, WM; Leybaert, L (2017) Mitochondrial Cx43 hemichannels contribute to mitochondrial calcium entry and cell death in the heart. Basic Res Cardiol 112:27. doi:10.1007/s00395-017-0618-1

63. Garcia-Nino, WR; Correa, F; Rodriguez-Barrena, JI; Leon-Contreras, JC; Buelna-Chontal, M; Soria-Castro, E; Hernandez-Pando, R; Pedraza-Chaverri, J; Zazueta, C (2017) Cardioprotective kinase signaling to subsarcolemmal and interfibrillar mitochondria is mediated by caveolar structures. Basic Res Cardiol 112:15. doi:10.1007/s00395-017-0607-4

64. Garcia-Ruiz, JM; Galan-Arriola, C; Fernandez-Jimenez, R; Aguero, J; Sanchez-Gonzalez, J; Garcia-Alvarez, A; Nuno-Ayala, M; Dube, GP; Zafirelis, Z; Lopez-Martin, GJ; Bernal, JA; Lara-Pezzi, E; Fuster, V; Ibanez, B (2017) Bloodless reperfusion with the oxygen carrier HBOC-201 in acute myocardial infarction: a novel platform for cardioprotective probes delivery. Basic Res Cardiol 112:17. doi:10.1007/s00395-017-0605-6

65. Harisseh, R; Pillot, B; Gharib, A; Augeul, L; Gallo-Bona, N; Ferrera, R; Loufouat, J; Delale, T; Allas, S; Abribat, T; Da Silva, CC; Ovize, M (2017) Unacylated ghrelin analog prevents myocardial reperfusion injury independently of permeability transition pore. Basic Res Cardiol 112:4. doi:10.1007/s00395-016-0595-9

66. Hong, H; Tao, T; Chen, S; Liang, CQ; Qiu, Y; Zhou, YH; Zhang, R (2017) MicroRNA-143 promotes cardiac ischemia-mediated mitochondrial impairment by the inhibition of protein kinase Cepsilon. Basic Res Cardiol 112:60. doi:10.1007/s00395-017-0649-7

67. Hooshdaran, B; Kolpakov, MA; Guo, XJ; Miller, SA; Wang, T; Tilley, DG; Rafiq, K; Sabri, A (2017) Dual inhibition of cathepsin G and chymase reduces myocyte death and improves cardiac remodeling after myocardial ischemia reperfusion injury. Basic Res Cardiol 112:62. doi:10.1007/s00395-017-0652-z

68. Jamaiyar, A; Wan, WG; Ohanyan, V; Enrick, M; Janota, D; Cumpston, D; Song, H; Stevanov, K; Kolz, CL; Hakobyan, T; Dong, F; Newby, BMZ; Chilian, WM; Yin, LY (2017) Alignment of inducible vascular progenitor cells on a micro-bundle scaffold improves cardiac repair following myocardial infarction. Basic Res Cardiol 112:41. doi:10.1007/s00395-017-0631-4

69. Jung, MR; Ma, YG; Iyer, RP; DeLeon-Pennell, KY; Yabluchanskiy, A; Garrett, MR; Lindsey, ML (2017) IL-10 improves cardiac remodeling after myocardial infarction by stimulating M2 macrophage polarization and fibroblast activation. Basic Res Cardiol 112:33. doi:10.1007/s00395-017-0622-5

70. Kingery, JR; Hamid, T; Lewis, RK; Ismahil, MA; Bansal, SS; Rokosh, G; Townes, TM; Ildstad, ST; Jones, SP; Prabhu, SD (2017) Leukocyte iNOS is required for inflammation and pathological remodeling in ischemic heart failure. Basic Res Cardiol 112:19. doi:10.1007/s00395-017-0609-2

71. Lassen, TR; Nielsen, JM; Johnsen, J; Ringgaard, S; Botker, HE; Kristiansen, SB (2017) Effect of paroxetine on left ventricular remodeling in an in vivo rat model of myocardial infarction. Basic Res Cardiol 112:26. doi:10.1007/s00395-017-0614-5

72. Maciel, L; de Oliveira, DF; da Costa, GCV; Bisch, PM; Nascimento, JHM (2017) Cardioprotection by the transfer of coronary effluent from ischaemic preconditioned rat hearts: identification of cardioprotective humoral factors. Basic Res Cardiol 112:52. doi:10.1007/s00395-017-0641-2

73. Pickard, JMJ; Burke, N; Davidson, SM; Yellon, DM (2017) Intrinsic cardiac ganglia and acetylcholine are important in the mechanism of ischaemic preconditioning. Basic Res Cardiol 112:11. doi:10.1007/s00395-017-0601-x

74. Pischke, SE; Gustavsen, A; Orrem, HL; Egge, KH; Courivaud, F; Fontenelle, H; Despont, A; Bongoni, AK; Rieben, R; Tonnessen, TI; Nunn, MA; Scott, H; Skulstad, H; Barratt-Due, A; Mollnes, TE (2017) Complement factor 5 blockade reduces porcine myocardial infarction size and improves immediate cardiac function. Basic Res Cardiol 112:20. doi:10.1007/s00395-017-0610-9

75. Rienks, M; Carai, P; Bitsch, N; Schellings, M; Vanhaverbeke, M; Verjans, J; Cuijpers, I; Heymans, S; Papageorgiou, A (2017) Sema3A promotes the resolution of cardiac inflammation after myocardial infarction. Basic Res Cardiol 112:42. doi:10.1007/s00395-017-0630-5

76. Rossello, X; Riquelme, JA; He, ZH; Taferner, S; Vanhaesebroeck, B; Davidson, SM; Yellon, DM (2017) The role of PI3K alpha isoform in cardioprotection. Basic Res Cardiol 112:66. doi:10.1007/s00395-017-0657-7

77. Tobisawa, T; Yano, T; Tanno, M; Miki, T; Kuno, A; Kimura, Y; Ishikawa, S; Kouzu, H; Nishizawa, K; Yoshida, H; Miura, T (2017) Insufficient activation of Akt upon reperfusion because of its novel modification by reduced PP2A-B55 alpha contributes to enlargement of infarct size by chronic kidney disease. Basic Res Cardiol 112:31. doi:10.1007/s00395-017-0621-6

78. Uitterdijk, A; Springeling, T; Hermans, KCM; Merkus, D; de Beer, VJ; Gorsse-Bakker, C; Mokelke, E; Daskalopoulos, EP; Wielopolski, PA; Cleutjens, JPM; Blankesteijn, WM; Prinzen, FW; van der Giessen, WJ; van Geuns, RJM; Duncker, DJ (2017) Intermittent pacing therapy favorably modulates infarct remodeling. Basic Res Cardiol 112:28. doi:10.1007/s00395-017-0616-3

79. Yang, G; Zhang, XJ; Weng, XY; Liang, P; Dai, X; Zeng, S; Xu, HH; Huan, HL; Fang, MM; Li, YH; Xu, DC; Xu, Y (2017) SUV39H1 mediated SIRT1 trans-repression contributes to cardiac ischemia-reperfusion injury. Basic Res Cardiol 112:22. doi:10.1007/s00395-017-0608-3

80. Audia, JP; Yang, XM; Crockett, ES; Housley, N; Ul Haq, E; O'Donnell, K; Cohen, MV; Downey, JM; Alvarez, DF (2018) Caspase-1 inhibition by VX-765 administered at reperfusion in P2Y(12) receptor antagonist-treated rats provides long-term reduction in myocardial infarct size and preservation of ventricular function. Basic Res Cardiol 113:32. doi:10.1007/s00395-018-0692-z

81. Kleinbongard, P; Skyschally, A; Gent, S; Pesch, M; Heusch, G (2018) STAT3 as a common signal of ischemic conditioning: a lesson on rigor and reproducibility in preclinical studies on cardioprotection. Basic Res Cardiol 113:3. doi:10.1007/s00395-017-0660-z

82. Kohler, D; Bibli, SI; Klammer, LP; Roth, JM; Lehmann, R; Fleming, I; Granja, TF; Straub, A; Benz, PM; Rosenberger, P (2018) Phosphorylation of vasodilator-stimulated phosphoprotein contributes to myocardial ischemic preconditioning. Basic Res Cardiol 113:11. doi:10.1007/s00395-018-0667-0

83. Nuntaphum, W; Pongkan, W; Wongjaikam, S; Thummasorn, S; Tanajak, P; Khamseekaew, J; Intachai, K; Chattipakorn, SC; Chattipakorn, N; Shinlapawittayatorn, K (2018) Vagus nerve stimulation exerts cardioprotection against myocardial ischemia/reperfusion injury predominantly through its efferent vagal fibers. Basic Res Cardiol 113:22. doi:10.1007/s00395-018-0683-0

84. Wider, J; Undyala, VVR; Whittaker, P; Woods, J; Chen, XQ; Przyklenk, K (2018) Remote ischemic preconditioning fails to reduce infarct size in the Zucker fatty rat model of type-2 diabetes: role of defective humoral communication. Basic Res Cardiol 113:16. doi:10.1007/s00395-018-0674-1

85. Yellon, DM; He, ZH; Khambata, R; Ahluwalia, A; Davidson, SM (2018) The GTN patch: a simple and effective new approach to cardioprotection?. Basic Res Cardiol 113:20. doi:10.1007/s00395-018-0681-2

86. Alakoski, T; Ulvila, J; Yrjola, R; Vainio, L; Magga, J; Szabo, Z; Licht, JD; Kerkela, R (2019) Inhibition of cardiomyocyte Sprouty1 protects from cardiac ischemia-reperfusion injury. Basic Res Cardiol 114:7. doi:10.1007/s00395-018-0713-y

87. Amanakis, G; Kleinbongard, P; Heusch, G; Skyschally, A (2019) Attenuation of ST-segment elevation after ischemic conditioning maneuvers reflects cardioprotection online. Basic Res Cardiol 114:22. doi:10.1007/s00395-019-0732-3

88. Baker, HE; Kiel, AM; Luebbe, ST; Simon, BR; Earl, CC; Regmi, A; Roell, WC; Mather, KJ; Tune, JD; Goodwill, AG (2019) Inhibition of sodium-glucose cotransporter-2 preserves cardiac function during regional myocardial ischemia independent of alterations in myocardial substrate utilization. Basic Res Cardiol 114:25. doi:10.1007/s00395-019-0733-2

89. Billah, M; Ridiandries, A; Rayner, BS; Allahwala, UK; Dona, A; Khachigian, LM; Bhindi, R (2019) Egr-1 functions as a master switch regulator of remote ischemic preconditioning-induced cardioprotection. Basic Res Cardiol 115:3. doi:10.1007/s00395-019-0763-9

90. Birnbaum, Y; Tran, D; Bajaj, M; Ye, YM (2019) DPP-4 inhibition by linagliptin prevents cardiac dysfunction and inflammation by targeting the Nlrp3/ASC inflammasome. Basic Res Cardiol 114:35. doi:10.1007/s00395-019-0743-0

91. Guo, YR; Nong, YB; Tukaye, DN; Rokosh, G; Du, JJ; Zhu, XP; Book, M; Tomlin, A; Li, QH; Bolli, R (2019) Inducible cardiac-specific overexpression of cyclooxygenase-2 (COX-2) confers resistance to ischemia/reperfusion injury. Basic Res Cardiol 114:32. doi:10.1007/s00395-019-0741-2

92. Kohlhauer, M; Pell, VR; Burger, N; Spiroski, AM; Gruszczyk, A; Mulvey, JF; Mottahedin, A; Costa, ASH; Frezza, C; Ghaleh, B; Murphy, MP; Tissier, R; Krieg, T (2019) Protection against cardiac ischemia-reperfusion injury by hypothermia and by inhibition of succinate accumulation and oxidation is additive. Basic Res Cardiol 114:18. doi:10.1007/s00395-019-0727-0

93. Su, HH; Liao, JM; Wang, YH; Chen, KM; Lin, CW; Lee, IH; Li, YJ; Huang, JY; Tsai, SK; Yen, JC; Huang, SS (2019) Exogenous GDF11 attenuates non-canonical TGF- signaling to protect the heart from acute myocardial ischemia-reperfusion injury. Basic Res Cardiol 114:20. doi:10.1007/s00395-019-0728-z

94. ter Horst, EN; Krijnen, PAJ; Hakimzadeh, N; Robbers, LFHJ; Hirsch, A; Nijveldt, R; Lommerse, I; Fontijn, RD; Meinster, E; Delewi, R; van Royen, N; Zijlstra, F; van Rossum, AC; van der Schoot, CE; Kraan, TCTMV; Horrevoets, AJ; van der Laan, AM; Niessen, HWM; Piek, JJ (2019) Elevated monocyte-specific type I interferon signalling correlates positively with cardiac healing in myocardial infarct patients but interferon alpha application deteriorates myocardial healing in rats. Basic Res Cardiol 114:1. doi:10.1007/s00395-018-0709-7

95. Wang, MJ; Smith, K; Yu, Q; Miller, C; Singh, K; Sen, CK (2019) Mitochondrial connexin 43 in sex-dependent myocardial responses and estrogen-mediated cardiac protection following acute ischemia/reperfusion injury. Basic Res Cardiol 115:1. doi:10.1007/s00395-019-0759-5

96. Wu, L; Tan, JL; Chen, ZY; Huang, G (2019) Cardioprotection of post-ischemic moderate ROS against ischemia/reperfusion via STAT3-induced the inhibition of MCU opening. Basic Res Cardiol 114:39. doi:10.1007/s00395-019-0747-9

97. Bakhta, O; Pascaud, A; Dieu, X; Beaumont, J; Nzoughet, JK; Kamel, R; Croyal, M; Tamareille, S; Simard, G; de la Barca, JMC; Reynier, P; Prunier, F; Mirebeau-Prunier, D (2020) Tryptophane-kynurenine pathway in the remote ischemic conditioning mechanism. Basic Res Cardiol 115:13. doi:10.1007/s00395-019-0770-x

98. Behrouzi, B; Weyers, JJ; Qi, XL; Barry, J; Rabadia, V; Manca, D; Connelly, J; Spino, M; Wood, JC; Strauss, BH; Wright, GA; Ghugre, NR (2020) Action of iron chelator on intramyocardial hemorrhage and cardiac remodeling following acute myocardial infarction. Basic Res Cardiol 115:24. doi:10.1007/s00395-020-0782-6

99. He, ZH; Davidson, SM; Yellon, DM (2020) The importance of clinically relevant background therapy in cardioprotective studies. Basic Res Cardiol 115:69. doi:10.1007/s00395-020-00830-y

100. Heimerl, M; Sieve, I; Ricke-Hoch, M; Erschow, S; Battmer, K; Scherr, M; Hilfiker-Kleiner, D (2020) Neuraminidase-1 promotes heart failure after ischemia/reperfusion injury by affecting cardiomyocytes and invading monocytes/macrophages. Basic Res Cardiol 115:62. doi:10.1007/s00395-020-00821-z

101. Liu, SJ; Chen, J; Shi, J; Zhou, WY; Wang, L; Fang, WL; Zhong, Y; Chen, XH; Chen, YF; Sabri, A; Liu, SM (2020) M1-like macrophage-derived exosomes suppress angiogenesis and exacerbate cardiac dysfunction in a myocardial infarction microenvironment. Basic Res Cardiol 115:22. doi:10.1007/s00395-020-0781-7

102. Lobo-Gonzalez, M; Galan-Arriola, C; Rossello, X; Gonzalez-Del-Hoyo, M; Vilchez, JP; Higuero-Verdejo, MI; Garcia-Ruiz, JM; Lopez-Martin, GJ; Sanchez-Gonzalez, J; Oliver, E; Pizarro, G; Fuster, V; Ibanez, B (2020) Metoprolol blunts the time-dependent progression of infarct size. Basic Res Cardiol 115:55. doi:10.1007/s00395-020-0812-4

103. Mendieta, G; Ben-Aicha, S; Casani, L; Badimon, L; Sabate, M; Vilahur, G (2020) Molecular pathways involved in the cardioprotective effects of intravenous statin administration during ischemia. Basic Res Cardiol 115:2. doi:10.1007/s00395-019-0760-z

104. Sawashita, Y; Hirata, N; Yoshikawa, Y; Terada, H; Tokinaga, Y; Yamakage, M (2020) Remote ischemic preconditioning reduces myocardial ischemia-reperfusion injury through unacylated ghrelin-induced activation of the JAK/STAT pathway. Basic Res Cardiol 115:50. doi:10.1007/s00395-020-0809-z

105. Sun, X; Wei, ZL; Li, YY; Wang, JZ; Hu, JX; Yin, Y; Xie, J; Xu, B (2020) Renal denervation restrains the inflammatory response in myocardial ischemia-reperfusion injury. Basic Res Cardiol 115:15. doi:10.1007/s00395-020-0776-4

106. Takov, K; He, ZH; Johnston, HE; Timms, JF; Guillot, PV; Yellon, DM; Davidson, SM (2020) Small extracellular vesicles secreted from human amniotic fluid mesenchymal stromal cells possess cardioprotective and promigratory potential. Basic Res Cardiol 115:26. doi:10.1007/s00395-020-0785-3

107. Vatner, DE; Oydanich, M; Zhang, J; Babici, D; Vatner, SF (2020) Secreted frizzled-related protein 2, a novel mechanism to induce myocardial ischemic protection through angiogenesis. Basic Res Cardiol 115:48. doi:10.1007/s00395-020-0808-0

108. Wang, JZ; Chen, ZW; Dai, Q; Zhao, JX; Wei, ZL; Hui, JX; Sun, X; Xie, J; Xu, B (2020) Intravenously delivered mesenchymal stem cells prevent microvascular obstruction formation after myocardial ischemia/reperfusion injury. Basic Res Cardiol 115:40. doi:10.1007/s00395-020-0800-8

109. Zhou, H; Toan, S; Zhu, PJ; Wang, J; Ren, J; Zhang, YM (2020) DNA-PKcs promotes cardiac ischemia reperfusion injury through mitigating BI-1-governed mitochondrial homeostasis. Basic Res Cardiol 115:11. doi:10.1007/s00395-019-0773-7

110. Cheng, ZJ; Zhang, H; Zhang, L; Wang, XJ; Zhang, QJ; Feng, MW; Hu, DL; Li, H; Qian, LM (2021) Exercise-induced peptide TAG-23 protects cardiomyocytes from reperfusion injury through regulating PKG-cCbl interaction. Basic Res Cardiol 116:41. doi:10.1007/s00395-021-00878-4

111. Hekkert, MT; Newton, G; Chapman, K; Aqil, R; Downham, R; Yan, R; Merkus, D; Whitlock, G; Lane, CAL; Cawkill, D; Perrior, T; Duncker, DJ; Schneider, MD (2021) Preclinical trial of a MAP4K4 inhibitor to reduce infarct size in the pig: does cardioprotection in human stem cell-derived myocytes predict success in large mammals?. Basic Res Cardiol 116:34. doi:10.1007/s00395-021-00875-7

112. Hirschhauser, C; Lissoni, A; Gorge, PM; Lampe, PD; Heger, J; Schluter, KD; Leybaert, L; Schulz, R; Boengler, K (2021) Connexin 43 phosphorylation by casein kinase 1 is essential for the cardioprotection by ischemic preconditioning. Basic Res Cardiol 116:21. doi:10.1007/s00395-021-00861-z

113. Lai, Q; Yuan, GY; Shen, L; Zhang, L; Fu, F; Liu, ZL; Zhang, YY; Kou, JP; Liu, SJ; Yu, BY; Li, F (2021) Oxoeicosanoid receptor inhibition alleviates acute myocardial infarction through activation of BCAT1. Basic Res Cardiol 116:3. doi:10.1007/s00395-021-00844-0

114. Li, S; Chen, JX; Liu, MY; Chen, YQ; Wu, Y; Li, QY; Ma, T; Gao, JF; Xia, Y; Fan, MK; Chen, A; Lu, DB; Su, EY; Xu, F; Chen, ZW; Qian, JY; Ge, JB (2021) Protective effect of HINT2 on mitochondrial function via repressing MCU complex activation attenuates cardiac microvascular ischemia-reperfusion injury. Basic Res Cardiol 116:65. doi:10.1007/s00395-021-00905-4

115. Lieder, HR; Braczko, F; Gedik, N; Stroetges, M; Heusch, G; Kleinbongard, P (2021) Cardioprotection by post-conditioning with exogenous triiodothyronine in isolated perfused rat hearts and isolated adult rat cardiomyocytes. Basic Res Cardiol 116:27. doi:10.1007/s00395-021-00868-6

116. Liu, N; Kataoka, M; Wang, YC; Pu, LB; Dong, XX; Fu, XY; Zhang, F; Gao, F; Liang, T; Pei, JQ; Xiao, CC; Qiu, QZ; Hong, TT; Chen, QM; Zhao, J; Zhu, LL; He, JH; Hu, XY; Nie, Y; Zhu, W; Yu, H; Cowan, DB; Hu, XY; Wang, JA; Wang, DZ; Chen, JH (2021) LncRNA LncHrt preserves cardiac metabolic homeostasis and heart function by modulating the LKB1-AMPK signaling pathway. Basic Res Cardiol 116:48. doi:10.1007/s00395-021-00887-3

117. Zhang, HL; Yang, NZ; He, HY; Chai, JW; Cheng, XX; Zhao, HH; Zhou, DM; Teng, TM; Kong, XR; Yang, Q; Xu, ZL (2021) The zinc transporter ZIP7 (Slc39a7) controls myocardial reperfusion injury by regulating mitophagy. Basic Res Cardiol 116:54. doi:10.1007/s00395-021-00894-4

118. Fu, WB; Ren, HM; Shou, JL; Liao, Q; Li, LP; Shi, Y; Jose, PA; Zeng, CY; Wang, WE (2022) Loss of NPPA-AS1 promotes heart regeneration by stabilizing SFPQ-NONO heteromer-induced DNA repair. Basic Res Cardiol 117:10. doi:10.1007/s00395-022-00921-y

119. Hess, A; Borchert, T; Ross, TL; Bengel, FM; Thackeray, JT (2022) Characterizing the transition from immune response to tissue repair after myocardial infarction by multiparametric imaging. Basic Res Cardiol 117:14. doi:10.1007/s00395-022-00922-x

120. Jiao, T; Collado, A; Mahdi, A; Jurga, J; Tengbom, J; Saleh, N; Verouhis, D; Bohm, F; Zhou, ZC; Yang, JN; Pernow, J (2022) Erythrocytes from patients with ST-elevation myocardial infarction induce cardioprotection through the purinergic P2Y(13) receptor and nitric oxide signaling. Basic Res Cardiol 117:46. doi:10.1007/s00395-022-00953-4

121. Kleinbongard, P; Lieder, HR; Skyschally, A; Alloosh, M; Godecke, A; Rahmann, S; Sturek, M; Heusch, G (2022) Non-responsiveness to cardioprotection by ischaemic preconditioning in Ossabaw minipigs with genetic predisposition to, but without the phenotype of the metabolic syndrome. Basic Res Cardiol 117:58. doi:10.1007/s00395-022-00965-0

122. Nikolaou, PE; Mylonas, N; Makridakis, M; Makrecka-Kuka, M; Iliou, A; Zerikiotis, S; Efentakis, P; Kampoukos, S; Kostomitsopoulos, N; Vilskersts, R; Ikonomidis, I; Lambadiari, V; Zuurbier, CJ; Latosinska, A; Vlahou, A; Dimitriadis, G; Iliodromitis, EK; Andreadou, I (2022) Cardioprotection by selective SGLT-2 inhibitors in a non-diabetic mouse model of myocardial ischemia/reperfusion injury: a class or a drug effect?. Basic Res Cardiol 117:27. doi:10.1007/s00395-022-00934-7

123. Starz, C; Hardtner, C; Mauler, M; Dufner, B; Hoppe, N; Krebs, K; Ehlert, CA; Merz, J; Heidt, T; Stachon, P; Wolf, D; Bode, C; von zur Muehlen, C; Rottbauer, W; Gawaz, M; Duerschmied, D;

Leuschner, F; Borst, O; Westermann, D; Hilgendorf, I (2022) Elevated platelet-leukocyte complexes are associated with, but dispensable for myocardial ischemia-reperfusion injury. Basic Res Cardiol 117:61. doi:10.1007/s00395-022-00970-3

124. Wu, C; Liu, RR; Luo, ZF; Sun, MY; Qile, M; Xu, SJ; Jin, SY; Zhang, L; Gross, ER; Zhang, Y; He, SF (2022) Spinal cord astrocytes regulate myocardial ischemia-reperfusion injury. Basic Res Cardiol 117:56. doi:10.1007/s00395-022-00968-x

125. Xing, YH; Sui, ZH; Liu, YC; Wang, MM; Wei, XQ; Lu, QX; Wang, XY; Liu, N; Lu, C; Chen, R; Wu, MM; Wang, YQ; Zhao, YH; Guo, F; Cao, JL; Qi, JS; Wang, WY (2022) Blunting TRPML1 channels protects myocardial ischemia/reperfusion injury by restoring impaired cardiomyocyte autophagy. Basic Res Cardiol 117:20. doi:10.1007/s00395-022-00930-x

126. Xu, Y; Jiang, K; Chen, F; Qian, J; Wang, DD; Wu, YZ; Zhou, CJ; Yu, Y; Chen, K; Hwa, O; Yang, B; Wang, HY; Xiang, YZ (2022) Bone marrow-derived naive B lymphocytes improve heart function after myocardial infarction: a novel cardioprotective mechanism for empagliflozin. Basic Res Cardiol 117:47. doi:10.1007/s00395-022-00956-1

127. Bassiouni, W; Valencia, R; Mahmud, Z; Seubert, JM; Schulz, R (2023) Matrix metalloproteinase-2 proteolyzes mitofusin-2 and impairs mitochondrial function during myocardial ischemia-reperfusion injury. Basic Res Cardiol 118:29. doi:10.1007/s00395-023-00999-y

128. Dang, YN; Hua, WJ; Zhang, XT; Sun, H; Zhang, YJ; Yu, BB; Wang, SR; Zhang, M; Kong, ZH; Pan, DJ; Chen, Y; Li, SR; Yuan, L; Reinhardt, JD; Lu, X; Zheng, Y (2023) Anti-angiogenic effect of exo-LncRNA TUG1 in myocardial infarction and modulation by remote ischemic conditioning. Basic Res Cardiol 118:1. doi:10.1007/s00395-022-00975-y

129. Kleinbongard, P.; Lieder, H.R.; Skyschally, A. (2023) No robust reduction of infarct size and no-reflow by metoprolol pretreatment in adult Göttingen minipigs. Basic Res Cardiol 118:23. doi:10.1007/s00395-023-00993-4

130. Konijnenberg, LSF; Luiken, TTJ; Veltien, A; Uthman, L; Kuster, CTA; Rodwell, L; de Waard, GA; Lindert, MKT; Akiva, A; Thijssen, DHJ; Nijveldt, R; van Royen, N (2023) Imatinib attenuates reperfusion injury in a rat model of acute myocardial infarction. Basic Res Cardiol 118:2. doi:10.1007/s00395-022-00974-z

131. Schneckmann, R; Doring, M; Gerfer, S; Gorressen, S; Heitmeier, S; Helten, C; Polzin, A; Jung, C; Kelm, M; Fender, AC; Flogel, U; Grandoch, M (2023) Rivaroxaban attenuates neutrophil maturation in the bone marrow niche. Basic Res Cardiol 118:31. doi:10.1007/s00395-023-01001-5

132. Schunke, KJ; Rodriguez, J; Dyavanapalli, J; Schloen, J; Wang, X; Escobar, J; Kowalik, G; Cheung, EC; Ribeiro, C; Russo, R; Alber, BR; Dergacheva, O; Chen, SW; Murillo-Berlioz, AE; Lee, KB; Trachiotis, G; Entcheva, E; Brantner, CA; Mendelowitz, D; Kay, MW (2023) Outcomes of hypothalamic oxytocin neuron-driven cardioprotection after acute myocardial infarction. Basic Res Cardiol 118:43. doi:10.1007/s00395-023-01013-1

133. Szulik, MW; Valdez, S; Walsh, M; Davis, K; Bia, R; Horiuchi, E; O'Very, S; Laxman, AK; Sandaklie-Nicolova, L; Eberhardt, DR; Durrant, JR; Sheikh, H; Hickenlooper, S; Creed, M; Brady, C; Miller, M; Wang, L; Garcia-Llana, J; Tracy, C; Drakos, SG; Funai, K; Chaudhuri, D; Boudina, S; Franklin, S (2023) SMYD1a protects the heart from ischemic injury by regulating OPA1-mediated cristae remodeling and supercomplex formation. Basic Res Cardiol 118:20. doi:10.1007/s00395-023-00991-6

134. Teixeira, RB; Pfeiffer, M; Zhang, P; Shafique, E; Rayta, B; Karbasiafshar, C; Ahsan, N; Sellke, FW; Abid, MR (2023) Reduction in mitochondrial ROS improves oxidative phosphorylation and provides resilience to coronary endothelium in non-reperfused myocardial infarction. Basic Res Cardiol 118:3. doi:10.1007/s00395-022-00976-x

135. Umbarkar, P; Ejantkar, S; Ramirez, SYR; Cora, AT; Zhang, QK; Tousif, S; Lal, H (2023) Cardiac fibroblast GSK-3a aggravates ischemic cardiac injury by promoting fibrosis, inflammation, and impairing angiogenesis. Basic Res Cardiol 118:35. doi:10.1007/s00395-023-01005-1

136. Xie, DN; Guo, HL; Li, MB; Jia, LQ; Zhang, H; Liang, DG; Wu, NS; Yang, ZQ; Tian, YK (2023) Splenic monocytes mediate inflammatory response and exacerbate myocardial ischemia/reperfusion injury in a mitochondrial cell-free DNA-TLR9-NLRP3-dependent fashion. Basic Res Cardiol 118:44. doi:10.1007/s00395-023-01014-0

137. Xie, J; Jiang, LJ; Wang, JZ; Yin, Y; Wang, RL; Du, LP; Chen, T; Ni, ZC; Qiao, SH; Gong, H; Xu, B; Xu, QB (2023) Multilineage contribution of CD34+ cells in cardiac remodeling after ischemia/reperfusion injury. Basic Res Cardiol 118:17. doi:10.1007/s00395-023-00981-8

138. Zhong, LF; Han, JB; Fan, XX; Huang, ZQ; Su, L; Cai, XL; Lin, S; Chen, XD; Huang, WJ; Dai, SS; Ye, BZ (2023) Novel GSDMD inhibitor GI-Y1 protects heart against pyroptosis and ischemia/reperfusion injury by blocking pyroptotic pore formation. Basic Res Cardiol 118:40. doi:10.1007/s00395-023-01010-4

139. Frasier, CR; Moukdar, F; Patel, HD; Sloan, RC; Stewart, LM; Alleman, RJ; La Favor, JD; Brown, DA (2013) Redox-dependent increases in glutathione reductase and exercise preconditioning: role of NADPH oxidase and mitochondria. Cardiovasc Res 98:47-55. doi:10.1093/cvr/cvt009

140. Hiroi, T; Wajima, T; Negoro, T; Ishii, M; Nakano, Y; Kiuchi, Y; Mori, Y; Shimizu, S (2013) Neutrophil TRPM2 channels are implicated in the exacerbation of myocardial ischaemia/reperfusion injury. Cardiovasc Res 97:271-281. doi:10.1093/cvr/cvs332

141. Jiang, BM; Zhang, B; Liang, PF; Chen, GW; Zhou, B; Lv, CL; Tu, ZZ; Xiao, XZ (2013) Nucleolin protects the heart from ischaemiareperfusion injury by up-regulating heat shock protein 32. Cardiovasc Res 99:92-101. doi:10.1093/cvr/cvt085

142. Li, JJ; Zhang, YY; Li, CF; Xie, J; Liu, Y; Zhu, WN; Zhang, XJ; Jiang, SR; Liu, L; Ding, ZN (2013) HSPA12B attenuates cardiac dysfunction and remodelling after myocardial infarction through an eNOS-dependent mechanism. Cardiovasc Res 99:674-684. doi:10.1093/cvr/cvt139

143. Liu, Y; Ye, XY; Mao, LN; Cheng, ZK; Yao, XP; Jia, XH; Mao, D; Ou, LL; Li, ZJ; Che, YZ; Liu, N; Steinhoff, G; Liu, L; Kong, DL (2013) Transplantation of parthenogenetic embryonic stem cells ameliorates cardiac dysfunction and remodelling after myocardial infarction. Cardiovasc Res 97:208-218. doi:10.1093/cvr/cvs314

144. Pereira, FE; Cronin, C; Ghosh, M; Zhou, SY; Agosto, M; Subramani, J; Wang, RB; Shen, JB; Schacke, W; Liang, B; Yang, TH; McAulliffe, B; Liang, BT; Shapiro, LH (2013) CD13 is essential for inflammatory trafficking and infarct healing following permanent coronary artery occlusion in mice. Cardiovasc Res 100:74-83. doi:10.1093/cvr/cvt155

145. Sandanger, O; Ranheim, T; Vinge, LE; Bliksoen, M; Alfsnes, K; Finsen, AV; Dahl, CP; Askevold, ET; Florholmen, G; Christensen, G; Fitzgerald, KA; Lien, E; Valen, G; Espevik, T; Aukrust, P; Yndestad, A (2013) The NLRP3 inflammasome is up-regulated in cardiac fibroblasts and mediates myocardial ischaemiareperfusion injury. Cardiovasc Res 99:164-174. doi:10.1093/cvr/cvt091

146. Uchinaka, A; Kawaguchi, N; Hamada, Y; Mori, S; Miyagawa, S; Saito, A; Sawa, Y; Matsuura, N (2013) Transplantation of myoblast sheets that secrete the novel peptide SVVYGLR improves cardiac function in failing hearts. Cardiovasc Res 99:102-110. doi:10.1093/cvr/cvt088

147. Wang, XH; Ha, TZ; Liu, L; Zou, JH; Zhang, X; Kalbfleisch, J; Gao, X; Williams, D; Li, CF (2013) Increased expression of microRNA-146a decreases myocardial ischaemia/reperfusion injury. Cardiovasc Res 97:432-442. doi:10.1093/cvr/cvs356

148. Whittington, HJ; Harding, I; Stephenson, CIM; Bell, R; Hausenloy, DJ; Mocanu, MM; Yellon, DM (2013) Cardioprotection in the aging, diabetic heart: the loss of protective Akt signalling. Cardiovasc Res 99:694-704. doi:10.1093/cvr/cvt140

149. Yang, CJ; Talukder, MAH; Varadharaj, S; Velayutham, M; Zweier, JL (2013) Early ischaemic preconditioning requires Akt- and PKA-mediated activation of eNOS via serine1176 phosphorylation. Cardiovasc Res 97:33-43. doi:10.1093/cvr/cvs287

150. Barile, L; Lionetti, V; Cervio, E; Matteucci, M; Gherghiceanu, M; Popescu, LM; Torre, T; Siclari, F; Moccetti, T; Vassalli, G (2014) Extracellular vesicles from human cardiac progenitor cells inhibit cardiomyocyte apoptosis and improve cardiac function after myocardial infarction. Cardiovasc Res 103:530-541. doi:10.1093/cvr/cvu167

151. Bice, JS; Keim, Y; Stasch, JP; Baxter, GF (2014) NO-independent stimulation or activation of soluble guanylyl cyclase during early reperfusion limits infarct size. Cardiovasc Res 101:220-228. doi:10.1093/cvr/cvt257

152. De Hoog, VC; Timmers, L; Van Duijvenvoorde, A; De Jager, SCA; Van Middelaar, BJ; Smeets, MB; Woodruff, TM; Doevendans, PA; Pasterkamp, G; Hack, CE; De Kleijn, DPV (2014) Leucocyte expression of complement C5a receptors exacerbates infarct size after myocardial reperfusion injury. Cardiovasc Res 103:521-529. doi:10.1093/cvr/cvu153

153. Gu, JM; Fan, YQ; Liu, XB; Zhou, LH; Cheng, JK; Cai, R; Xue, S (2014) SENP1 protects against myocardial ischaemia/reperfusion injury via a HIF1 alpha-dependent pathway. Cardiovasc Res 104:83-92. doi:10.1093/cvr/cvu177

154. Herzog, C; Lorenz, A; Gillmann, HJ; Chowdhury, A; Larmann, J; Harendza, T; Echtermeyer, F; Muller, M; Schmitz, M; Stypmann, J; Seidler, DG; Damm, M; Stehr, SN; Koch, T; Wollert, KC; Conway, EM; Theilmeier, G (2014) Thrombomodulin's lectin-like domain reduces myocardial damage by interfering with HMGB1-mediated TLR2 signalling. Cardiovasc Res 101:400-410. doi:10.1093/cvr/cvt275

155. Inserte, J; Hernando, V; Ruiz-Meana, M; Poncelas-Nozal, M; Fernandez, C; Agullo, L; Sartorio, C; Vilardosa, U; Garcia-Dorado, D (2014) Delayed phospholamban phosphorylation in post-conditioned heart favours Ca2+ normalization and contributes to protection. Cardiovasc Res 103:542-553. doi:10.1093/cvr/cvu163

156. Jiang, BM; Liang, PF; Wang, KK; Lv, CL; Sun, L; Tong, ZY; Liu, YJ; Xiao, XZ (2014) Nucleolin involved in myocardial ischaemic preconditioning via post-transcriptional control of HSPA1A expression. Cardiovasc Res 102:56-67. doi:10.1093/cvr/cvu006

157. Jiang, SX; Streeter, J; Schickling, BM; Zimmerman, K; Weiss, RM; Miller, FJ (2014) Nox1 NADPH oxidase is necessary for late but not early myocardial ischaemic preconditioning. Cardiovasc Res 102:79-87. doi:10.1093/cvr/cvu027

158. Kang, J; Yun, JY; Hur, J; Kang, JA; Choi, JI; Ko, SB; Lee, J; Kim, JY; Hwang, IC; Park, YB; Kim, HS (2014) Erythropoietin priming improves the vasculogenic potential of G-CSF mobilized human peripheral bloodmononuclear cells. Cardiovasc Res 104:171-182. doi:10.1093/cvr/cvu180

159. Li, JY; Chen, XQ; McClusky, R; Ruiz-Sundstrom, M; Itoh, Y; Umar, S; Arnold, AP; Eghbali, M (2014) The number of X chromosomes influences protection from cardiac ischaemia/reperfusion injury in mice: one X is better than two. Cardiovasc Res 102:-. doi:10.1093/cvr/cvu064

160. Lopez-Olaneta, MM; Villalba, M; Gomez-Salinero, JM; Jimenez-Borreguero, LJ; Breckenridge, R; Ortiz-Sanchez, P; Garcia-Pavia, P; Ibanez, B; Lara-Pezzi, E (2014) Induction of the calcineurin variant CnA beta 1 after myocardial infarction reduces post-infarction ventricular remodelling by promoting infarct vascularization. Cardiovasc Res 102:396-406. doi:10.1093/cvr/cvu068

161. Luedde, M; Lutz, M; Carter, N; Sosna, J; Jacoby, C; Vucur, M; Gautheron, J; Roderburg, C; Borg, N; Reisinger, F; Hippe, HJ; Linkermann, A; Wolf, MJ; Rose-John, S; Lullmann-Rauch, R; Adam, D; Flogel, U; Heikenwalder, M; Luedde, T; Frey, N (2014) RIP3, a kinase promoting necroptotic cell death, mediates adverse remodelling after myocardial infarction. Cardiovasc Res 103:206-216. doi:10.1093/cvr/cvu146

162. Morel, S; Braunersreuther, V; Chanson, M; Bouis, D; Rochemont, V; Foglia, B; Pelli, G; Sutter, E; Pinsky, DJ; Mach, F; Kwak, BR (2014) Endothelial Cx40 limits myocardial ischaemia/reperfusion injury in mice. Cardiovasc Res 102:329-337. doi:10.1093/cvr/cvu063

163. Ong, SG; Lee, WH; Theodorou, L; Kodo, K; Lim, SY; Shukla, DH; Briston, T; Kiriakidis, S; Ashcroft, M; Davidson, SM; Maxwell, PH; Yellon, DM; Hausenloy, DJ (2014) HIF-1 reduces ischaemia-reperfusion injury in the heart by targeting the mitochondrial permeability transition pore. Cardiovasc Res 104:24-36. doi:10.1093/cvr/cvu172

164. Pride, CK; Mo, L; Quesnelle, K; Dagda, RK; Murillo, D; Geary, L; Corey, C; Portella, R; Zharikov, S; St Croix, C; Maniar, S; Chu, CT; Khoo, NH; Shiva, S (2014) Nitrite activates protein kinase A in normoxia to mediate mitochondrial fusion and tolerance to ischaemia/reperfusion. Cardiovasc Res 101:57-68. doi:10.1093/cvr/cvt224

165. Saxena, A; Bujak, M; Frunza, O; Dobaczewski, M; Gonzalez-Quesada, C; Lu, B; Gerard, C; Frangogiannis, NG (2014) CXCR3-independent actions of the CXC chemokine CXCL10 in the infarcted myocardium and in isolated cardiac fibroblasts are mediated through proteoglycans. Cardiovasc Res 103:217-227. doi:10.1093/cvr/cvu138

166. Wang, XH; Ha, TZ; Zou, JH; Ren, DY; Liu, L; Zhang, X; Kalbfleisch, J; Gao, X; Williams, D; Li, CF (2014) MicroRNA-125b protects against myocardial ischaemia/reperfusion injury via targeting p53-mediated apoptotic signalling and TRAF6. Cardiovasc Res 102:385-395. doi:10.1093/cvr/cvu044

167. Wu, M; Zhou, JL; Cheng, M; Boriboun, C; Biyashev, D; Wang, H; Mackie, A; Thorne, T; Chou, J; Wu, YP; Chen, ZS; Liu, QH; Yan, HB; Yang, Y; Jie, CF; Tang, YL; Zhao, TC; Taylor, RN; Kishore, R; Losordo, DW; Qin, GJ (2014) E2F1 suppresses cardiac neovascularization by down-regulating VEGF and PlGF expression. Cardiovasc Res 104:412-422. doi:10.1093/cvr/cvu222

168. Wu, W; Hu, YP; Li, JT; Zhu, WN; Ha, TZ; Que, LL; Liu, L; Zhu, Q; Chen, Q; Xu, Y; Li, CF; Li, YH (2014) Silencing of Pellino1 improves post-infarct cardiac dysfunction and attenuates left ventricular remodelling in mice. Cardiovasc Res 102:46-55. doi:10.1093/cvr/cvu007

169. Alburquerque-Bejar, JJ; Barba, I; Inserte, J; Miro-Casas, E; Ruiz-Meana, M; Poncelas, M; Vilardosa, U; Valls-Lacalle, L; Rodriguez-Sinovas, A; Garcia-Dorado, D (2015) Combination therapy with remote ischaemic conditioning and insulin or exenatide enhances infarct size limitation in pigs. Cardiovasc Res 107:246-254. doi:10.1093/cvr/cvv171

170. Andersson, L; Tang, MS; Lundqvist, A; Lindbom, M; Mardani, I; Fogelstrand, P; Shahrouki, P; Redfors, B; Omerovic, E; Levin, M; Boren, J; Levin, MC (2015) Rip2 modifies VEGF-induced signalling and vascular permeability in myocardial ischaemia. Cardiovasc Res 107:478-486. doi:10.1093/cvr/cvv186

171. Bandaru, S; Gronros, J; Redfors, B; Cil, C; Pazooki, D; Salimi, R; Larsson, E; Zhou, AX; Omerovic, E; Akyurek, LM (2015) Deficiency of filamin A in endothelial cells impairs left ventricular remodelling after myocardial infarction. Cardiovasc Res 105:151-159. doi:10.1093/cvr/cvu226

172. Bibli, SI; Andreadou, I; Chatzianastasiou, A; Tzimas, C; Sanoudou, D; Kranias, E; Brouckaert, P; Coletta, C; Szabo, C; KremastiNOS, DT; Iliodromitis, EK; Papapetropoulos, A (2015) Cardioprotection by H2S engages a cGMP-dependent protein kinase G/phospholamban pathway. Cardiovasc Res 106:432-442. doi:10.1093/cvr/cvv129

173. Chen, YX; Liu, JL; Zheng, YJ; Wang, JX; Wang, ZH; Gu, SS; Tan, JL; Jing, Q; Yang, HT (2015) Uncoupling protein 3 mediates H2O2 preconditioning-afforded cardioprotection through the inhibition of MPTP opening. Cardiovasc Res 105:192-202. doi:10.1093/cvr/cvu256

174. Davidson, SM; Foote, K; Kunuthur, S; Gosain, R; Tan, N; Tyser, R; Zhao, YJ; Graeff, R; Ganesan, A; Duchen, MR; Patel, S; Yellon, DM (2015) Inhibition of NAADP signalling on reperfusion protects the heart by preventing lethal calcium oscillations via two-pore channel 1 and opening of the mitochondrial permeability transition pore. Cardiovasc Res 108:357-366. doi:10.1093/cvr/cvv226

175. de Hoog, VC; Bovens, SM; de Jager, SCA; van Middelaar, BJ; van Duijvenvoorde, A; Doevendans, PA; Pasterkamp, G; de Kleijn, DPV; Timmers, L (2015) BLT1 antagonist LSN2792613 reduces infarct size in a mouse model of myocardial ischaemia-reperfusion injury. Cardiovasc Res 108:367-376. doi:10.1093/cvr/cvv224

176. Du, M; Huang, K; Huang, D; Yang, L; Gao, L; Wang, XJ; Huang, DD; Li, XR; Wang, C; Zhang, FX; Wang, Y; Cheng, M; Tong, QS; Qin, GJ; Huang, K; Wang, L (2015) Renalase is a novel target gene of hypoxia-inducible factor-1 in protection against cardiac ischaemia-reperfusion injury. Cardiovasc Res 105:182-191. doi:10.1093/cvr/cvu255

177. Esposito, G; Schiattarella, GG; Perrino, C; Cattaneo, F; Pironti, G; Franzone, A; Gargiulo, G; Magliulo, F; Serino, F; Carotenuto, G; Sannino, A; Ilardi, F; Scudiero, F; Brevetti, L; Oliveti, M; Giugliano, G; Del Giudice, C; Ciccarelli, M; Renzone, G; Scaloni, A; Zambrano, N; Trimarco, B (2015) Dermcidin: a skeletal muscle myokine modulating cardiomyocyte survival and infarct size after coronary artery ligation. Cardiovasc Res 107:431-441. doi:10.1093/cvr/cvv173

178. Lucas, A; Mialet-Perez, J; Daviaud, D; Parini, A; Marber, MS; Sicard, P (2015) Gadd45 gamma regulates cardiomyocyte death and post-myocardial infarction left ventricular remodelling. Cardiovasc Res 108:254-267. doi:10.1093/cvr/cvv219

179. Basalay, MV; Mastitskaya, S; Mrochek, A; Ackland, GL; del Arroyo, AG; Sanchez, J; Sjoquist, PO; Pernow, J; Gourine, AV; Gourine, A (2016) Glucagon-like peptide-1 (GLP-1) mediates cardioprotection by remote ischaemic conditioning. Cardiovasc Res 112:669-676. doi:10.1093/cvr/cvw216

180. Booij, HG; Yu, HJ; De Boer, RA; van de Kolk, CWA; van de Sluis, B; Van Deursen, JM; Van Gilst, WH; Sillje, HHW; Westenbrink, BD (2016) Overexpression of A kinase interacting protein 1 attenuates myocardial ischaemia/reperfusion injury but does not influence heart failure development. Cardiovasc Res 111:217-226. doi:10.1093/cvr/cvw161

181. Eerenberg, ES; Teunissen, PFA; van den Born, BJ; Meijers, JCM; Hollander, MR; Jansen, M; Tijssen, R; Belien, JAM; van de Ven, PM; Aly, MF; Kamp, O; Niessen, HW; Kamphuisen, PW; Levi, M; van Royen, N (2016) The role of ADAMTS13 in acute myocardial infarction: cause or consequence?. Cardiovasc Res 111:194-203. doi:10.1093/cvr/cvw097

182. Gonzalez-Santamaria, J; Villalba, M; Busnadiego, O; Lopez-Olaneta, MM; Sandoval, P; Snabel, J; Lopez-Cabrera, M; Erler, JT; Hanemaaijer, R; Lara-Pezzi, E; Rodriguez-Pascual, F (2016) Matrix cross-linking lysyl oxidases are induced in response to myocardial infarction and promote cardiac dysfunction. Cardiovasc Res 109:67-78. doi:10.1093/cvr/cvv214

183. Hu, ZY; Crump, SM; Zhang, P; Abbott, GW (2016) Kcne2 deletion attenuates acute post-ischaemia/reperfusion myocardial infarction. Cardiovasc Res 110:227-237. doi:10.1093/cvr/cvw048

184. Kan, XY; Wu, YN; Ma, YC; Zhang, CC; Li, P; Wu, LF; Zhang, S; Li, YL; Du, J (2016) Deficiency of IL-12p35 improves cardiac repair after myocardial infarction by promoting angiogenesis. Cardiovasc Res 109:249-259. doi:10.1093/cvr/cvv255

185. Kumagai, S; Nakayama, H; Fujimoto, M; Honda, H; Serada, S; Ishibashi-Ueda, H; Kasai, A; Obana, M; Sakata, Y; Sawa, Y; Fujio, Y; Naka, T (2016) Myeloid cell-derived LRG attenuates adverse cardiac remodelling after myocardial infarction. Cardiovasc Res 109:272-282. doi:10.1093/cvr/cvv273

186. Matteucci, M; Casieri, V; Gabisonia, K; Aquaro, GD; Agostini, S; Pollio, G; Diamanti, D; Rossi, M; Travagli, M; Porcari, V; Recchia, FA; Lionetti, V (2016) Magnetic resonance imaging of infarct-induced canonical wingless/integrated (Wnt)/beta-catenin/T-cell factor pathway activation, in vivo. Cardiovasc Res 112:645-655. doi:10.1093/cvr/cvw214

187. Morel, S; Christoffersen, C; Axelsen, LN; Montecucco, F; Rochemont, V; Frias, MA; Mach, F; James, RW; Naus, CC; Chanson, M; Lampe, PD; Nielsen, MS; Nielsen, LB; Kwak, BR (2016) Sphingosine-1-phosphate reduces ischaemia-reperfusion injury by phosphorylating the gap junction protein Connexin43. Cardiovasc Res 109:385-396. doi:10.1093/cvr/cvw004

188. Sun, JH; Aponte, AM; Menazza, S; Gucek, M; Steenbergen, C; Murphy, E (2016) Additive cardioprotection by pharmacological postconditioning with hydrogen sulfide and nitric oxide donors in mouse heart: S-sulfhydration vs. S-nitrosylation. Cardiovasc Res 110:96-106. doi:10.1093/cvr/cvw037

189. Takada, S; Masaki, Y; Kinugawa, S; Matsumoto, J; Furihata, T; Mizushima, W; Kadoguchi, T; Fukushima, A; Homma, T; Takahashi, M; Harashima, S; Matsushima, S; Yokota, T; Tanaka, S; Okita, K; Tsutsui, H (2016) Dipeptidyl peptidase-4 inhibitor improved exercise capacity and mitochondrial biogenesis in mice with heart failure via activation of glucagon-like peptide-1 receptor signalling. Cardiovasc Res 111:338-347. doi:10.1093/cvr/cvw182

190. Valls-Lacalle, L; Barba, I; Miro-Casas, E; Alburquerque-Bejar, JJ; Ruiz-Meana, M; Fuertes-Agudo, M; Rodriguez-Sinovas, A; Garcia-Dorado, D (2016) Succinate dehydrogenase inhibition with malonate during reperfusion reduces infarct size by preventing mitochondrial permeability transition. Cardiovasc Res 109:374-384. doi:10.1093/cvr/cvv279

191. Alburquerque-Bejar, JJ; Barba, I; Valls-Lacalle, L; Ruiz-Meana, M; Pecoraro, M; Rodriguez-Sinovas, A; Garcia-Dorado, D (2017) Remote ischemic conditioning provides humoural cross-species cardioprotection through glycine receptor activation. Cardiovasc Res 113:52-60. doi:10.1093/cvr/cvw242

192. Bayoumi, AS; Teoh, JP; Aonuma, T; Yuan, ZZ; Ruan, XF; Tang, YL; Su, HB; Weintraub, NL; Kim, IM (2017) MicroRNA-532 protects the heart in acute myocardial infarction, and represses prss23, a positive regulator of endothelial-to-mesenchymal transition. Cardiovasc Res 113:1603-1614. doi:10.1093/cvr/cvx132

193. Bibli, SI; Zhou, ZM; Zukunft, S; Fisslthaler, B; Andreadou, I; Szabo, C; Brouckaert, P; Fleming, I; Papapetropoulos, A (2017) Tyrosine phosphorylation of eNOS regulates myocardial survival after an ischaemic insult: role of PYK2. Cardiovasc Res 113:926-937. doi:10.1093/cvr/cvx058

194. Chen, ZH; Xie, JH; Hao, HX; Lin, HR; Wang, L; Zhang, YX; Chen, L; Cao, SP; Huang, XB; Liao, WJ; Bin, JP; Liao, YL (2017) Ablation of periostin inhibits post-infarction myocardial regeneration in neonatal mice mediated by the phosphatidyliNOSitol 3 kinase/glycogen synthase kinase 3 beta/cyclin D1 signalling pathway. Cardiovasc Res 113:620-632. doi:10.1093/cvr/cvx001

195. Garikipati, VNS; Verma, SK; Jolardarashi, D; Cheng, ZJ; Ibetti, J; Cimini, M; Tang, Y; Khan, M; Yue, YJ; Benedict, C; Nickoloff, E; Truongcao, MM; Gao, EH; Krishnamurthy, P; Goukassian, DA; Koch, WJ; Kishore, R (2017) Therapeutic inhibition of miR-375 attenuates post-myocardial infarction inflammatory response and left ventricular dysfunction via PDK-1-AKT signalling axis. Cardiovasc Res 113:938-949. doi:10.1093/cvr/cvx052

196. Huang, CH; Liu, Y; Beenken, A; Jiang, L; Gao, X; Huang, ZF; Hsu, A; Gross, GJ; Wang, YG; Mohammadi, M; Schultz, JEJ (2017) A novel fibroblast growth factor-1 ligand with reduced heparin binding protects the heart against ischemia-reperfusion injury in the presence of heparin co-administration. Cardiovasc Res 113:1585-1602. doi:10.1093/cvr/cvx165

197. Poncelas, M; Inserte, J; Aluja, D; Hernando, V; Vilardosa, U; Garcia-Dorado, D (2017) Delayed, oral pharmacological inhibition of calpains attenuates adverse post-infarction remodelling. Cardiovasc Res 113:950-961. doi:10.1093/cvr/cvx073

198. Raleigh, JV; Mauro, AG; Devarakonda, T; Marchetti, C; He, J; Kim, E; Filippone, S; Das, A; Toldo, S; Abbate, A; Salloum, FN (2017) Reperfusion therapy with recombinant human relaxin-2

(Serelaxin) attenuates myocardial infarct size and NLRP3 inflammasome following ischemia/reperfusion injury via eNOS-dependent mechanism. Cardiovasc Res 113:609-619. doi:10.1093/cvr/cvw246

199. Ribeiro-Rodrigues, TM; Laundos, TL; Pereira-Carvalho, R; Batista-Almeida, D; Pereira, R; Coelho-Santos, V; Silva, AP; Fernandes, R; Zuzarte, M; Enguita, FJ; Costa, MC; Pinto-do-O, P; Pinto, MT; Gouveia, P; Ferreira, L; Mason, JC; Pereira, P; Kwak, BR; Nascimento, DS; Girao, H (2017) Exosomes secreted by cardiomyocytes subjected to ischaemia promote cardiac angiogenesis. Cardiovasc Res 113:1338-1350. doi:10.1093/cvr/cvx118

200. Vincent, A; Sportouch, C; Covinhes, A; Barrere, C; Gallot, L; Delgado-Betancourt, V; Lattuca, B; Solecki, K; Boisguerin, P; Piot, C; Nargeot, J; Barrere-Lemaire, S (2017) Cardiac mGluR1 metabotropic receptors in cardioprotection. Cardiovasc Res 113:644-655. doi:10.1093/cvr/cvx024

201. Wang, YM; Jin, L; Song, Y; Zhang, M; Shan, D; Liu, YL; Fang, M; Lv, FX; Xiao, RP; Zhang, Y (2017) beta-arrestin 2 mediates cardiac ischemia-reperfusion injury via inhibiting GPCR-independent cell survival signalling. Cardiovasc Res 113:1615-1626. doi:10.1093/cvr/cvx147

202. Wang, ZH; Huang, SY; Sheng, YL; Peng, X; Liu, H; Jin, N; Cai, J; Shu, YW; Li, T; Li, P; Fan, C; Hu, XF; Zhang, WY; Long, R; You, Y; Huang, CH; Song, Y; Xiang, CH; Wang, J; Yang, Y; Liu, K (2017) Topiramate modulates post-infarction inflammation primarily by targeting monocytes ormacrophages. Cardiovasc Res 113:475-487. doi:10.1093/cvr/cvx027

203. Barile, L; Cervio, E; Lionetti, V; Milano, G; Ciullo, A; Biemmi, V; Bolis, S; Altomare, C; Matteucci, M; Di Silvestre, D; Brambilla, F; Fertig, TE; Torre, T; Demertzis, S; Mauri, P; Moccetti, T; Vassalli, G (2018) Cardioprotection by cardiac progenitor cell-secreted exosomes: role of pregnancy-associated plasma protein-A. Cardiovasc Res 114:992-1005. doi:10.1093/cvr/cvy055

204. Chen, CW; Wang, LL; Zaman, S; Gordon, J; Arisi, MF; Venkataraman, CM; Chung, JJ; Hung, G; Gaffey, AC; Spruce, LA; Fazelinia, H; Gorman, RC; Seeholzer, SH; Burdick, JA; Atluri, P (2018) Sustained release of endothelial progenitor cell-derived extracellular vesicles from shear-thinning hydrogels improves angiogenesis and promotes function after myocardial infarction. Cardiovasc Res 114:1029-1040. doi:10.1093/cvr/cvy067

205. Chen, H; Jing, XY; Shen, YJ; Wang, TL; Ou, C; Lu, SF; Cai, Y; Li, Q; Chen, X; Ding, YJ; Yu, XC; Zhu, BM (2018) Stat5-dependent cardioprotection in late remote ischaemia preconditioning. Cardiovasc Res 114:679-689. doi:10.1093/cvr/cvy014

206. Deutsch, MA; Doppler, SA; Li, XH; Lahm, H; Santamaria, G; Cuda, G; Eichhorn, S; Ratschiller, T; Dzilic, E; Dressen, M; Eckart, A; Stark, K; Massberg, S; Bartets, A; Rischpter, C; Gilsbach, R; Hein, L; Fleischmann, BK; Wu, SM; Lange, R; Krane, M (2018) Reactivation of the Nkx2.5 cardiac enhancer aftermyocardial infarction does not presagemyogenesis. Cardiovasc Res 114:1098-1114. doi:10.1093/cvr/cvy069

207. Ding, ZF; Wang, XW; Liu, SJ; Shahanawaz, J; Theus, S; Fan, YB; Deng, XY; Zhou, SC; Mehta, JL (2018) PCSK9 expression in the ischaemic heart and its relationship to infarct size, cardiac function, and development of autophagy. Cardiovasc Res 114:1738-1751. doi:10.1093/cvr/cvy128

208. Francois, AA; Obasanjo-Blackshire, K; Clark, JE; Boguslavskyi, A; Holt, MR; Parker, PJ; Marber, MS; Heads, RJ (2018) Loss of Protein Kinase Novel 1 (PKN1) is associated with mild systolic and diastolic contractile dysfunction, increased phospholamban Thr 17 phosphorylation, and exacerbated ischaemia-reperfusion injury. Cardiovasc Res 114:138-157. doi:10.1093/cvr/cvx206

209. Frankenreiter, S; Groneberg, D; Kuret, A; Krieg, T; Ruth, P; Friebe, A; Lukowski, R (2018) Cardioprotection by ischemic postconditioning and cyclic guanosine monophosphate-elevating agents involves cardiomyocyte nitric oxide-sensitive guanylyl cyclase. Cardiovasc Res 114:822-829. doi:10.1093/cvr/cvy039

210. Ueta, CB; Campos, JC; Albuquerque, RPE; Lima, VM; Disatnik, MH; Sanchez, AB; Chen, CH; de Medeiros, MHG; Yang, WJ; Mochly-Rosen, D; Ferreira, JCB (2018) Cardioprotection induced by a brief exposure to acetaldehyde: role of aldehyde dehydrogenase 2. Cardiovasc Res 114:1006-1015. doi:10.1093/cvr/cvy070

211. Vilahur, G; Gutierrez, M; Casani, L; Lambert, C; Mendieta, G; Ben-Aicha, S; Capdevila, A; Pons-Llado, G; Carreras, F; Carlsson, L; Hidalgo, A; Badimon, L (2018) P2Y(12) antagonists and cardiac repair post-myocardial infarction: global and regional heart function analysis and molecular assessments in pigs. Cardiovasc Res 114:1860-1870. doi:10.1093/cvr/cvy201

212. Wang, L; Quan, NH; Sun, WQ; Chen, X; Cates, C; Rousselle, T; Zhou, XC; Zhao, XZ; Li, J (2018) Cardiomyocyte-specific deletion of Sirt1 gene sensitizes myocardiumto ischaemia and reperfusion injury. Cardiovasc Res 114:805-821. doi:10.1093/cvr/cvy033

213. Whittington, HJ; Ostrowski, PJ; McAndrew, DJ; Cao, F; Shaw, A; Eykyn, TR; Lake, HA; Tyler, J; Schneider, JE; Neubauer, S; Zervou, S; Lygate, CA (2018) Over-expression of mitochondrial creatine kinase in the murine heart improves functional recovery and protects against injury following ischaemia-reperfusion. Cardiovasc Res 114:858-869. doi:10.1093/cvr/cvy054

214. Xia, YL; Zhang, FY; Zhao, SH; Li, YY; Chen, XY; Gao, EH; Xu, XY; Xiong, ZY; Zhang, XM; Zhang, JL; Zhao, HS; Wang, W; Wang, HL; Guo, YJ; Liu, Y; Li, CY; Wang, S; Zhang, L; Yan, WJ; Tao, L (2018) Adiponectin determines farnesoid X receptor agonism-mediated cardioprotection against post-infarction remodelling and dysfunction. Cardiovasc Res 114:1335-1349. doi:10.1093/cvr/cvy093

215. Ziegler, KA; Ahles, A; Wille, T; Kerler, J; Ramanujam, D; Engelhardt, S (2018) Local sympathetic denervation attenuates myocardial inflammation and improves cardiac function after myocardial infarction in mice. Cardiovasc Res 114:291-299. doi:10.1093/cvr/cvx227

216. Bibli, SI; Papapetropoulos, A; Iliodromitis, EK; Daiber, A; Randriamboavonjy, V; Steven, S; Brouckaert, P; Chatzianastasiou, A; Kypreos, KE; Hausenloy, DJ; Fleming, I; Andreadou, I (2019) Nitroglycerine limits infarct size through S-nitrosation of cyclophilin D: a novel mechanism for an old drug. Cardiovasc Res 115:625-636. doi:10.1093/cvr/cvy222

217. Chen, XY; Zhabyeyev, P; Azad, AK; Wang, W; Minerath, RA; DesAulniers, E; Grueter, CE; Murray, AG; Kassiri, Z; Vanhaesebroeck, B; Oudit, GY (2019) Endothelial and cardiomyocyte PI3K beta divergently regulate cardiac remodelling in response to ischaemic injury. Cardiovasc Res 115:1343-1356. doi:10.1093/cvr/cvy298

218. Fujiwara, M; Matoba, T; Koga, JI; Okahara, A; Funamoto, D; Nakano, K; Tsutsui, H; Egashira, K (2019) Nanoparticle incorporating Toll-like receptor 4 inhibitor attenuates myocardial ischaemia-reperfusion injury by inhibiting monocyte-mediated inflammation in mice. Cardiovasc Res 115:1244-1255. doi:10.1093/cvr/cvz066

219. Jones, JL; Peana, D; Veteto, AB; Lambert, MD; Nourian, Z; Karasseva, NG; Hill, MA; Lindman, BR; Baines, CP; Krenz, M; Domeier, TL (2019) TRPV4 increases cardiomyocyte calcium cycling and contractility yet contributes to damage in the aged heart following hypoosmotic stress. Cardiovasc Res 115:46-56. doi:10.1093/cvr/cvy156

220. Kimura, T; Tajiri, K; Sato, A; Sakai, S; Wang, Z; Yoshida, T; Uede, T; Hiroe, M; Aonuma, K; Ieda, M; Imanaka-Yoshida, K (2019) Tenascin-C accelerates adverse ventricular remodelling after myocardial infarction by modulating macrophage polarization. Cardiovasc Res 115:614-624. doi:10.1093/cvr/cvy244

221. Laudette, M; Coluccia, A; Sainte-Marie, Y; Solari, A; Fazal, L; Sicard, P; Silvestri, R; Mialet-Perez, J; Pons, S; Ghaleh, B; Blondeau, JP; Lezoualc'h, F (2019) Identification of a pharmacological inhibitor of Epac1 that protects the heart against acute and chronic models of cardiac stress. Cardiovasc Res 115:1766-1777. doi:10.1093/cvr/cvz076

222. Li, Z; Agrawal, V; Ramratnam, M; Sharma, RK; D'Auria, S; Sincoular, A; Jakubiak, M; Music, ML; Kutschke, WJ; Huang, XN; Gifford, L; Ahmad, F (2019) Cardiac sodium-dependent glucose cotransporter 1 is a novel mediator of ischaemia/reperfusion injury. Cardiovasc Res 115:1646-1658. doi:10.1093/cvr/cvz037

223. Lindsey, ML; Jung, M; Yabluchanskiy, A; Cannon, PL; Iyer, RP; Flynn, ER; DeLeon-Pennell, KY; Valerio, FM; Harrison, CL; Ripplinger, CM; Hall, ME; Ma, YG (2019) Exogenous CXCL4 infusion inhibits macrophage phagocytosis by limiting CD36 signalling to enhance post-myocardial infarction cardiac dilation and mortality. Cardiovasc Res 115:395-408. doi:10.1093/cvr/cvy211

224. Liu, YN; Paterson, M; Baumgardt, SL; Irwin, MG; Xia, ZY; Bosnjak, ZJ; Ge, ZD (2019) Vascular endothelial growth factor regulation of endothelial nitric oxide synthase phosphorylation is involved in isoflurane cardiac preconditioning. Cardiovasc Res 115:168-178. doi:10.1093/cvr/cvy157

225. Luo, H; Li, XH; Li, TZM; Zhao, L; He, JN; Zha, LH; Qi, QQ; Yu, ZX (2019) microRNA-423-3p exosomes derived from cardiac fibroblasts mediates the cardioprotective effects of ischaemic post-conditioning. Cardiovasc Res 115:1189-1204. doi:10.1093/cvr/cvy231

226. Matsumoto, K; Obana, M; Kobayashi, A; Kihara, M; Shioi, G; Miyagawa, S; Maeda, M; Sakata, Y; Nakayama, H; Sawa, Y; Fujio, Y (2019) Blockade of NKG2D/NKG2D ligand interaction attenuated

cardiac remodelling after myocardial infarction. Cardiovasc Res 115:765-775. doi:10.1093/cvr/cvy254

227. Nikolaou, PE; Boengler, K; Efentakis, P; Vouvogiannopoulou, K; Zoga, A; Gaboriaud-Kolar, N; Myrianthopoulos, V; Alexakos, P; Kostomitsopoulos, N; Rerras, I; Tsantili-Kakoulidou, A; Skaltsounis, AL; Papapetropoulos, A; Iliodromitis, EK; Schulz, R; Andreadou, I (2019) Investigating and re-evaluating the role of glycogen synthase kinase 3 beta kinase as a molecular target for cardioprotection by using novel pharmacological inhibitors. Cardiovasc Res 115:1228-1243. doi:10.1093/cvr/cvz061

228. Schloss, MJ; Horckmans, M; Guillamat-Prats, R; Hering, D; Lauer, E; Lenglet, S; Weber, C; Thomas, A; Steffens, S (2019) 2-Arachidonoylglycerol mobilizes myeloid cells and worsens heart function after acute myocardial infarction. Cardiovasc Res 115:602-613. doi:10.1093/cvr/cvy242

229. Surinkaew, S; Aflaki, M; Takawale, A; Chen, Y; Qi, XY; Gillis, MA; Shi, YF; Tardif, JC; Chattipakorn, N; Nattel, S (2019) Exchange protein activated by cyclic-adeNOSine monophosphate (Epac) regulates atrial fibroblast function and controls cardiac remodelling. Cardiovasc Res 115:94-106. doi:10.1093/cvr/cvy173

230. Tokutome, M; Matoba, T; Nakano, Y; Okahara, A; Fujiwara, M; Koga, JI; Nakano, K; Tsutsui, H; Egashira, K (2019) Peroxisome proliferator-activated receptor-gamma targeting nanomedicine promotes cardiac healing after acute myocardial infarction by skewing monocyte/macrophage polarization in preclinical animal models. Cardiovasc Res 115:419-431. doi:10.1093/cvr/cvy200

231. Uthman, L; Nederlof, R; Eerbeek, O; Baartscheer, A; Schumacher, C; Buchholtz, N; Hollmann, MW; Coronel, R; Weber, NC; Zuurbier, CJ (2019) Delayed ischaemic contracture onset by empagliflozin associates with NHE1 inhibition and is dependent on insulin in isolated mouse hearts. Cardiovasc Res 115:1533-1545. doi:10.1093/cvr/cvz004

232. Valverde, CA; Mazzocchi, G; Di Carlo, MN; Pardo, AC; Salas, N; Ragone, MI; Felice, JI; Cely-Ortiz, A; Consolini, AE; Portiansky, E; Mosca, S; Kranias, EG; Wehrens, XHT; Mattiazzi, A (2019) Ablation of phosphotamban rescues reperfusion arrhythmias but exacerbates myocardium infarction in hearts with Ca2+/calmodulin kinase II constitutive phosphorylation of ryanodine receptors. Cardiovasc Res 115:556-569. doi:10.1093/cvr/cvy213

233. Wang, YN; Liu, JL; Kong, QY; Cheng, H; Tu, F; Yu, P; Liu, Y; Zhang, XJ; Li, CF; Li, YH; Min, XX; Du, SY; Ding, ZN; Liu, L (2019) Cardiomyocyte-specific deficiency of HSPB1 worsens cardiac dysfunction by activating NFB-mediated leucocyte recruitment after myocardial infarction. Cardiovasc Res 115:154-167. doi:10.1093/cvr/cvy163

234. Yeang, C; Hasanally, D; Que, XC; Hung, MY; Stamenkovic, A; Chan, D; Chaudhary, R; Margulets, V; Edel, AL; Hoshijima, M; Gu, YS; Bradford, W; Dalton, N; Miu, P; Cheung, DY; Jassal, DS; Pierce, GN; Peterson, KL; Kirshenbaum, LA; Witztum, JL; Tsimikas, S; Ravandi, A (2019) Reduction of myocardial ischaemia-reperfusion injury by inactivating oxidized phospholipids. Cardiovasc Res 115:179-189. doi:10.1093/cvr/cvy136

235. Zhao, JX; Li, XL; Hu, JX; Chen, F; Qiao, SH; Sun, X; Gao, L; Xie, J; Xu, B (2019) Mesenchymal stromal cell-derived exosomes attenuatemyocardial ischaemia-reperfusion injury throughmiR-182-regulated macrophage polarization. Cardiovasc Res 115:1205-1216. doi:10.1093/cvr/cvz040

236. Boisguerin, P; Covinhes, A; Gallot, L; Barrere, C; Vincent, A; Busson, M; Piot, C; Nargeot, J; Lebleu, B; Barrere-Lemaire, S (2020) A novel therapeutic peptide targeting myocardial reperfusion injury. Cardiovasc Res 116:633-644. doi:10.1093/cvr/cvz145

237. Gao, C; Wang, RT; Li, B; Guo, YZ; Yin, T; Xia, YL; Zhang, FY; Lian, K; Liu, Y; Wang, H; Zhang, L; Gao, EH; Yan, WJ; Tao, L (2020) TXNIP/Redd1 signalling and excessive autophagy: a novel mechanism of myocardial ischaemia/reperfusion injury in mice. Cardiovasc Res 116:645-657. doi:10.1093/cvr/cvz152

238. Huang, PS; Wang, L; Li, Q; Tian, XQ; Xu, J; Xu, JY; Xiong, YY; Chen, GH; Qian, HY; Jin, C; Yu, Y; Cheng, K; Qian, L; Yang, YJ (2020) Atorvastatin enhances the therapeutic efficacy of mesenchymal stem cells-derived exosomes in acute myocardial infarction via up-regulating long non-coding RNA H19. Cardiovasc Res 116:353-367. doi:10.1093/cvr/cvz139

239. Lou, X; Zhao, M; Fan, CM; Fast, VG; Valarmathi, MT; Zhu, WQ; Zhang, JY (2020) N-cadherin overexpression enhances the reparative potency of human-induced pluripotent stem cell-derived cardiac myocytes in infarcted mouse hearts. Cardiovasc Res 116:671-685. doi:10.1093/cvr/cvz179

240. Nguyen, QT; Nsaibia, MJ; Sirois, MG; Calderone, A; Tardif, JC; Shi, YF; Ruiz, M; Daneault, C; Gagnon, L; Grouix, B; Laurin, P; Dupuis, J (2020) PBI-4050 reduces pulmonary hypertension, lung

fibrosis, and right ventricular dysfunction in heart failure. Cardiovasc Res 116:171-182. doi:10.1093/cvr/cvz034

241. Samidurai, A; Roh, SK; Prakash, M; Durrant, D; Salloum, FN; Kukreja, RC; Das, A (2020) STAT3-miR-17/20 signalling axis plays a critical role in attenuating myocardial infarction following rapamycin treatment in diabetic mice. Cardiovasc Res 116:2103-2115. doi:10.1093/cvr/cvz315

242. Schwach, V; Fernandes, MG; Maas, S; Gerhardt, S; Tsonaka, R; van der Weerd, L; Passier, R; Mummery, CL; Birket, MJ; Salvatori, DCF (2020) Expandable human cardiovascular progenitors from stem cells for regenerating mouse heart after myocardial infarction. Cardiovasc Res 116:545-553. doi:10.1093/cvr/cvz181

243. Yang, CF; Chen, YY; Singh, JP; Hsu, SF; Liu, YW; Yang, CY; Chang, CW; Chen, SN; Shih, RH; Hsu, ST; Jou, YS; Cheng, CF; Meng, TC (2020) Targeting protein tyrosine phosphatase PTP-PEST (PTPN12) for therapeutic intervention in acute myocardial infarction. Cardiovasc Res 116:1032-1046. doi:10.1093/cvr/cvz165

244. Amanakis, G; Sun, JH; Fergusson, MM; McGinty, S; Liu, CY; Molkentin, JD; Murphy, E (2021) Cysteine 202 of cyclophilin D is a site of multiple post-translational modifications and plays a role in cardioprotection. Cardiovasc Res 117:212-223. doi:10.1093/cvr/cvaa053

245. Kompa, AR; Greening, DW; Kong, AM; McMillan, PJ; Fang, HY; Saxena, R; Wong, RCB; Lees, JG; Sivakumaran, P; Newcomb, AE; Tannous, BA; Kos, C; Mariana, L; Loudovaris, T; Hausenloy, DJ; Lim, SY (2021) Sustained subcutaneous delivery of secretome of human cardiac stem cells promotes cardiac repair following myocardial infarction. Cardiovasc Res 117:918-929. doi:10.1093/cvr/cvaa088

246. Kuang, YS; Li, XL; Liu, XX; Wei, L; Chen, XL; Liu, J; Zhuang, T; Pi, JJ; Wang, YF; Zhu, CY; Gong, X; Hu, H; Yu, ZR; Li, JM; Yu, P; Fan, HM; Zhang, YZ; Liu, ZM; Zhang, L (2021) Vascular endothelial S1pr1 ameliorates adverse cardiac remodelling via stimulating reparative macrophage proliferation after myocardial infarction. Cardiovasc Res 117:585-599. doi:10.1093/cvr/cvaa046

247. Nambu, H; Takada, S; Maekawa, S; Matsumoto, J; Kakutani, N; Furihata, T; Shirakawa, R; Katayama, T; Nakajima, T; Yamanashi, K; Obata, Y; Nakano, I; Tsuda, M; Saito, A; Fukushima, A; Yokota, T; Nio-Kobayashi, J; Yasui, H; Higashikawa, K; Kuge, Y; Anzai, T; Sabe, H; Kinugawa, S (2021) Inhibition of xanthine oxidase in the acute phase of myocardial infarction prevents skeletal muscle abnormalities and exercise intolerance. Cardiovasc Res 117:805-819. doi:10.1093/cvr/cvaa127

248. Tao, ZH; Loo, SJ; Su, LP; Tan, SH; Tee, GZ; Gan, SU; Zhang, JY; Chen, X; Ye, L (2021) Angiopoietin-1 enhanced myocytemitosis, engraftment, and the reparability of hiPSC-CMs for treatment of myocardial infarction. Cardiovasc Res 117:1578-1591. doi:10.1093/cvr/cvaa215

249. Ambrosini, S; Montecucco, F; Kolijn, D; Pedicino, D; Akhmedov, A; Mohammed, SA; Herwig, M; Gorica, E; Szabo, PL; Weber, L; Russo, G; Vinci, R; Matter, CM; Liuzzo, G; Brown, PJ; Rossi, FMV; Camici, GG; Sciarretta, S; Beltrami, AP; Crea, F; Podesser, B; Luscher, TF; Kiss, A; Ruschitzka, F; Hamdani, N; Costantino, S; Paneni, F (2022) Methylation of the Hippo effector YAP by the methyltransferase SETD7 drives myocardial ischaemic injury: a translational study. Cardiovasc Res 118:3374-3385. doi:10.1093/cvr/cvac102

250. de Lucia, C; Grisanti, LA; Borghetti, G; Piedepalumbo, M; Ibetti, J; Lucchese, AM; Barr, EW; Roy, R; Okyere, AD; Murphy, HC; Gao, E; Rengo, G; Houser, SR; Tilley, DG; Koch, WJ (2022) G protein-coupled receptor kinase 5 (GRK5) contributes to impaired cardiac function and immune cell recruitment in post-ischemic heart failure. Cardiovasc Res 118:169-183. doi:10.1093/cvr/cvab044

251. Dehghani, T; Thai, PN; Sodhi, H; Ren, L; Sirish, P; Nader, CE; Timofeyev, V; Overton, JL; Li, XC; Lam, KS; Chiamvimonvat, N; Panitch, A (2022) Selectin-targeting glycosaminoglycan-peptide conjugate limits neutrophil-mediated cardiac reperfusion injury. Cardiovasc Res 118:267-281. doi:10.1093/cvr/cvaa312

252. del Campo, CV; Liaw, NY; Gunadasa-Rohling, M; Matthaei, M; Braga, L; Kennedy, T; Salinas, G; Voigt, N; Giacca, M; Zimmermann, WH; Riley, PR (2022) Regenerative potential of epicardium-derived extracellular vesicles mediated by conserved miRNA transfer. Cardiovasc Res 118:597-611. doi:10.1093/cvr/cvab054

253. Harris, M; Sun, JH; Keeran, K; Aponte, A; Singh, K; Springer, D; Gucek, M; Pirooznia, M; Cockman, ME; Murphy, E; Kennedy, LM (2022) Ogfod1 deletion increases cardiac beta-alanine levels and protects mice against ischaemia- reperfusion injury. Cardiovasc Res 118:2847-2858. doi:10.1093/cvr/cvab323

254. He, WH; McCarroll, CS; Nather, K; Ford, K; Mangion, K; Riddell, A; O'Toole, D; Zaeri, A; Corcoran, D; Carrick, D; Lee, MMY; McEntegart, M; Davie, A; Good, R; Lindsay, MM; Eteiba, H; Rocchiccioli, P; Watkins, S; Hood, S; Shaukat, A; McArthur, L; Elliott, EB; McClure, J; Hawksby, C; Martin, T; Petrie, MC; Oldroyd, KG; Smith, GL; Channon, KM; Berry, C; Nicklin, SA; Loughrey, CM (2022) Inhibition of myocardial cathepsin-L release during reperfusion following myocardial infarction improves cardiac function and reduces infarct size. Cardiovasc Res 118:1535-1547. doi:10.1093/cvr/cvab204

255. Hubert, F; Payan, SM; Pelce, E; Bouchard, L; Sturny, R; Lenfant, N; Mottola, G; Collart, F; Kelly, RG; Rochais, F (2022) FGF10 promotes cardiac repair through a dual cellular mechanism increasing cardiomyocyte renewal and inhibiting fibrosis. Cardiovasc Res 118:2625-2637. doi:10.1093/cvr/cvab340

256. Kalkhoran, SB; Kriston-Vizi, J; Hernandez-Resendiz, S; Crespo-Avilan, GE; Rosdah, AA; Lees, JG; Da Costa, JRS; Ling, NXY; Holien, JK; Samangouei, P; Chinda, K; Yap, EP; Riquelme, JA; Ketteler, R; Yellon, DM; Lim, SY; Hausenloy, DJ (2022) Hydralazine protects the heart against acute ischaemia/reperfusion injury by inhibiting Drp1-mediated mitochondrial fission. Cardiovasc Res 118:282-294. doi:10.1093/cvr/cvaa343

257. Mia, MM; Cibi, DM; Ghani, SABA; Singh, A; Tee, N; Sivakumar, V; Bogireddi, H; Cook, SA; Mao, JH; Singh, MK (2022) Loss of Yap/Taz in cardiac fibroblasts attenuates adverse remodelling and improves cardiac function. Cardiovasc Res 118:1785-1804. doi:10.1093/cvr/cvab205

258. Shah, M; He, ZH; Rauf, A; Kalkhoran, SB; Heiestad, CM; Stenslokken, KO; Parish, CR; Soehnlein, O; Arjun, S; Davidson, SM; Yellon, D (2022) Extracellular histones are a target in myocardial ischaemia-reperfusion injury. Cardiovasc Res 118:1115-1125. doi:10.1093/cvr/cvab139

259. Xu, JY; Xiong, YY; Tang, RJ; Jiang, WY; Ning, Y; Gong, ZT; Huang, PS; Chen, GH; Xu, J; Wu, CX; Hu, MJ; Xu, J; Xu, Y; Huang, CR; Jin, C; Lu, XT; Qian, HY; Li, XD; Yang, YJ (2022) Interleukin-5-induced eosinophil population improves cardiac function after myocardial infarction. Cardiovasc Res 118:2165-2178. doi:10.1093/cvr/cvab237

260. Akbar, N; Braithwaite, AT; Corr, EM; Koelwyn, GJ; van Solingen, C; Cochain, C; Saliba, AE; Corbin, A; Pezzolla, D; Jorgensen, MM; Baek, R; Edgar, L; De Villiers, C; Gunadasa-Rohling, M; Banerjee, A; Paget, D; Lee, C; Hogg, E; Costin, A; Dhaliwal, R; Johnson, E; Krausgruber, T; Riepsaame, J; Melling, GE; Shanmuganathan, M; Bock, C; Carter, DRF; Channon, KM; Riley, PR; Udalova, IA; Moore, KJ; Anthony, D; Choudhury, RP (2023) Rapid neutrophil mobilization by VCAM-1+endothelial cell-derived extracellular vesicles. Cardiovasc Res 119:236-251. doi:10.1093/cvr/cvac012

261. Berg, J; Jablonowski, R; Nordlund, D; Ryd, D; Heiberg, E; Carlsson, M; Arheden, H (2023) Mild hypothermia attenuates ischaemia/reperfusion injury: insights from serial non-invasive pressure-volume loops. Cardiovasc Res :2230-2243. doi:10.1093/cvr/cvad028

262. Chung, BM; Wang, Y; Thiel, M; Rostami, F; Rogoll, A; Hirsch, VG; Malik, Z; Bührke, A; Bär, C; Klintschar, M; Schmitto, JD; Vogt, C; Werlein, C; Jonigk, D; Bauersachs, J; Wollert, KC; Kempf, T (2023) Pre-emptive iron supplementation prevents myocardial iron deficiency and attenuates adverse remodelling after myocardial infarction. Cardiovasc Res 119:1969-1980. doi:10.1093/cvr/cvad092

263. Dong, M; Chen, DS; Zhu, YX; Yang, S; Kumar, S; Zhang, R; Zhou, Y; Yang, ZY; Zheng, N; Zhu, T; Xiang, JQ; Liu, Y; Kang, L; Liu, J (2023) Impaired regulation of MMP2/16-MLCK3 by miR-146a-5p increased susceptibility to myocardial ischaemic injury in aging mice. Cardiovasc Res 119:786-801. doi:10.1093/cvr/cvac104

264. Hiram, R; Xiong, F; Naud, P; Xiao, JN; Sosnowski, DK; Le Quilliec, E; Saljic, A; Abu-Taha, IH; Kamler, M; Leblanc, CA; Al-U'Datt, DGF; Sirois, MG; Hebert, TE; Tanguay, JF; Tardif, JC; Dobrev, D; Nattel, S (2023) An inflammation resolution-promoting intervention prevents atrial fibrillation caused by left ventricular dysfunction. Cardiovasc Res :-. doi:10.1093/cvr/cvad175

265. Kleinbongard, P; Lieder, H; Skyschally, A; Heusch, G (2023) No sex-related differences in infarct size, no-reflow, and protection by ischaemic pre-conditioning in Gottingen minipigs. Cardiovasc Res 119:561-570. doi:10.1093/cvr/cvac062

266. Kroetsch, JT; Lidington, D; Alibhai, FJ; Reitz, CJ; Zhang, HJ; Dinh, DD; Hanchard, J; Khatua, TN; Heximer, SP; Martino, TA; Bolz, SS (2023) Disrupting circadian control of peripheral myogenic reactivity mitigates cardiac injury following myocardial infarction. Cardiovasc Res 119:1403-1415. doi:10.1093/cvr/cvac174

267. Li, ZY; Dai, RL; Chen, M; Huang, LX; Zhu, K; Li, MY; Zhu, WT; Li, Y; Xie, N; Li, JC; Wang, L; Lan, F; Cao, CM (2023) p55 gamma degrades RIP3 via MG53 to suppress ischaemia-induced myocardial necroptosis and mediates cardioprotection of preconditioning. Cardiovasc Res :2421-2440. doi:10.1093/cvr/cvad123

268. Liu, TX; Meng, ZJ; Liu, J; Li, J; Zhang, YY; Deng, ZY; Luo, SY; Wang, MJ; Huang, Q; Zhang, SY; Fendt, P; Devouassoux, J; Li, DZ; McKenzie, ANJ; Nahrendorf, M; Libby, P; Guo, JL; Shi, GP (2023) Group 2 innate lymphoid cells protect mouse heart from myocardial infarction injury via interleukin 5, eosinophils, and dendritic cells. Cardiovasc Res 119:1046-1061. doi:10.1093/cvr/cvac144

269. Lou, X; Tang, YW; Ye, L; Pretorius, D; Fast, VG; Kahn-Krell, AM; Zhang, J; Zhang, JH; Qiao, AJ; Qin, GJ; Kamp, T; Thomson, JA; Zhang, JY (2023) Cardiac muscle patches containing four types of cardiac cells derived from human pluripotent stem cells improve recovery from cardiac injury in mice. Cardiovasc Res 119:1062-1076. doi:10.1093/cvr/cvad004

270. Rusiecka, OM; Molica, F; Nielsen, MS; Tollance, A; Morel, S; Frieden, M; Chanson, M; Boengler, K; Kwak, BR (2023) Mitochondrial pannexin1 controls cardiac sensitivity to ischaemia/reperfusion injury. Cardiovasc Res :2342-2354. doi:10.1093/cvr/cvad120

271. Sayour, NV; Brenner, GB; Makkos, A; Kiss, B; Kovacshazi, C; Gergely, TG; Aukrust, SG; Tian, HM; Zenkl, V; Gomori, K; Szabados, T; Bencsik, P; Heinen, A; Schulz, R; Baxter, GF; Zuurbier, CJ; Voko, Z; Ferdinandy, P; Giricz, Z (2023) Cardioprotective efficacy of limb remote ischaemic preconditioning in rats: discrepancy between a meta-analysis and a three-centre in vivo study. Cardiovasc Res 119:1336-1351. doi:10.1093/cvr/cvad024

272. Wang, XY; Gaur, M; Mounzih, K; Rodriguez, HJ; Qiu, HL; Chen, M; Yan, LQ; Cooper, BA; Narayan, S; Derakhshandeh, R; Rao, PA; Han, DD; Nabavizadeh, P; Springer, ML; John, CM (2023) Inhibition of galectin-3 post-infarction impedes progressive fibrosis by regulating inflammatory profibrotic cascades. Cardiovasc Res :2536-2549. doi:10.1093/cvr/cvad116

273. Zhao, JX; Zhang, Q; Cheng, W; Dai, Q; Wei, ZH; Guo, M; Chen, F; Qiao, SH; Hu, JX; Wang, JZ; Chen, HT; Bao, X; Mu, D; Sun, X; Xu, B; Xie, J (2023) Heart-gut microbiota communication determines the severity of cardiac injury after myocardial ischaemia/reperfusion. Cardiovasc Res 119:1390-1402. doi:10.1093/cvr/cvad023

274. Bonner, F; Borg, N; Jacoby, C; Temme, S; Ding, ZP; Flogel, U; Schrader, J (2013) Ecto-5-Nucleotidase on Immune Cells Protects From Adverse Cardiac Remodeling. Circ Res 113:301-312. doi:10.1161/CIRCRESAHA.113.300180

275. Duran, JM; Makarewich, CA; Sharp, TE; Starosta, T; Zhu, F; Hoffman, NE; Chiba, Y; Madesh, M; Berretta, RM; Kubo, H; Houser, SR (2013) Bone-Derived Stem Cells Repair the Heart After Myocardial Infarction Through Transdifferentiation and Paracrine Signaling Mechanisms. Circ Res 113:539-552. doi:10.1161/CIRCRESAHA.113.301202

276. Houtgraaf, JH; de Jong, R; Kazemi, K; de Groot, D; van der Spoel, TIG; Arslan, F; Hoefer, I; Pasterkamp, G; Itescu, S; Zijlstra, F; Geleijnse, ML; Serruys, PW; Duckers, HJ (2013) Intracoronary Infusion of Allogeneic Mesenchymal Precursor Cells Directly After Experimental Acute Myocardial Infarction Reduces Infarct Size, Abrogates Adverse Remodeling, and Improves Cardiac Function. Circ Res 113:153-166. doi:10.1161/CIRCRESAHA.112.300730

277. Icli, B; Wara, AKM; Moslehi, J; Sun, XH; Plovie, E; Cahill, M; Marchini, JF; Schissler, A; Padera, RF; Shi, JR; Cheng, HW; Raghuram, S; Arany, Z; Liao, R; Croce, K; MacRae, C; Feinberg, MW (2013) MicroRNA-26a Regulates Pathological and Physiological Angiogenesis by Targeting BMP/SMAD1 Signaling. Circ Res 113:1231-1241. doi:10.1161/CIRCRESAHA.113.301780

278. Lam, CK; Zhao, W; Cai, WF; Vafiadaki, E; Florea, SM; Ren, XP; Liu, Y; Robbins, N; Zhang, ZG; Zhou, XY; Jiang, M; Rubinstein, J; Jones, WK; Kranias, EG (2013) Novel Role of HAX-1 in Ischemic Injury Protection Involvement of Heat Shock Protein 90. Circ Res 112:79-89. doi:10.1161/CIRCRESAHA.112.279935

279. Ling, HY; Gray, CBB; Zambon, AC; Grimm, M; Gu, YS; Dalton, N; Purcell, NH; Peterson, K; Brown, JH (2013) Ca2+/Calmodulin-Dependent Protein Kinase II delta Mediates Myocardial Ischemia/Reperfusion Injury Through Nuclear Factor-kappa B. Circ Res 112:935-944. doi:10.1161/CIRCRESAHA.112.276915

280. Lygate, CA; Aksentijevic, D; Dawson, D; ten Hove, M; Phillips, D; de Bono, JP; Medway, DJ; Sebag-Montefiore, L; Hunyor, I; Channon, KM; Clarke, K; Zervou, S; Watkins, H; Balaban, RS; Neubauer, S (2013) Living Without Creatine Unchanged Exercise Capacity and Response to Chronic

Myocardial Infarction in Creatine-Deficient Mice. Circ Res 112:945-955. doi:10.1161/CIRCRESAHA.112.300725

281. Ma, YG; Halade, GV; Zhang, JH; Ramirez, TA; Levin, D; Voorhees, A; Jin, YF; Han, HC; Manicone, AM; Lindsey, ML (2013) Matrix Metalloproteinase-28 Deletion Exacerbates Cardiac Dysfunction and Rupture After Myocardial Infarction in Mice by Inhibiting M2 Macrophage Activation. Circ Res 112:675-688. doi:10.1161/CIRCRESAHA.111.300502

282. Matsushima, S; Kuroda, J; Ago, T; Zhai, PY; Ikeda, Y; Oka, S; Fong, GH; Tian, R; Sadoshima, J (2013) Broad Suppression of NADPH Oxidase Activity Exacerbates Ischemia/Reperfusion Injury Through Inadvertent Downregulation of Hypoxia-inducible Factor-1 alpha and Upregulation of Peroxisome Proliferator-activated Receptor-alpha. Circ Res 112:1135-1149. doi:10.1161/CIRCRESAHA.111.300171

283. Sciarretta, S; Zhai, PY; Shao, D; Zablocki, D; Nagarajan, N; Terada, LS; Volpe, M; Sadoshima, J (2013) Activation of NADPH Oxidase 4 in the Endoplasmic Reticulum Promotes Cardiomyocyte Autophagy and Survival During Energy Stress Through the Protein Kinase RNA-Activated-Like Endoplasmic Reticulum Kinase/Eukaryotic Initiation Factor 2 alpha/Activating Transcription Factor 4 Pathway. Circ Res 113:1253-1264. doi:10.1161/CIRCRESAHA.113.301787

284. Alibhai, FJ; Tsimakouridze, EV; Chinnappareddy, N; Wright, DC; Billia, F; O'Sullivan, ML; Pyle, WG; Sole, MJ; Martino, TA (2014) Short-Term Disruption of Diurnal Rhythms After Murine Myocardial Infarction Adversely Affects Long-Term Myocardial Structure and Function. Circ Res 114:1713-1722. doi:10.1161/CIRCRESAHA.114.302995

285. Duran, JM; Makarewich, CA; Trappanese, D; Gross, P; Husain, S; Dunn, J; Lal, H; Sharp, TE; Starosta, T; Vagnozzi, RJ; Berretta, RM; Barbe, M; Yu, DH; Gao, E; Kubo, H; Force, T; Houser, SR (2014) Sorafenib Cardiotoxicity Increases Mortality After Myocardial Infarction. Circ Res 114:1700-1712. doi:10.1161/CIRCRESAHA.114.303200

286. Felician, G; Collesi, C; Lusic, M; Martinelli, V; Dal Ferro, M; Zentilin, L; Zacchigna, S; Giacca, M (2014) Epigenetic Modification at Notch Responsive Promoters Blunts Efficacy of Inducing Notch Pathway Reactivation After Myocardial Infarction. Circ Res 115:636-649. doi:10.1161/CIRCRESAHA.115.304517

287. Lin, ZQ; von Gise, A; Zhou, PZ; Gu, F; Ma, Q; Jiang, JM; Yau, AL; Buck, JN; Gouin, KA; van Gorp, PRR; Zhou, B; Chen, JH; Seidman, JG; Wang, DZ; Pu, WT (2014) Cardiac-Specific YAP Activation Improves Cardiac Function and Survival in an Experimental Murine MI Model. Circ Res 115:354-363. doi:10.1161/CIRCRESAHA.115.303632

288. Mishina, H; Watanabe, K; Tamaru, S; Watanabe, Y; Fujioka, D; Takahashi, S; Suzuki, K; Nakamura, T; Obata, J; Kawabata, K; Yokota, Y; Inoue, O; Murakami, M; Hanasaki, K; Kugiyama, K (2014) Lack of Phospholipase A(2) Receptor Increases Susceptibility to Cardiac Rupture After Myocardial Infarction. Circ Res 114:493-504. doi:10.1161/CIRCRESAHA.114.302319

289. Rainer, PP; Hao, S; Vanhoutte, D; Lee, DI; Koitabashi, N; Molkentin, JD; Kass, DA (2014) Cardiomyocyte-Specific Transforming Growth Factor Suppression Blocks Neutrophil Infiltration, Augments Multiple Cytoprotective Cascades, and Reduces Early Mortality After Myocardial Infarction. Circ Res 114:1246-1257. doi:10.1161/CIRCRESAHA.114.302653

290. Rassaf, T; Totzeck, M; Hendgen-Cotta, UB; Shiva, S; Heusch, G; Kelm, M (2014) Circulating Nitrite Contributes to Cardioprotection by Remote Ischemic Preconditioning. Circ Res 114:1601-1610. doi:10.1161/CIRCRESAHA.114.303822

291. Weirather, J; Hofmann, UDW; Beyersdorf, N; Ramos, GC; Vogel, B; Frey, A; Ertl, G; Kerkau, T; Frantz, S (2014) Foxp(3+) CD4+ T Cells Improve Healing After Myocardial Infarction by Modulating Monocyte/Macrophage Differentiation. Circ Res 115:55-67. doi:10.1161/CIRCRESAHA.115.303895

292. Yan, XX; Hegab, AE; Endo, J; Anzai, A; Matsuhashi, T; Katsumata, Y; Ito, K; Yamamoto, T; Betsuyaku, T; Shinmura, K; Shen, WF; Vivier, E; Fukuda, K; Sano, M (2014) Lung Natural Killer Cells Play a Major Counter-Regulatory Role in Pulmonary Vascular Hyperpermeability After Myocardial Infarction. Circ Res 114:637-649. doi:10.1161/CIRCRESAHA.114.302625

293. Yano, T; Ferlito, M; Aponte, A; Kuno, A; Miura, T; Murphy, E; Steenbergen, C (2014) Pivotal Role of mTORC2 and Involvement of Ribosomal Protein S6 in Cardioprotective Signaling. Circ Res 114:1268-1280. doi:10.1161/CIRCRESAHA.114.303562

294. Zavadzkas, JA; Stroud, RE; Bouges, S; Mukherjee, R; Jones, JR; Patel, RK; McDermott, PJ; Spinale, FG (2014) Targeted Overexpression of Tissue Inhibitor of Matrix Metalloproteinase-4

Modifies Post-Myocardial Infarction Remodeling in Mice. Circ Res 114:1435-1445. doi:10.1161/CIRCRESAHA.114.303634

295. Avolio, E; Meloni, M; Spencer, HL; Riu, F; Katare, R; Mangialardi, G; Oikawa, A; Rodriguez-Arabaolaza, I; Dang, ZX; Mitchell, K; Reni, C; Alvino, VV; Rowlinson, J; Livi, U; Cesselli, D; Angelini, G; Emanueli, C; Beltrami, AP; Madeddu, P (2015) Combined Intramyocardial Delivery of Human Pericytes and Cardiac Stem Cells Additively Improves the Healing of Mouse Infarcted Hearts Through Stimulation of Vascular and Muscular Repair. Circ Res 116:E81-E94. doi:10.1161/CIRCRESAHA.115.306146

296. Conklin, DJ; Guo, YR; Jagatheesan, G; Kilfoil, PJ; Haberzettl, P; Hill, BG; Baba, SP; Guo, LP; Wetzelberger, K; Obal, D; Rokosh, DG; Prough, RA; Prabhu, SD; Velayutham, M; Zweier, JL; Hoetker, JD; Riggs, DW; Srivastava, S; Bolli, R; Bhatnagar, A (2015) Genetic Deficiency of Glutathione S-Transferase P Increases Myocardial Sensitivity to Ischemia-Reperfusion Injury. Circ Res 117:437-449. doi:10.1161/CIRCRESAHA.114.305518

297. Ikeda, Y; Shirakabe, A; Maejima, Y; Zhai, PY; Sciarretta, S; Toli, J; Nomura, M; Mihara, K; Egashira, K; Ohishi, M; Abdellatif, M; Sadoshima, J (2015) Endogenous Drp1 Mediates Mitochondrial Autophagy and Protects the Heart Against Energy Stress. Circ Res 116:264-278. doi:10.1161/CIRCRESAHA.116.303356

298. Jayawardena, TM; Finch, EA; Zhang, LN; Zhang, HT; Hodgkinson, CP; Pratt, RE; Rosenberg, PB; Mirotsou, M; Dzau, VJ (2015) MicroRNA Induced Cardiac Reprogramming In Vivo Evidence for Mature Cardiac Myocytes and Improved Cardiac Function. Circ Res 116:418-424. doi:10.1161/CIRCRESAHA.116.304510

299. Jones, SP; Tang, XL; Guo, YR; Steenbergen, C; Lefer, DJ; Kukreja, RC; Kong, MY; Li, QH; Bhushan, S; Zhu, XP; Du, JJ; Nong, YB; Stowers, HL; Kondo, K; Hunt, GN; Goodchild, TT; Orr, A; Chang, CC; Ockaili, R; Salloum, FN; Bolli, R (2015) The NHLBI-Sponsored Consortium for preclinicAl assESsment of cARdioprotective Therapies (CAESAR) A New Paradigm for Rigorous, Accurate, and Reproducible Evaluation of Putative Infarct-Sparing Interventions in Mice, Rabbits, and Pigs. Circ Res 116:572-586. doi:10.1161/CIRCRESAHA.116.305462

300. Khan, M; Nickoloff, E; Abramova, T; Johnson, J; Verma, SK; Krishnamurthy, P; Mackie, AR; Vaughan, E; Garikipati, VNS; Benedict, C; Ramirez, V; Lambers, E; Ito, A; Gao, E; Misener, S; Luongo, T; Elrod, J; Qin, GJ; Houser, SR; Koch, WJ; Kishore, R (2015) Embryonic Stem Cell-Derived Exosomes Promote Endogenous Repair Mechanisms and Enhance Cardiac Function Following Myocardial Infarction. Circ Res 117:52-64. doi:10.1161/CIRCRESAHA.117.305990

301. Kim, PJ; Mahmoudi, M; Ge, XH; Matsuura, Y; Toma, I; Metzler, S; Kooreman, NG; Ramunas, J; Holbrook, C; McConnell, MV; Blau, H; Harnish, P; Rulifson, E; Yang, PC (2015) Direct Evaluation of Myocardial Viability and Stem Cell Engraftment Demonstrates Salvage of the Injured Myocardium. Circ Res 116:E40-E50. doi:10.1161/CIRCRESAHA.116.304668

302. Riegler, J; Tiburcy, M; Ebert, A; Tzatzalos, E; Raaz, U; Abilez, OJ; Shen, Q; Kooreman, NG; Neofytou, E; Chen, VC; Wang, M; Meyer, T; Tsao, PS; Connolly, AJ; Couture, LA; Gold, JD; Zimmermann, WH; Wu, JC (2015) Human Engineered Heart Muscles Engraft and Survive Long Term in a Rodent Myocardial Infarction Model. Circ Res 117:720-730. doi:10.1161/CIRCRESAHA.115.306985

303. Skyschally, A; Gent, S; Amanakis, G; Schulte, C; Kleinbongard, P; Heusch, G (2015) Across-Species Transfer of Protection by Remote Ischemic Preconditioning With Species-Specific Myocardial Signal Transduction by Reperfusion Injury Salvage Kinase and Survival Activating Factor Enhancement Pathways. Circ Res 117:279-288. doi:10.1161/CIRCRESAHA.117.306878

304. Van Aelst, LNL; Voss, S; Carai, P; Van Leeuwen, R; Vanhoutte, D; Sanders-van Wijk, S; Eurlings, L; Swinnen, M; Verheyen, FK; Verbeken, E; Nef, H; Troidl, C; Cook, SA; Brunner-La Rocca, HP; Mollmann, H; Papageorgiou, AP; Heymans, S (2015) Osteoglycin Prevents Cardiac Dilatation and Dysfunction After Myocardial Infarction Through Infarct Collagen Strengthening. Circ Res 116:425-436. doi:10.1161/CIRCRESAHA.116.304599

305. Yang, YF; Cheng, HW; Qiu, YL; Dupee, D; Noonan, M; Lin, YD; Fisch, S; Unno, K; Sereti, KI; Liao, RL (2015) MicroRNA-34a Plays a Key Role in Cardiac Repair and Regeneration Following Myocardial Infarction. Circ Res 117:450-459. doi:10.1161/CIRCRESAHA.117.305962

306. Zhu, M; Goetsch, SC; Wang, ZN; Luo, R; Hill, JA; Schneider, J; Morris, SM; Liu, ZP (2015) FoxO4 Promotes Early Inflammatory Response Upon Myocardial Infarction via Endothelial Arg1. Circ Res 117:967-977. doi:10.1161/CIRCRESAHA.115.306919

307. Chen, W; Spitzl, A; Mathes, D; Nikolaev, VO; Werner, F; Weirather, J; Spiranec, K; Rock, K; Fischer, JW; Kammerer, U; Stegner, D; Baba, HA; Hofmann, U; Frantz, S; Kuhn, M (2016) Endothelial Actions of ANP Enhance Myocardial Inflammatory Infiltration in the Early Phase After Acute Infarction. Circ Res 119:237-248. doi:10.1161/CIRCRESAHA.115.307196

308. Polhemus, DJ; Gao, J; Scarborough, AL; Trivedi, R; McDonough, KH; Goodchild, TT; Smart, F; Kapusta, DR; Lefer, DJ (2016) Radiofrequency Renal Denervation Protects the Ischemic Heart via Inhibition of GRK2 and Increased Nitric Oxide Signaling. Circ Res 119:470-480. doi:10.1161/CIRCRESAHA.115.308278

309. Tang, XL; Li, QH; Rokosh, G; Sanganalmath, SK; Chen, N; Ou, QH; Stowers, H; Hunt, G; Bolli, R (2016) Long-Term Outcome of Administration of c-kit(POS) Cardiac Progenitor Cells After Acute Myocardial Infarction Transplanted Cells Do not Become Cardiomyocytes, but Structural and Functional Improvement and Proliferation of Endogenous Cells Persist for at Least One Year. Circ Res 118:1091-1105. doi:10.1161/CIRCRESAHA.115.307647

310. Woodall, MC; Woodall, BP; Gao, EH; Yuan, A; Koch, WJ (2016) Cardiac Fibroblast GRK2 Deletion Enhances Contractility and Remodeling Following Ischemia/Reperfusion Injury. Circ Res 119:1116-1127. doi:10.1161/CIRCRESAHA.116.309538

311. Cao, Y; Bojjireddy, N; Kim, M; Li, T; Zhai, PY; Nagarajan, N; Sadoshima, J; Palmiter, RD; Tian, R (2017) Activation of gamma 2-AMPK Suppresses Ribosome Biogenesis and Protects Against Myocardial Ischemia/Reperfusion Injury. Circ Res 121:1182-1191. doi:10.1161/CIRCRESAHA.117.311159

312. DeBerge, M; Yeap, XY; Dehn, S; Zhang, S; Grigoryeva, L; Misener, S; Procissi, D; Zhou, X; Lee, DC; Muller, WA; Luo, XR; Rothlin, C; Tabas, I; Thorp, EB (2017) MerTK Cleavage on Resident Cardiac Macrophages Compromises Repair After Myocardial Ischemia Reperfusion Injury. Circ Res 121:930-940. doi:10.1161/CIRCRESAHA.117.311327

313. Fazal, L; Laudette, M; Paula-Gomes, S; Pons, S; Conte, C; Tortosa, F; Sicard, P; Sainte-Marie, Y; Bisserier, M; Lairez, O; Lucas, A; Roy, J; Ghaleh, B; Fauconnier, J; Mialet-Perez, J; Lezoualc'h, F (2017) Multifunctional Mitochondrial Epac1 Controls Myocardial Cell Death. Circ Res 120:645-657. doi:10.1161/CIRCRESAHA.116.309859

314. Fernandez-Jimenez, R; Galan-Arriola, C; Sanchez-Gonzalez, J; Aguero, J; Lopez-Martin, GJ; Gomez-Talavera, S; Garcia-Prieto, J; Benn, A; Molina-Iracheta, A; Barreiro-Perez, M; Martin-Garcia, A; Garcia-Lunar, I; Pizarro, G; Sanz, J; Sanchez, PL; Fuster, V; Ibanez, B (2017) Effect of Ischemia Duration and Protective Interventions on the Temporal Dynamics of Tissue Composition After Myocardial Infarction. Circ Res 121:439-450. doi:10.1161/CIRCRESAHA.117.310901

315. Gao, L; Kupfer, ME; Jung, JP; Yang, LB; Zhang, P; Sie, YD; Tran, Q; Ajeti, V; Freeman, BT; Fast, VG; Campagnola, PJ; Ogle, BM; Zhang, JY (2017) Myocardial Tissue Engineering With Cells Derived From Human-Induced Pluripotent Stem Cells and a Native-Like, High-Resolution, 3-Dimensionally Printed Scaffold. Circ Res 120:1318-1325. doi:10.1161/CIRCRESAHA.116.310277

316. Lesizza, P; Prosdocimo, G; Martinelli, V; Sinagra, G; Zacchigna, S; Giacca, M (2017) Single-Dose Intracardiac Injection of Pro-Regenerative MicroRNAs Improves Cardiac Function After Myocardial Infarction. Circ Res 120:1298-1304. doi:10.1161/CIRCRESAHA.116.309589

317. Luger, D; Lipinski, MJ; Westman, PC; Glover, DK; Dimastromatteo, J; Frias, JC; Albelda, MT; Sikora, S; Kharazi, A; Vertelov, G; Waksman, R; Epstein, SE (2017) Intravenously Delivered Mesenchymal Stem Cells Systemic Anti-Inflammatory Effects Improve Left Ventricular Dysfunction in Acute Myocardial Infarction and Ischemic Cardiomyopathy. Circ Res 120:1598-1613. doi:10.1161/CIRCRESAHA.117.310599

318. Luo, L; Tang, JN; Nishi, K; Yan, C; Dinh, PU; Cores, J; Kudo, T; Zhang, JY; Li, TS; Cheng, K (2017) Fabrication of Synthetic Mesenchymal Stem Cells for the Treatment of Acute Myocardial Infarction in Mice. Circ Res 120:1768-1775. doi:10.1161/CIRCRESAHA.116.310374

319. Sharp, TE; Schena, GJ; Hobby, AR; Starosta, T; Berretta, RM; Wallner, M; Borghetti, G; Gross, P; Yu, DH; Johnson, J; Feldsott, E; Trappanese, DM; Toib, A; Rabinowitz, JE; George, JC; Kubo, H; Mohsin, S; Houser, SR (2017) Cortical Bone Stem Cell Therapy Preserves Cardiac Structure and Function After Myocardial Infarction. Circ Res 121:1263-1278. doi:10.1161/CIRCRESAHA.117.311174

320. Su, SA; Yang, D; Wu, Y; Xie, Y; Zhu, W; Cai, ZJ; Shen, J; Fu, ZR; Wang, YP; Jia, LL; Wang, YD; Wang, JA; Xiang, MX (2017) EphrinB2 Regulates Cardiac Fibrosis Through Modulating the Interaction of Stat3 and TGF-beta/Smad3 Signaling. Circ Res 121:617-627. doi:10.1161/CIRCRESAHA.117.311045

321. Tachibana, A; Santoso, MR; Mahmoudi, M; Shukla, P; Wang, L; Bennett, M; Goldstone, AB; Wang, M; Fukushi, M; Ebert, AD; Woo, YJ; Rulifson, E; Yang, PC (2017) Paracrine Effects of the Pluripotent Stem Cell-Derived Cardiac Myocytes Salvage the Injured Myocardium. Circ Res 121:E22-E36. doi:10.1161/CIRCRESAHA.117.310803

322. Yan, XX; Zhang, H; Fan, Q; Hu, J; Tao, R; Chen, QJ; Iwakura, Y; Shen, WF; Lu, L; Zhang, Q; Zhang, RY (2017) Dectin-2 Deficiency Modulates Th1 Differentiation and Improves Wound Healing After Myocardial Infarction. Circ Res 120:1116-1129. doi:10.1161/CIRCRESAHA.116.310260

323. Casin, KM; Fallica, J; Mackowski, N; Veenema, RJ; Chan, A; St Paul, A; Zhu, GS; Bedja, D; Biswal, S; Kohr, MJ (2018) S-Nitrosoglutathione Reductase Is Essential for Protecting the Female Heart From Ischemia-Reperfusion Injury. Circ Res 123:1232-1243. doi:10.1161/CIRCRESAHA.118.313956

324. Kleinbongard, P; Amanakis, G; Skyschally, A; Heusch, G (2018) Reflection of Cardioprotection by Remote Ischemic Perconditioning in Attenuated ST-Segment Elevation During Ongoing Coronary Occlusion in Pigs Evidence for Cardioprotection From Ischemic Injury. Circ Res 122:1102-1108. doi:10.1161/CIRCRESAHA.118.312784

325. Lieder, HR; Kleinbongard, P; Skyschally, A; Hagelschuer, H; Chilian, WM; Heusch, G (2018) Vago-Splenic Axis in Signal Transduction of Remote Ischemic Preconditioning in Pigs and Rats. Circ Res 123:1152-1163. doi:10.1161/CIRCRESAHA.118.313859

326. Miyazaki, T; Otani, K; Chiba, A; Nishimura, H; Tokudome, T; Takano-Watanabe, H; Matsuo, A; Ishikawa, H; Shimamoto, K; Fukui, H; Kanai, Y; Yasoda, A; Ogata, S; Nishimura, K; Minamino, N; Mochizuki, N (2018) A New Secretory Peptide of Natriuretic Peptide Family, Osteocrin, Suppresses the Progression of Congestive Heart Failure After Myocardial Infarction. Circ Res 122:742-751. doi:10.1161/CIRCRESAHA.117.312624

327. Otaka, N; Shibata, R; Ohashi, K; Uemura, Y; Kambara, T; Enomoto, T; Ogawa, H; Ito, M; Kawanishi, H; Maruyama, S; Joki, Y; Fujikawa, Y; Narita, S; Unno, K; Kawamoto, Y; Murate, T; Murohara, T; Ouchi, N (2018) Myonectin Is an Exercise-Induced Myokine That Protects the Heart From Ischemia-Reperfusion Injury. Circ Res 123:1326-1338. doi:10.1161/CIRCRESAHA.118.313777

328. Techiryan, G; Weil, BR; Palka, BA; Canty, JM (2018) Effect of Intracoronary Metformin on Myocardial Infarct Size in Swine. Circ Res 123:986-995. doi:10.1161/CIRCRESAHA.118.313341

329. Xiao, CC; Wang, K; Xu, YC; Hu, HX; Zhang, N; Wang, YC; Zhong, ZW; Zhao, J; Li, QJ; Zhu, D; Ke, CL; Zhong, SH; Wu, XP; Yu, H; Zhu, W; Chen, JH; Zhang, JY; Wang, JA; Hu, XY (2018) Transplanted Mesenchymal Stem Cells Reduce Autophagic Flux in Infarcted Hearts via the Exosomal Transfer of miR-125b. Circ Res 123:564-578. doi:10.1161/CIRCRESAHA.118.312758

330. Xu, SY; Tao, J; Yang, L; Zhang, E; Boriboun, C; Zhou, JL; Sun, TJ; Cheng, M; Huang, K; Shi, JW; Dong, NG; Liu, QH; Zhao, TC; Qiu, HY; Harris, RA; Chandel, NS; Losordo, DW; Qin, GJ (2018) E2F1 Suppresses Oxidative Metabolism and Endothelial Differentiation of Bone Marrow Progenitor Cells. Circ Res 122:701-711. doi:10.1161/CIRCRESAHA.117.311814

331. Yamada, Y; Wakao, S; Kushida, Y; Minatoguchi, S; Mikami, A; Higashi, K; Baba, S; Shigemoto, T; Kuroda, Y; Kanamori, H; Amin, M; Kawasaki, M; Nishigaki, K; Taoka, M; Isobe, T; Muramatsu, C; Dezawa, M; Minatoguchi, S (2018) S1P-S1PR2 Axis Mediates Homing of Muse Cells Into Damaged Heart for Long-Lasting Tissue Repair and Functional Recovery After Acute Myocardial Infarction. Circ Res 122:1069-1083. doi:10.1161/CIRCRESAHA.117.311648

332. Zhu, WQ; Zhao, M; Mattapally, S; Chen, SF; Zhang, JY (2018) CCND2 Overexpression Enhances the Regenerative Potency of Human Induced Pluripotent Stem Cell-Derived Cardiomyocytes: Remuscularization of Injured Ventricle. Circ Res 122:88-96. doi:10.1161/CIRCRESAHA.117.311504

333. Borden, A; Kurian, J; Nickoloff, E; Yang, YJ; Troupes, CD; Ibetti, J; Lucchese, AM; Gao, E; Mohsin, S; Koch, WJ; Houser, SR; Kishore, R; Khan, M (2019) Transient Introduction of miR-294 in the Heart Promotes Cardiomyocyte Cell Cycle Reentry After Injury. Circ Res 125:14-25. doi:10.1161/CIRCRESAHA.118.314223

334. Hou, ZX; Qin, XH; Hu, YY; Zhang, X; Li, GH; Wu, J; Li, J; Sha, JD; Chen, JW; Xia, JL; Wang, LF; Gao, F (2019) Longterm Exercise-Derived Exosomal miR-342-5p A Novel Exerkine for Cardioprotection. Circ Res 124:1386-1400. doi:10.1161/CIRCRESAHA.118.314635

335. Jia, DL; Jiang, H; Weng, XY; Wu, J; Bai, PY; Yang, WL; Wang, Z; Hu, K; Sun, AJ; Ge, JB (2019) Interleukin-35 Promotes Macrophage Survival and Improves Wound Healing After Myocardial Infarction in Mice. Circ Res 124:1323-1336. doi:10.1161/CIRCRESAHA.118.314569

336. Korf-Klingebiel, M; Reboll, MR; Grote, K; Schleiner, H; Wang, Y; Wu, XK; Klede, S; Mikhed, Y; Bauersachs, J; Klintschar, M; Rudat, C; Kispert, A; Niessen, HW; Luke, T; Dierks, T; Wollert, KC (2019) Heparan Sulfate-Editing Extracellular Sulfatases Enhance VEGF Bioavailability for Ischemic Heart Repair. Circ Res 125:787-801. doi:10.1161/CIRCRESAHA.119.315023

337. Petz, A; Grandoch, M; Gorski, DJ; Abrams, M; Piroth, M; Schneckmann, R; Homann, S; Muller, J; Hartwig, S; Lehr, S; Yamaguchi, Y; Wight, TN; Gorressen, S; Ding, ZP; Kotter, S; Kruger, M; Heinen, A; Kelm, M; Godecke, A; Flogel, U; Fischer, JW (2019) Cardiac Hyaluronan Synthesis Is Critically Involved in the Cardiac Macrophage Response and Promotes Healing After Ischemia Reperfusion Injury. Circ Res 124:1433-1447. doi:10.1161/CIRCRESAHA.118.313285

338. Trincot, CE; Xu, WJ; Zhang, H; Kulikauskas, MR; Caranasos, TG; Jensen, BC; Sabine, A; Petrova, TV; Caron, KM (2019) Adrenomedullin Induces Cardiac Lymphangiogenesis After Myocardial Infarction and Regulates Cardiac Edema Via Connexin 43. Circ Res 124:101-113. doi:10.1161/CIRCRESAHA.118.313835

339. Zhao, L; Cheng, GM; Choksi, K; Samanta, A; Girgis, M; Soder, R; Vincent, RJ; Wulser, M; De Ruyter, M; McEnulty, P; Hauptman, J; Yang, YJ; Weiner, CP; Dawn, B (2019) Transplantation of Human Umbilical Cord Blood-Derived Cellular Fraction Improves Left Ventricular Function and Remodeling After Myocardial Ischemia/Reperfusion. Circ Res 125:759-772. doi:10.1161/CIRCRESAHA.119.315216

340. Gu, SS; Tan, JL; Li, Q; Liu, SY; Ma, J; Zheng, YJ; Liu, JL; Bi, W; Sha, P; Li, XX; Wei, M; Cao, N; Yang, HT (2020) Downregulation of LAPTM4B Contributes to the Impairment of the Autophagic Flux via Unopposed Activation of mTORC1 Signaling During Myocardial Ischemia/Reperfusion Injury. Circ Res 127:E148-E165. doi:10.1161/CIRCRESAHA.119.316388

341. Marinkovic, G; Koenis, DS; de Camp, L; Jablonowski, R; Graber, N; de Waard, V; de Vries, CJ; Goncalves, I; Nilsson, J; Jovinge, S; Schiopu, A (2020) S100A9 Links Inflammation and Repair in Myocardial Infarction. Circ Res 127:664-676. doi:10.1161/CIRCRESAHA.120.315865

342. Yan, WJ; Lin, C; Guo, YZ; Chen, YH; Du, YH; Lau, WB; Xia, YL; Zhang, FY; Su, RZ; Gao, EH; Wang, YJ; Li, CY; Liu, R; Ma, XL; Tao, L (2020) N-Cadherin Overexpression Mobilizes the Protective Effects of Mesenchymal Stromal Cells Against Ischemic Heart Injury Through a beta-Catenin-Dependent Manner. Circ Res 126:857-874. doi:10.1161/CIRCRESAHA.119.315806

343. Yue, YJ; Wang, CL; Benedict, C; Huang, GC; Truongcao, M; Roy, R; Cimini, M; Garikipati, VNS; Cheng, ZJ; Koch, WJ; Kishore, R (2020) Interleukin-10 Deficiency Alters Endothelial Progenitor Cell-Derived Exosome Reparative Effect on Myocardial Repair via Integrin-Linked Kinase Enrichment. Circ Res 126:315-329. doi:10.1161/CIRCRESAHA.119.315829

344. Burke, RM; Dirkx, RA; Quijada, P; Lighthouse, JK; Mohan, A; O'Brien, M; Wojciechowski, W; Woeller, CF; Phipps, RP; Alexis, JD; Ashton, JM; Small, EM (2021) Prevention of Fibrosis and Pathological Cardiac Remodeling by Salinomycin. Circ Res 128:1663-1678. doi:10.1161/CIRCRESAHA.120.317791

345. Gadi, I; Fatima, S; Elwakiel, A; Nazir, S; Al-Dabet, MM; Rana, R; Bock, F; Manoharan, J; Gupta, D; Biemann, R; Nieswandt, B; Braun-Dullaeus, R; Besler, C; Scholz, M; Geffers, R; Griffin, JH; Esmon, CT; Kohli, S; Isermann, B; Shahzad, K (2021) Different DOACs Control Inflammation in Cardiac Ischemia-Reperfusion Differently. Circ Res 128:513-529. doi:10.1161/CIRCRESAHA.120.317219

346. Li, J; Salvador, AM; Li, GP; Valkov, N; Ziegler, O; Yeri, A; Xiao, CY; Meechoovet, B; Alsop, E; Rodosthenous, RS; Kundu, P; Huan, TX; Levy, D; Tigges, J; Pico, AR; Ghiran, I; Silverman, MG; Meng, XM; Kitchen, R; Xu, JH; Van Keuren-Jensen, K; Shah, R; Xiao, JJ; Das, S (2021) Mir-30d Regulates Cardiac Remodeling by Intracellular and Paracrine Signaling. Circ Res 128:E1-E23. doi:10.1161/CIRCRESAHA.120.317244

347. Liu, MM; Yan, M; He, JL; Lv, HZ; Chen, ZP; Peng, LY; Cai, WB; Yao, F; Chen, C; Shi, L; Zhang, K; Zhang, X; Wang, DW; Wang, L; Zhu, Y; Ai, D (2021) Macrophage MST1/2 Disruption Impairs Post-Infarction Cardiac Repair via LTB4. Circ Res 129:909-926. doi:10.1161/CIRCRESAHA.121.319687

348. Patil, M; Saheera, S; Dubey, PK; Kahn-Krell, A; Govindappa, PK; Singh, S; Tousif, S; Zhang, QK; Lal, H; Zhang, JY; Qin, GJ; Krishnamurthy, P (2021) Novel Mechanisms of Exosome-Mediated Phagocytosis of Dead Cells in Injured Heart. Circ Res 129:1006-1020. doi:10.1161/CIRCRESAHA.120.317900

349. Shi, HR; Gao, Y; Dong, Z; Yang, JE; Gao, RF; Li, X; Zhang, SQ; Ma, LL; Sun, XL; Wang, Z; Zhang, F; Hu, K; Sun, AJ; Ge, JB (2021) GSDMD-Mediated Cardiomyocyte Pyroptosis Promotes Myocardial I/R Injury. Circ Res 129:383-396. doi:10.1161/CIRCRESAHA.120.318629

350. Song, Y; Xu, CJ; Liu, JF; Li, YL; Wang, H; Shan, D; Wainer, IW; Hu, XL; Zhang, Y; Woo, AYH; Xiao, RP (2021) Heterodimerization With 5-HT2BR Is Indispensable for beta(2)AR-Mediated Cardioprotection. Circ Res 128:262-277. doi:10.1161/CIRCRESAHA.120.317011

351. Xie, M; Cho, GW; Kong, YL; Li, DL; Altamirano, F; Luo, X; Morales, CR; Jiang, N; Schiattarella, GG; May, HI; Medina, J; Shelton, JM; Ferdous, A; Gillette, TG; Hill, JA (2021) Activation of Autophagic Flux Blunts Cardiac Ischemia/Reperfusion Injury. Circ Res 129:435-450. doi:10.1161/CIRCRESAHA.120.318601

352. Zhang, QJ; He, Y; Li, YN; Shen, HL; Lin, L; Zhu, M; Wang, ZN; Luo, X; Hill, JA; Cao, D; Luo, RL; Zou, R; McAnally, J; Liao, J; Bajona, P; Zang, QS; Yu, YH; Liu, ZP (2021) Matricellular Protein Cilp1 Promotes Myocardial Fibrosis in Response to Myocardial Infarction. Circ Res 129:1021-1035. doi:10.1161/CIRCRESAHA.121.319482

353. He, HM; Mulhern, RM; Oldham, WM; Xiao, WS; Lin, YD; Liao, R; Loscalzo, J (2022) L-2-Hydroxyglutarate Protects Against Cardiac Injury via Metabolic Remodeling. Circ Res 131:562-579. doi:10.1161/CIRCRESAHA.122.321227

354. Lv, FX; Wang, YF; Shan, D; Guo, SL; Chen, GJ; Jin, L; Zheng, W; Feng, H; Zeng, XH; Zhang, S; Zhang, Y; Hu, XL; Xiao, RP (2022) Blocking MG53(S255) Phosphorylation Protects Diabetic Heart From Ischemic Injury. Circ Res 131:962-976. doi:10.1161/CIRCRESAHA.122.321055

355. Nakayama, Y; Mukai, N; Kreitzer, G; Patwari, P; Yoshioka, J (2022) Interaction of ARRDC4 With GLUT1 Mediates Metabolic Stress in the Ischemic Heart. Circ Res 131:510-527. doi:10.1161/CIRCRESAHA.122.321351

356. Ni, C; Chen, YJ; Xu, YC; Zhao, J; Li, QJ; Xiao, CC; Wu, Y; Wang, JY; Wang, YC; Zhong, ZW; Zhang, L; Wu, RR; Liu, QN; Wu, XP; Ke, CL; Zhu, W; Chen, JH; Huang, JJ; Wang, YB; Wang, JN; Hu, XY (2022) FMO2 (Flavin Containing Monooxygenase 2) Prevents Cardiac Fibrosis via CYP2J3-SMURF2 Axis. Circ Res 131:189-206. doi:10.1161/CIRCRESAHA.122.320538

357. Pei, JQ; Cai, L; Wang, F; Xu, CS; Pei, SQ; Guo, HW; Sun, XG; Chun, J; Cong, XF; Zhu, WQ; Zheng, Z; Chen, X (2022) LPA(2) Contributes to Vascular Endothelium Homeostasis and Cardiac Remodeling After Myocardial Infarction. Circ Res 131:388-403. doi:10.1161/CIRCRESAHA.122.321036

358. Prag, HA; Aksentijevic, D; Dannhorn, A; Giles, AV; Mulvey, JF; Sauchanka, O; Du, LP; Bates, G; Reinhold, J; Kula-Alwar, D; Xu, ZL; Pellerin, L; Goodwin, RJA; Murphy, MP; Krieg, T (2022) Ischemia-Selective Cardioprotection by Malonate for Ischemia/Reperfusion Injury. Circ Res 131:528-541. doi:10.1161/CIRCRESAHA.121.320717

359. Wan, Q; Xu, CS; Zhu, LY; Zhang, YZ; Peng, ZK; Chen, H; Rao, HJ; Zhang, EL; Wang, HY; Chu, F; Ning, X; Yang, XJ; Yuan, JQ; Wu, YJ; Huang, Y; Hu, SS; Liu, DP; Wang, M (2022) Targeting PDE4B (Phosphodiesterase-4 Subtype B) for Cardioprotection in Acute Myocardial Infarction via Neutrophils and Microcirculation. Circ Res 131:442-455. doi:10.1161/CIRCRESAHA.122.321365

360. Xiao, HW; Zhang, MY; Wu, H; Wu, JX; Hu, XX; Pei, XY; Li, DY; Zhao, L; Hua, Q; Meng, B; Zhang, XW; Peng, LL; Cheng, XL; Li, ZY; Yang, WQ; Zhang, Q; Zhang, Y; Lu, YJ; Pan, ZW (2022) CIRKIL Exacerbates Cardiac Ischemia/Reperfusion Injury by Interacting With Ku70. Circ Res 130:E3-E17. doi:10.1161/CIRCRESAHA.121.318992

361. Young, A; Bradley, LA; Farrar, E; Bilcheck, HO; Tkachenko, S; Saucerman, JJ; Bekiranov, S; Wolf, MJ (2022) Inhibition of DYRK1a Enhances Cardiomyocyte Cycling After Myocardial Infarction. Circ Res 130:1345-1361. doi:10.1161/CIRCRESAHA.121.320005

362. Zhao, H; Chen, XY; Hu, GY; Li, CY; Guo, LY; Zhang, L; Sun, FF; Xia, YL; Yan, WJ; Cui, Z; Guo, YZ; Guo, X; Huang, C; Fan, MM; Wang, S; Zhang, FY; Tao, L (2022) Small Extracellular Vesicles From Brown Adipose Tissue Mediate Exercise Cardioprotection. Circ Res 130:1490-1506. doi:10.1161/CIRCRESAHA.121.320458

363. Cannavo, A; Jun, SH; Rengo, G; Marzano, F; Agrimi, J; Liccardo, D; Elia, A; Keceli, G; Altobelli, GG; Marcucci, L; Megighian, A; Gao, ER; Feng, N; Kammers, K; Ferrara, N; FiNOS, L; Koch, WJ; Paolocci, N (2023) & beta;3AR-Dependent Brain-Derived Neurotrophic Factor (BDNF) Generation Limits Chronic Postischemic Heart Failure. Circ Res 132:867-881. doi:10.1161/CIRCRESAHA.122.321583

364. Enzan, N; Matsushima, S; Ikeda, S; Okabe, K; Ishikita, A; Yamamoto, T; Sada, M; Miyake, R; Tsutsui, Y; Nishimura, R; Toyohara, T; Ikeda, Y; Shojima, Y; Miyamoto, HD; Tadokoro, T; Ikeda, M; Abe, K; Ide, T; Kinugawa, S; Tsutsui, H (2023) ZBP1 Protects Against mtDNA-Induced Myocardial Inflammation in Failing Hearts. Circ Res 132:1110-1126. doi:10.1161/CIRCRESAHA.122.322227

365. Hume, RD; Kanagalingam, S; Deshmukh, T; Chen, S; Mithieux, SM; Rashid, FN; Roohani, I; Lu, J; Doan, T; Graham, D; Clayton, ZE; Slaughter, E; Kizana, E; Stempien-Otero, AS; Brown, P; Thomas, L; Weiss, AS; Chong, JJH (2023) Tropoelastin Improves Post-Infarct Cardiac Function. Circ Res 132:72-86. doi:10.1161/CIRCRESAHA.122.321123

366. Ichihara, G; Katsumata, Y; Sugiura, Y; Matsuoka, Y; Maeda, R; Endo, J; Anzai, A; Shirakawa, K; Moriyama, H; Kitakata, H; Hiraide, T; Goto, S; Ko, S; Iwasawa, Y; Sugai, K; Daigo, K; Goto, S; Sato, K; Yamada, K; Suematsu, M; Ieda, M; Sano, M (2023) MRP1-Dependent Extracellular Release of Glutathione Induces Cardiomyocyte Ferroptosis After Ischemia-Reperfusion. Circ Res 133:861-876. doi:10.1161/CIRCRESAHA.123.323517

367. Marti-Pamies, I; Thoonen, R; Morley, M; Graves, L; Tamez, J; Caplan, A; McDaid, K; Yao, VC; Hindle, A; Gerszten, RE; Farrell, LA; Li, L; Tseng, YH; Profeta, G; Buys, ES; Bloch, DB; Scherrer-Crosbie, M (2023) Brown Adipose Tissue and BMP3b Decrease Injury in Cardiac Ischemia-Reperfusion. Circ Res 133:353-365. doi:10.1161/CIRCRESAHA.122.322337

368. Schutte, JP; Manke, MC; Hemmen, K; Munzer, P; Schorg, BF; Ramos, GC; Pogoda, M; Dicenta, V; Hoffmann, SHL; Pinnecker, J; Kollotzek, F; Zdanyte, M; Mueller, KAL; Singh, Y; Mack, AF; Pichler, B; Lang, F; Nieswandt, B; Gawaz, M; Heinze, KG; Casadei, N; Borst, O (2023) Platelet-Derived MicroRNAs Regulate Cardiac Remodeling After Myocardial Ischemia. Circ Res 132:E96-E113. doi:10.1161/CIRCRESAHA.122.322459

369. Sun, JC; Wang, L; Matthews, RC; Walcott, GP; Lu, YA; Wei, YH; Zhou, Y; Zangi, L; Zhang, JY (2023) CCND2 Modified mRNA Activates Cell Cycle of Cardiomyocytes in Hearts With Myocardial Infarction in Mice and Pigs. Circ Res 133:484-504. doi:10.1161/CIRCRESAHA.123.322929

370. Sun, YX; Ma, MQ; Cao, DD; Zheng, AC; Zhang, YY; Su, Y; Wang, JF; Xu, YH; Zhou, M; Tang, YS; Liu, YF; Ma, T; Fan, AY; Zhang, XY; Zhu, QL; Qin, JC; Mo, CY; Xu, YW; Zhang, L; Xu, DC; Yue, R (2023) Inhibition of Fap Promotes Cardiac Repair by Stabilizing BNP. Circ Res 132:586-600. doi:10.1161/CIRCRESAHA.122.320781

371. Yang, Y; Zhang, Y; Yang, JQ; Zhang, MM; Tian, T; Jiang, Y; Liu, XN; Xue, GL; Li, XD; Zhang, XF; Li, SX; Huang, X; Li, Z; Guo, Y; Zhao, LX; Bao, HR; Zhou, ZW; Song, JH; Yang, GH; Xuan, LA; Shan, HL; Zhang, ZR; Lu, YJ; Yang, BF; Pan, ZW (2023) Interdependent Nuclear Co-Trafficking of ASPP1 and p53 Aggravates Cardiac Ischemia/Reperfusion Injury. Circ Res 132:208-222. doi:10.1161/CIRCRESAHA.122.321153
